# Rhinovirus-induced epithelial RIG-I inflammasome suppresses antiviral immunity and promotes inflammation in asthma and COVID-19

**DOI:** 10.1038/s41467-023-37470-4

**Published:** 2023-04-22

**Authors:** Urszula Radzikowska, Andrzej Eljaszewicz, Ge Tan, Nino Stocker, Anja Heider, Patrick Westermann, Silvio Steiner, Anita Dreher, Paulina Wawrzyniak, Beate Rückert, Juan Rodriguez-Coira, Damir Zhakparov, Mengting Huang, Bogdan Jakiela, Marek Sanak, Marcin Moniuszko, Liam O’Mahony, Marek Jutel, Tatiana Kebadze, David J. Jackson, Michael R. Edwards, Volker Thiel, Sebastian L. Johnston, Cezmi A. Akdis, Milena Sokolowska

**Affiliations:** 1 Swiss Institute of Allergy and Asthma Research (SIAF), University of Zurich, Herman-Burchard-Strasse 9, 7265 Davos Wolfgang, Switzerland; 2 Christine Kühne – Center for Allergy Research and Education (CK-CARE), Herman-Burchard-Strasse 1, 7265 Davos Wolfgang, Switzerland; 3 Department of Regenerative Medicine and Immune Regulation, Medical University of Bialystok, Waszyngtona 13 Str., 15-269 Bialystok, Poland; 4 Functional Genomics Center Zurich, ETH Zurich/University of Zurich, Winterthurerstrasse 190, 8057 Zurich, Switzerland; 5 Institute of Virology and Immunology (IVI), Laenggassstrasse 122, 3012 Bern, Switzerland; 6 Department of Infectious Diseases and Pathobiology, Vetsuisse Faculty, University of Bern, Laenggassstrasse 122, 3012 Bern, Switzerland; 7 Graduate School for Cellular and Biomedical Sciences, University of Bern, Mittelstrasse 43, 3012 Bern, Switzerland; 8 Division of Clinical Chemistry and Biochemistry, University Children’s Hospital Zurich, Raemistrasse 100, 8091 Zurich, Switzerland; 9 Children’s Research Center, University Children’s Hospital Zurich, Raemistrasse 100, 8091 Zurich, Switzerland; 10 IMMA, Department of Basic Medical Sciences, Facultad de Medicina, Universidad San Pablo-CEU, CEU Universities Madrid, C. de Julian Romea 23, 28003 Madrid, Spain; 11 Centre for Metabolomics and Bioanalysis (CEMBIO), Department of Chemistry and Biochemistry, Facultad de Farmacia, Universidad San Pablo-CEU, CEU Universities Madrid, Urb. Monteprincipe 28925, Alcorcon, Madrid Spain; 12 Department of Internal Medicine, Jagiellonian University Medical College, M. Skawinska 8 Str., 31-066 Krakow, Poland; 13 Department of Allergology and Internal Medicine, Medical University of Bialystok, M. Sklodowskiej-Curie 24A Str., 15-276 Bialystok, Poland; 14 Department of Medicine and School of Microbiology, APC Microbiome Ireland, University College Cork, College Rd, T12 E138 Cork, Ireland; 15 Department of Clinical Immunology, Wroclaw Medical University, wyb. Lidwika Pasteura 1 Str, 50-367 Wroclaw, Poland; 16 ALL-MED Medical Research Institute, Gen. Jozefa Hallera 95 Str., 53-201 Wroclaw, Poland; 17 National Heart and Lung Institute, Imperial College London, Guy Scadding Building, Cale Street, London, SW3 6LY UK; 18 Department of Infectious Diseases, Imperial College London, School of Medicine, St Mary’s Hospital, Praed Street, London, W21NY UK; 19 Guy’s Severe Asthma Centre, School of Immunology & Microbial Sciences, King’s College London, Strand, London, WC2R 2LS UK; 20 Guy’s & St Thomas’ NHS Trust, St Thomas‘ Hospital, Westminster Bridge Rd, London, SE1 7EH UK; 21 Asthma UK Centre in Allergic Mechanisms of Asthma, Norfolk Place, London, W2 1PG UK; 22 Multidisciplinary Center for Infectious Diseases, University of Bern, Hallerstrasse 6, 3012 Bern, Switzerland; 23 Imperial College Healthcare HNS Trust, The Bays, S Wharf Rd, London, W2 1NY UK

**Keywords:** Viral infection, Asthma, RIG-I-like receptors, Mucosal immunology, Infection

## Abstract

Rhinoviruses and allergens, such as house dust mite are major agents responsible for asthma exacerbations. The influence of pre-existing airway inflammation on the infection with severe acute respiratory syndrome coronavirus 2 (SARS-CoV-2) is largely unknown. We analyse mechanisms of response to viral infection in experimental in vivo rhinovirus infection in healthy controls and patients with asthma, and in in vitro experiments with house dust mite, rhinovirus and SARS-CoV-2 in human primary airway epithelium. Here, we show that rhinovirus infection in patients with asthma leads to an excessive RIG-I inflammasome activation, which diminishes its accessibility for type I/III interferon responses, leading to their early functional impairment, delayed resolution, prolonged viral clearance and unresolved inflammation in vitro and in vivo. Pre-exposure to house dust mite augments this phenomenon by inflammasome priming and auxiliary inhibition of early type I/III interferon responses. Prior infection with rhinovirus followed by SARS-CoV-2 infection augments RIG-I inflammasome activation and epithelial inflammation. Timely inhibition of the epithelial RIG-I inflammasome may lead to more efficient viral clearance and lower the burden of rhinovirus and SARS-CoV-2 infections.

## Abstract (web summary)

Viral infections and exposure to inhaled allergens are linked to asthma onset, exacerbations and progression. Here, the authors used controlled experimental rhinovirus infection in patients with and without asthma, and further assessed in vitro the role of house dust mite allergen combined with rhinovirus and SARS-CoV-2 infection. They discovered that rhinovirus-induced activation of epithelial RIG-I inflammasome supresses antiviral immunity, promotes inflammation during asthma exacerbations and aggravates subsequent infection with SARS-CoV-2, particularly upon house dust mite exposure.

## Introduction

Asthma is one of the most common chronic inflammatory lung diseases affecting more than 5% of the global population^1^. Its pathogenesis and clinical presentation is complex, with a common feature of susceptibility to exacerbations leading to loss of disease control, hospitalizations, and in some cases, progressive loss of lung function^2^. Exacerbations of asthma are most often caused by common respiratory viruses^3,4^, with rhinoviruses (RV) responsible for up to 80% of asthma attacks^3^. RVs that have been initially considered as benign viruses, now are also linked to the early-life development of asthma, severe bronchiolitis in infants and fatal pneumonia in elderly and immunocompromised patients^5,6^. Likewise, human coronaviruses have not been strongly linked with asthma pathology^7^. However, the current pandemic of severe acute respiratory syndrome coronavirus 2 (SARS-CoV-2) has been challenging this view, resulting in a range of contradictory observations of asthma being considered a risk factor for SARS-CoV-2 infection and coronavirus disease 2019 (COVID-19) severity^8–12^, not being a risk factor or even constituting a protection from the disease^13–16^, depending on its phenotype, severity, and treatment. Another important factor for asthma development and exacerbations is exposure to inhaled allergens. House dust mite (HDM) is the major source of perennial allergens worldwide. HDM sensitization is found in around 50%–85% of patients with asthma, and HDM exposure correlates with asthma severity^17^. There are strong epidemiological links between RV infections, allergen exposure and sensitization on the risk of asthma development and the rates of exacerbations^6,18^. Children with early life RV-induced wheezing and aeroallergen sensitization have a high incidence of asthma in later years^6^. Combination of virus detection in the airways with the high allergen exposure markedly increases the risk of hospital admission^19^. In line with this, HDM immunotherapy significantly reduces risk of asthma exacerbations^20^. It has been also recently suggested that allergen exposure might influence SARS-CoV-2 infection patterns in the general population^21,22^. However, the underlying mechanisms of these noxious, reciprocal allergen-virus effects in asthma pathogenesis are incompletely understood.

The host response to the RV infection encompasses its RNA recognition by the endosomal toll-like receptor 3 (TLR) 3, TLR7/8 and cytoplasmic RNA helicases: retinoic acid-inducible gene I (RIG-I) and melanoma-differentiation-associated gene 5 (MDA5)^23^, whereas its capsid might interact with the cell surface TLR2 and initiate myeloid differentiation primary response 88 (MyD88)-dependent nuclear factor ‘kappa-light-chain-enhancer’ of activated B cells (NF-kB) activation^24^. RIG-I, in its monomeric form, binds to the 5’ end of viral RNA, undergoes conformational changes, and interacts with mitochondrial antiviral signaling protein (MAVS)^25^. MAVS recruits tumor necrosis factor receptor-associated factor 3 (TRAF3) to activate TRAF family member-associated nuclear factor kappa B activator (TANK)-binding kinase (TBK)−1 and IκB kinase ε (IKKε) complex. TBK1 complex mediates phosphorylation of interferon (IFN) response factors and subsequent induction of type I and type III (I/III) IFNs^25^. Interferons further signal via their respective receptors, which leads to the broad expression of interferon-stimulated genes (ISGs)^25^. Epithelial antiviral response is sufficient to clear RV infection in healthy airways^26^. In asthma, however, we and others demonstrated several alternations in RV-induced type I/III IFN responses^27–29^, but the pathomechanisms of those changes still remain elusive, suggesting greater complexity than previously anticipated, and potential involvement of additional factors such as allergens and other viruses adding to this complexity. SARS-CoV-2 is also sensed by RIG-I and MDA5, but due to several evasion mechanisms, induction of IFNs by SARS-CoV-2 is reduced or delayed^30,31^. There is still limited understanding of epithelial response to SARS-CoV-2 in asthma or in the presence of underlying allergic inflammation or other viral infection.

Other important host factors involved in sensing viruses, bacteria and other noxious agents are inflammasomes. Inflammasomes are supramolecular complexes, composed of a sensor protein, such as NLR Family Pyrin Domain Containing 3 (NLRP3), RIG-I, and others, adaptor protein apoptosis-associated speck-like protein containing CARD (ASC), and caspase-1^32–34^. They are responsible for cleavage and release of the mature, active forms of IL-1β and IL-18, and induction of the proinflammatory cell death called pyroptosis^32^. Activation of RIG-I and NLRP3 inflammasomes has been demonstrated in macrophages and dendritic cells after infection with some respiratory RNA viruses, including RV^35,36^, influenza A (IAV)^36–38^, SARS-CoV^39,40^ and most recently SARS-CoV-2^41,42^. However, activation of any epithelial inflammasomes by these viruses in vivo in human airways and their involvement in pathology of asthma remain poorly understood. It is not known whether they are necessary to clear infection or in contrast, whether they initiate mucosal hyperinflammation delaying virus clearance^43,44^, especially in the scenario when the same sensor protein, such as RIG-I or MDA5 can be involved in type I/III IFN response or in inflammasome activation. Likewise, an involvement of NLRP3 inflammasome in HDM-models of asthma and in severe asthma in humans has been demonstrated, however data are conflicting and remain poorly understood^45–47^. Finally, airway epithelial response in health or during the preexisting allergic inflammation in asthma and combined infection with RV and SARS-CoV-2 is unknown.

Therefore, we analyze mechanisms of airway epithelial sensing and response to RV infection using controlled experimental in vivo RV infection in healthy controls and patients with asthma and in vitro models of HDM exposure and RV/SARS-CoV-2 co-infection in primary airway epithelial cells from both groups. We show that RV infection in patients with asthma leads to overactivation of RIG-I inflammasomes which diminish RIG-I accessibility for type I/III IFN responses, leading to their functional impairment, prolonged viral clearance and unresolved inflammation in vivo and in vitro. Pre-exposure to HDM augments RIG-I inflammasome activation and additionally inhibits IFN-I/III responses. Co-infection of RV and SARS-CoV-2 augments RIG-I inflammasome activation and epithelial inflammation in patients with asthma, especially in the presence of HDM.

## Results

### Intranasal infection with rhinovirus induced inflammasome-mediated immune responses in the epithelium of lower airways in asthma

**Fig. 1:**
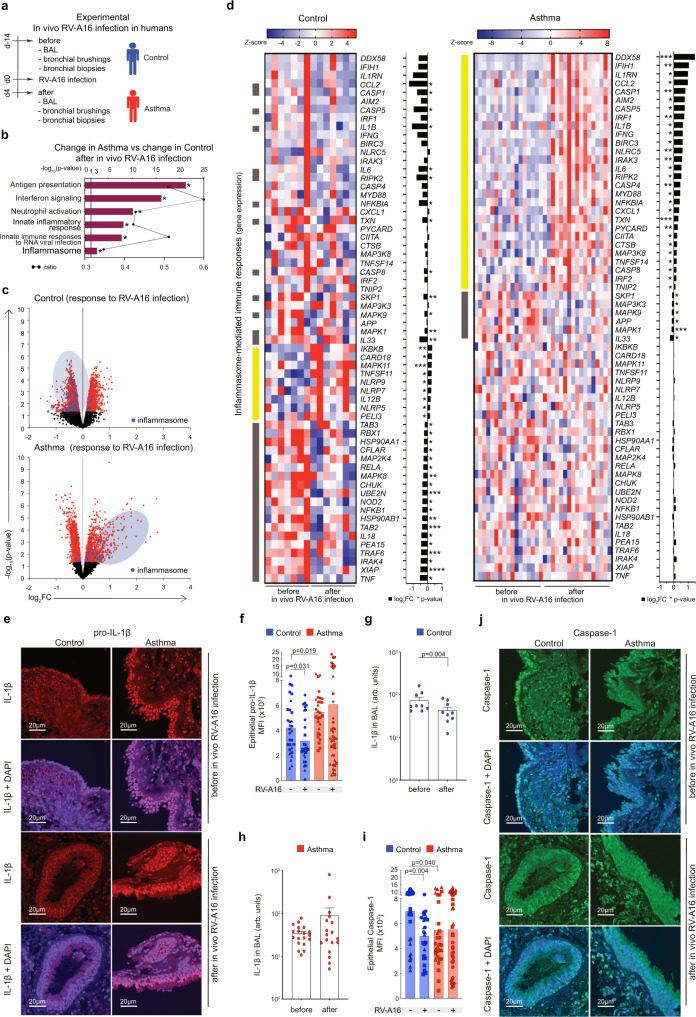
Intranasal infection with rhinovirus induced inflammasome-mediated immune responses in the epithelium of lower airways in asthma. **a** Overview of the experimental in vivo RV-A16 infection in humans. **b** Top significantly enriched pathways within genes changed after in vivo RV-A16 infection in bronchial brushings from patients with asthma compared to genes changed in control individuals (control *n* = 7, asthma *n* = 17). Black line represents a ratio of genes in the experiment over the whole pathway set. **c** Volcano plots of all (black), significant (red), and significant inflammasome-mediated immune response (blue) genes in bronchial brushings from controls (upper panel) and patients with asthma (lower panel) after in vivo RV-A16 infection (control *n* = 7, asthma *n* = 17). **d** Heatmap of genes encoding inflammasome-mediated immune responses after in vivo RV-A16 infection in controls (left panel) and patients with asthma (right panel) presented together with the corresponding log_2_ fold change (FC) expression changes (black bars) (control *n* = 7, asthma *n* = 17). Yellow and grey left-side color bars represent genes upregulated and downregulated, respectively. **e–f** Representative confocal images of pro-IL-1β in bronchial biopsies at baseline and after in vivo RV-A16 infection, scale bars: 20 μm. Quantification based on the MFI x10^3^: 10 equal epithelial areas from each biopsy (demonstrated as circles, squares, triangles, or diamonds) of control subjects (*n* = 3, before; *n* = 3, after) and patients with asthma (*n* = 3, before; *n* = 4, after). **g–h** Secretion of IL-1β to BAL fluid before and after in vivo RV-A16 infection in (**g**) controls (*n* = 9) and (**h**) patients with asthma (*n* = 19, before; *n* = 18, after). Data are presented as arbitrary units (arb. units). **i**, **j** Representative confocal images of caspase-1 in bronchial biopsies at baseline and after in vivo RV-A16 infection, scale bars: 20 μm. Quantification based on the mean fluorescence intensity (MFI) x10^3^: 10 equal epithelial areas from each biopsy (demonstrated as circles, squares, triangles, or diamonds) of control subjects (*n* = 3) and patients with asthma (*n* = 3, before; *n* = 4, after). Patients with asthma are presented in red, control individuals are presented in blue. (*n*) indicates the number of biologically independent samples examined from one in vivo RV-A16 infection. Heatmap displays normalized gene expression across the groups (row normalization). Transcriptome data analyzed with Bioconductor microarray analysis workflow [https://www.bioconductor.org/packages/release/workflows/vignettes/arrays/inst/doc/arrays.html], raw *p*-value presented. Asterisks represent statistical significance, *p*-value: *<0.05; **<0.005; ***<0.0005, ****<0.00005. Bar graph data present mean ± SEM analyzed with one-way ANOVA (Kruskal–Wallis test), RM one-way ANOVA (Friedman test), mixed-effects model with post-hoc analysis as appropriate, or paired two-tailed *T*-test or Wilcoxon test, as appropriate, depending on the data relation and distribution. Source data are provided as Source Data files. Arb. units arbitrary units; BAL Bronchoalveolar lavage, MFI mean fluorescence intensity, RV-A16 rhinovirus A16.

First, we aimed to investigate mechanisms of airway epithelial sensing in bronchial epithelium in the rhinovirus (RV)-induced asthma exacerbations in vivo in humans in the controlled, experimental settings. Therefore, we analyzed bronchial brushings, bronchial biopsies and bronchoalveolar lavage fluid (BAL) from the controlled, experimental RV infection of patients with allergic asthma and healthy controls in vivo. Samples were collected two weeks before (d-14, baseline) and 4 days after infection (Fig. 1a), as reported previously^48^. Both groups were seronegative for anti-RV antibodies prior to infection and only individuals without recent natural respiratory infection underwent the experimental infection. Within asthma group, patients with mild and moderate disease^49^, well-, partly- and poorly controlled disease^49^, eosinophilic low and high asthma^50^, and patients treated with or without inhaled corticosteroids (ICS) were equally distributed (Supplementary Table 1). In case of no-ICS group short-acting beta-agonists (SABA) were used only as required and not chronically. They also presented mostly type 2 high asthma, according to the nasal and bronchial cytokine profile^48^. An unbiased analysis of the gene ontologies and networks revealed significant differences in antigen presentation, interferon signaling and innate immune responses to RNA viral infection pathways in bronchial brushings from patients with asthma as compared to control individuals in response to RV infection (Fig. 1b). Importantly, we also noted a significant enrichment of genes in the inflammasome-mediated immune responses in asthma (Fig. 1b, Supplementary Table 2). Many of these genes have been also included in the first ranked categories (Supplementary Table 2). Once investigated in detail, we found a strong upregulation of transcriptome profiles of inflammasome-mediated immune responses in bronchial brushings from patients with asthma, in a sharp contrast to the downregulation or no change of similar genes in control individuals (Fig. 1c, d). We further validated the expression of IL-1β and caspase-1 proteins in bronchial biopsies of the same patients. We found higher expression of IL-1β in the epithelial areas of the bronchial biopsies of patients with asthma at baseline as compared to healthy controls (Fig. 1e, f). In line with its gene expression in bronchial brushings, we noted a decrease in IL-1β protein expression following RV infection in healthy controls, in contrast to the permanent upregulation of IL-1β in patients with asthma (Fig. 1e, f). Additionally, we assessed concentrations of mature IL-1β protein secreted into the BAL. In agreement with the epithelial mRNA and protein expression of IL-1β, we found significantly decreased IL-1β protein concentration in BAL fluid from control individuals 4 days after infection, whereas in patients with asthma IL-1β protein concentrations in BAL fluid tended to be increased, though this increase was not statistically significant (Fig. 1g, h). Likewise, we also noted downregulation of epithelial expression of caspase-1 in bronchial biopsies from healthy controls 4 days after infection, whereas it did not change in patients with asthma (Fig. 1i, j). Finally, to elucidate if treatment with ICS would impact any of the observed responses in patients with asthma, we stratified them according to their baseline treatment (Supplementary Fig. 1). We have not found any major differences in expression of RIG-I inflammasome-related genes or IL-1β concentration in BAL between groups treated with or without ICS before and after rhinovirus infection (Supplementary Fig. 1a–e). Altogether, we demonstrated here that inflammasome is activated and there is an upregulation of inflammasome-mediated immune responses in bronchial epithelium of patients with asthma after in vivo infection with RV, whereas it is either being actively suppressed or already resolved 4 days after infection in healthy controls.

### Augmented rhinovirus-induced RIG-I but not NLRP3 inflammasome activation in bronchial epithelium in asthma

**Fig. 2:**
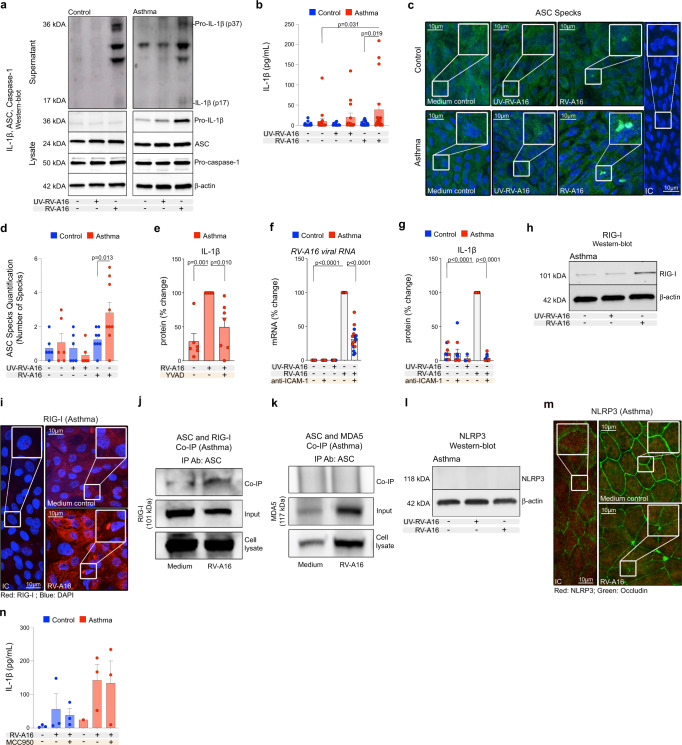
Augmented rhinovirus-induced RIG-I, but not NLRP3 inflammasome activation in bronchial epithelium in asthma. **a** Representative Western Blot images of secreted IL-1β (apical compartment), and pro-IL-1β, ASC, pro-caspase-1 and β-actin (cell lysates) in in vitro-cultured HBECs from control subjects (*n* = 3, left panel) and patients with asthma (*n* = 3, right panel). **b** IL-1β release to the apical compartment assessed by ELISA in in vitro-cultured HBECs from control (*n* = 22, vehicle; *n* = 14, UV-RV-A16; *n* = 23, RV-A16) and asthma (*n* = 17, vehicle; *n* = 14, UV-RV-A16; *n* = 18, RV-A16). **c** Representative confocal images of ASC speck formation in in vitro-cultured HBECs from control individuals and patients with asthma (control *n* = 3, asthma *n* = 3); scale bars: 10 μm. **d** Quantification of ASC specks, presented as a number of specks (mean from 5–11 equal epithelial areas from two/three technical replicates from control *n* = 3, asthma *n* = 3). **e** IL-1β release to the apical compartment assessed by ELISA (*n* = 6, HDM; *n* = 7, HDM + RV-A16, HDM + RV-A16 + YVAD) in in vitro-cultured HBECs from patients with asthma in the presence or absence of caspase-1 inhibitor (YVAD). **f** Expression of *RV-A16 positive strand* (RV-A16 viral RNA) in in vitro-cultured HBECs from patients with asthma and controls (*n* = 16, vehicle, ICAM-1, RV-A16, RV-A16 + ICAM-1; *n* = 8, UV-RV-A16) after anti-ICAM-1 treatment was assessed using RT-PCR and presented as a relative quantification (RQ = 2^-ΔΔCt^) as compared to the vehicle condition. **g** IL-1β release to the apical supernatants assessed by ELISA in in vitro–cultured HBECs from patients with asthma and healthy controls (*n* = 10, vehicle, ICAM-1, RV-A16, RV-A16 + ICAM-1; *n* = 3, UV-RV-A16) in the presence or absence of anti-ICAM-1 combined with RV-A16 infection. **e-g** Data are presented as the percentage of the response after in vitro RV-A16 treatment. **h** Representative Western Blot images of RIG-I protein expression in in vitro-cultured HBECs from patients with asthma (*n* = 4). **i** Representative confocal images of RIG-I in in vitro-cultured HBECs from patients with asthma (*n* = 3); scale bars: 10 μm. **j** Co-immunoprecipitation (co-IP) of ASC/RIG-I complex using anti-ASC antibodies followed by RIG-I detection in the presence of HDM in in vitro-cultured HBECs from patients with asthma (*n* = 4). **k** Co-immunoprecipitation (co-IP) of ASC/MDA5 complex using anti-ASC antibodies followed by MDA5 detection in in vitro-cultured HBECs from patients with asthma (*n* = 3). **l** Representative Western Blot images of NLRP3 protein in in vitro-cultured HBECs from patients with asthma (*n* = 4). **m** Representative confocal images of NLRP3 and Occludin in vitro-cultured HBECs from patients with asthma in the presence of HDM (*n* = 3), scale bars: 10 μm. **n** IL-1β release to the apical compartment in in-vitro-cultured HBECs with/without RV-A16 and NLRP3 inflammasome inhibitor (MCC950) (control *n* = 3, asthma *n* = 3). HBECs from patients with asthma are presented in red, HBECs from control individuals are presented in blue. (*n*) indicates the number of biologically independent samples examined over at least three independent experiments. Bar graph data show mean ± SEM analyzed with one-way ANOVA (Kruskal–Wallis test), RM one-way ANOVA (Friedman test) or mixed-effects model with post-hoc analysis, as appropriate, depending on the data relation (paired or unpaired) and distribution. Source data are provided as Source Data files. *anti-ICAM-1*, anti-ICAM-1 antibody; Co-IP Co-immunoprecipitation, HBECs human bronchial epithelial cells, HDM house dust mite, IC Isotype control, IP Ab antibodies used for co-precipitation, *MCC950*, NLRP3 inflammasome inhibitor; RV-A16 rhinovirus A16; UV-RV-A16 UV-treated rhinovirus A16; YVAD YVAD- (caspase-1 inhibitor).

Having demonstrated rhinovirus-induced inflammasome activation in patients with asthma in vivo, we aimed to further characterize mechanisms, sensors and timelines of this phenomenon. First, we analyzed publicly available next-generation sequencing (NGS) data of in vitro RV-infected differentiated primary human bronchial epithelial cells (HBECs) from healthy controls and patients with asthma 24 h after infection^51^. Confirming our in vivo results, an unbiased analysis of pathways and ontologies, revealed upregulated interferon signaling and innate immune responses to RNA viral infections (Supplementary Fig. 2a, Supplementary Table 3). We also noted significant enrichment of inflammasome-mediated immune responses, in this early time point happening in control and asthma samples, yet still ranking slightly higher in patients with asthma (Supplementary Fig. 2a). Accordingly, epithelium from both patients with asthma and control individuals showed increased inflammasome-mediated immune responses after RV infection (Supplementary Fig. 2b, c), but the inflammasome-related molecules such as *CASP1* (caspase-1), *IL6*, NLR family CARD domain containing 5 (*NLRC5)*, *CXCL1* and others were significantly more upregulated in asthma (Supplementary Fig. 2d). Next, we investigated mechanisms of the release of mature IL-1β in HBECs upon RV infection in vitro in a dose and time-dependent manner (Fig. 2a–c, Supplementary Fig. 3a–c). RV infection at a multiplicity of infection (MOI) 0.1, but not UV-inactivated RV (UV-RV), induced secretion of mature IL-1β 24 h after infection (Fig. 2a, b, Supplementary Fig. 3b, c), which was significantly increased in patients with asthma (Fig. 2b). Inflammasome activation was accompanied by a higher virus replication at this time point (Supplementary Fig. 3d). We also demonstrated that mature IL-1β release was paired with formation of ASC specks in HBECs from control individuals and patients with asthma infected with RV (Fig. 2c). Again, complementary to IL-1β secretion, ASC specks count was higher in patients with asthma (Fig. 2d). We did not observe ASC-speck formation after UV-RV alone. Once we demonstrated that RV-induced inflammasome activation is augmented in patients with asthma, we also looked at the baseline status of IL-1β expression and the influence of infection on the inflammasome priming step in both groups. We observed higher expression of pro-IL-1β protein in HBECs from patients with asthma at baseline in vitro (Fig. 2a, Supplementary Fig. 3e), in bronchial biopsies in vivo (Fig. 1e, f), and a trend of moderate upregulation of IL-1β concentration in bronchoalveolar lavage (BAL) fluid in vivo (Supplementary Fig. 3f). RV and UV-RV stimulation further increased expression of pro-IL-1β mRNA and protein, especially in epithelium in asthma (Fig. 2a, Supplementary Fig. 3e, g). RV infection did not affect protein expression of ASC or pro-caspase-1 (Fig. 2a and Supplementary Fig. 3h, i). Next, we noted significant inhibition of RV-induced inflammasome activation upon caspase-1 inhibitor (YVAD) treatment (Fig. 2e), while, as expected, it did not affect inflammasome priming (Supplementary Fig. 3j). To investigate whether active RV infection is necessary for inflammasome activation in HBECs, we blocked RV entry to the cells using monoclonal antibodies blocking its receptor ICAM-1. Indeed, blocking RV entry significantly diminished RV infection (Fig. 2f) and completely inhibited RV induction of mature IL-1β secretion, to levels comparable to UV-RV-A16 treatment (Fig. 2g). Notably, anti-ICAM-1 antibody combined with RV-A16 infection, but not anti-ICAM-1 antibody alone, decreased expression of RV-induced *ICAM-1*, and other antiviral and inflammasome related molecules, namely *IFNB* (IFN-β), *IFNL* (IFN-λ), *IFIH1* (MDA5), *DDX58* (RIG-I), *IL1B* (IL-1β), *CASP1* (caspase-1) and *GSDMD* (gasdermin D) (Supplementary Fig. 3k). Using targeted proteomics, we further noted that in addition to IL-1β, also IL-18, IL-1α, tumor necrosis factor (TNF) and TNF-related activation-induced cytokine (TRANCE) were released 24 h after RV infection in HBECs from patients with asthma (Supplementary Fig. 3l) indicating, that RV-induced epithelial inflammasome activation participated in the heightened proinflammatory responses at the bronchial barrier sites in asthma. Additionally, RV infection increased mRNA expression and protein secretion of thymic stromal lymphopoietin (TSLP) from epithelium of controls and patients with asthma (Supplementary Fig. 3m). Finally, we investigated which of the pattern recognition receptors (PRRs) expressed in human bronchial epithelium acts as a sensor and activator of inflammasome assembly. Based on the abundant expression after RV infection in vivo and in vitro (Fig. 1d, Supplementary Fig. 2b, c), and their capability to form inflammasomes in response to other RNA viruses in hematopoietic cells^34,35^ or in epithelium^36^, we focused on RIG-I (*DDX58*) and MDA5 (*IFIH1*) receptors. *DDX58* (RIG-I) and *IFIH1* (MDA5) mRNA was expressed in HBECs from both controls and patients with asthma at baseline (Supplementary Fig. 4a, b). We observed further increases in *DDX58* (RIG-I) (Supplementary Fig. 4c, d) and *IFIH1* (MDA5) mRNA (Supplementary Fig. 4e, f) expression in HBECs upon RV infection. Interestingly, RV-induced upregulation of *DDX58* (RIG-I) was more enhanced in asthma as compared to control (Supplementary Fig. 4d). Additionally, we observed increased expression of RIG-I protein (Fig. 2h, Supplementary Fig. 4g, h) upon RV infection, accompanied by formation of RIG-I speck-like structures (Fig. 2i). Indeed, coprecipitation of ASC with RIG-I confirmed RIG-I binding to ASC upon RV infection (Fig. 2j). Notably, MDA5 was not bound to ASC upon RV infection (Fig. 2k). Lastly, taking into account various reports regarding NLRP3 inflammasome activation in airway epithelium upon viral infections^35,36,52,53^, we also assessed its importance in human bronchial epithelium. We found low expression of *NLRP3* mRNA in fully differentiated, mature bronchial epithelial cells from patients with asthma and control individuals at baseline (Supplementary Fig. 4i) or after RV infection (Supplementary Fig. 4j). In line with that, we did not detect the expression of NLRP3 protein in differentiated HBECs from controls and patients with asthma at baseline or after RV infection (Fig. 2l, m, Supplementary Fig. 4k, l). Finally, a specific NLRP3 inflammasome inhibitor (MCC950) did not affect the secretion of mature IL-1β upon RV infection (Fig. 2n). In summary, we demonstrated here that RV infection led to activation of RIG-I inflammasome in the differentiated primary human bronchial epithelial cells, which was augmented in patients with asthma. NLRP3 and MDA5 inflammasomes were not activated by RV infection.

### Activation of the RIG-I inflammasome impaired RIG-I dependent interferon signaling in bronchial epithelium of patients with asthma

**Fig. 3:**
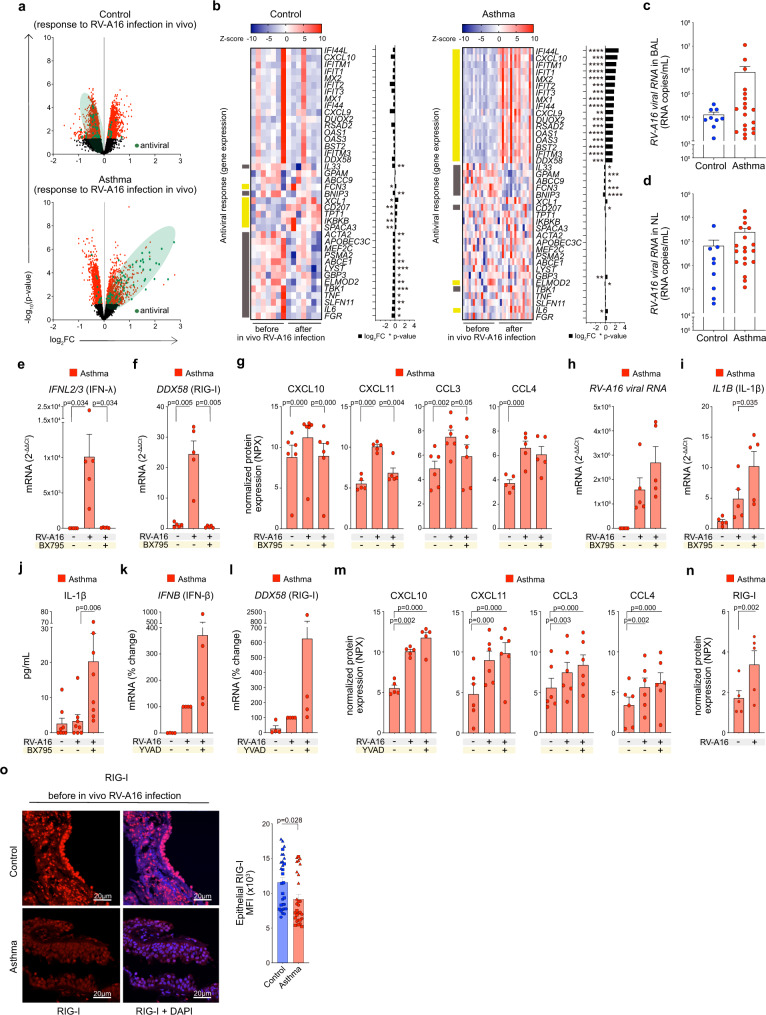
Activation of the RIG-I inflammasome impaired RIG-I-dependent interferon signaling in bronchial epithelium of patients with asthma. **a** Volcano plots of all (black), significant (red), and significant antiviral (green) genes in bronchial brushings from controls (upper panel) and patients with asthma (lower panel) after in vivo RV-A16 infection (control *n* = 7, asthma *n* = 17). **b** Heatmap of antiviral genes significantly changed four days after in vivo RV-A16 infection in healthy controls (left panel) and/or in patients with asthma (right panel) presented together with the log_2_ fold change (FC) (black bars) (control *n* = 7, asthma *n* = 17). Yellow and grey left-side color bars represent genes upregulated and downregulated, respectively. **c**, **d** RV-A16 virus load in (**c**) the bronchoalveolar lavage (BAL) fluid and **d** the nasal lavage (NL) fluid in control individuals and patients with asthma four days after in vivo RV-A16 infection (control *n* = 9, asthma *n* = 19). Data presented as viral RNA copies per 1 mL of BAL/NL. **e–j** in vitro-cultured HBECs from patients with asthma were infected in vitro with RV-A16 in the presence or absence of BX795, a chemical inhibitor of TBK1 and IKKε, or vehicle. mRNA expression of (**e**) *IFNL2/3* (IFNλ) and (**f**) *DDX58* (RIG-I) assessed using RT-PCR and presented as relative quantification (RQ = 2^-ΔΔCt^) as compared to the vehicle condition (*n* = 5). **g** Secretion of CXCL10, CXCL11, CCL3, and CCL4 proteins into the apical compartment assessed with the Proximity Extension Assay (PEA) targeted proteomics (*n* = 6). Expression of (**h**) *RV-A16 positive strand* (RV-A16 viral RNA) and (**i**) *IL1B* (IL-1β) assessed using RT-PCR and presented as relative quantification (RQ = 2^-ΔΔCt^) (*n* = 5). **j** IL-1β release to the apical compartment of in vitro-cultured HBECs from patients with asthma assessed by ELISA (*n* = 8). **k–n** in vitro-cultured HBECs from patients with asthma were infected in vitro with RV-A16 in the presence or absence of YVAD, a caspase-1 inhibitor or vehicle. mRNA expression of (**k**) *IFNB* (IFNβ) and (**l**) *DDX58* (RIG-I) (*n* = 4). Data are demonstrated as the percentage of the expression normalized to the in vitro RV-A16 condition. **m** Secretion of CXCL10, CXCL11, CCL3, and CCL4 into apical compartment assessed with the PEA proteomics (*n* = 6). **n** RIG-I release to the apical compartment assessed by PEA in in vitro-cultured HBECs from patients with asthma (*n* = 5). **o** Representative confocal images of RIG-I expression in bronchial biopsies at baseline, scale bars: 20 μm. Quantification based on the mean fluorescence intensity (MFI) x10^3^: 10 equal epithelial areas from each biopsy (demonstrated as circles, squares, triangles, or diamonds) of control subjects (*n* = 3) and patients with asthma (*n* = 3). Fig. 3a–d, (**o**) presents in vivo RV-A16 infection study, Fig. 3e–n shows in vitro ALI-differentiated HBECs. Patients with asthma/HBECs from patients with asthma are presented in red, control individuals/HBECs from control individuals are presented in blue. (*n*) indicates the number of biologically independent samples examined over one infection (in vivo RV-A16 infection) or at least three independent experiments (in vitro-cultured HBECs). Heatmap displays normalized gene expression across the groups (row normalization). Transcriptome data analyzed with Bioconductor microarray analysis workflow [https://www.bioconductor.org/packages/release/workflows/vignettes/arrays/inst/doc/arrays.html], raw p-value presented. Asterisks represent statistical significance, p-value: *<0.05; **<0.005; ***<0.0005, ****<0.00005. Bar graph data present mean ± SEM analyzed with one-way ANOVA (Kruskal–Wallis test), RM one-way ANOVA (Friedman test), or mixed-effects model with post-hoc analysis, as appropriate, depending on the data relation and distribution. Proximity Extension Assay (PEA) targeted proteomics data were analyzed by Bioconductor limma package^104^, raw *p*-value presented, and presented as normalized protein expression (NPX). Source data are provided as Source Data files. ALI Air-liquid interface, BAL bronchoalveolar lavage; *BX795*, TBK1/IKKε inhibitor; HBECs differentiated human bronchial epithelial cells, MFI mean fluorescent intensity*,* NL nasal lavage, NPX normalized protein expression, RV-A16 rhinovirus A16, YVAD ac-YVAD-cmk (caspase-1 inhibitor).

Since the major function of RIG-I is recognition of RNA viruses^25^, we also analyzed the status of antiviral genes and proteins involved in in vivo responses to RV infection. In line with inflammasome-mediated immune responses, the majority of genes encoding antiviral pathways were still upregulated 4 days after in vivo RV infection in patients with asthma while they were either downregulated or not changed in healthy controls at the same timepoint after RV infection (Fig. 3a, b, Supplementary Table 4). These data suggest less effective resolution of RV infection and delayed clearance of the virus in asthma^54^. Indeed, RV load in the bronchoalveolar lavage fluid in asthma was around 100-fold higher than in controls and the peak nasal lavage virus load was 25-fold higher in patients with asthma than in healthy controls, though this difference was not statistically significant (Fig. 3c, d). Thus, we showed here that bronchial epithelium from healthy individuals can efficiently respond to RV infection which leads to rapid virus clearance and subsequent resolution of antiviral responses. In contrast, in asthma, the lack of resolution of antiviral responses and delayed virus clearance suggest that there is an ongoing process in epithelium, which impairs the effectiveness of antiviral mechanisms. Therefore, we hypothesized that excessive RIG-I inflammasome activation and subsequent IL-1β secretion in response to RV infection in asthma, resulted in persistent, but less efficient RIG-I-mediated anti-RV response. Hence, we further studied whether RIG-I activation of MAVS/TBK1/IKKε and downstream interferon signaling inhibits RIG-I inflammasome activation and conversely if formation of RIG-I inflammasome inhibits interferon signaling in vitro. First, we used BX795, a chemical inhibitor of TBK1 and IKKε. As expected, it blocked expression of *IFNL2/3* (IFN-λ) in HBECs of patients with asthma (Fig. 3e) and subsequently decreased expression of *DDX58* (RIG-I) (Fig. 3f). BX795 treatment also reduced expression of interferon-stimulated chemokines: CXCL10, CXCL11 and CCL3 (Fig. 3g). It also led to a trend to increased RV infection (Fig. 3h), as well as to significantly augmented inflammasome priming (Fig. 3i) and activation (Fig. 3j). Next, we blocked IL-1β processing by RIG-I inflammasome with the use of the caspase-1 inhibitor YVAD and investigated interferon signaling and RV infection. Inhibition of RIG-I inflammasome activation and subsequent IL-1β signaling indeed led to trends of increasing expression of *IFNB* (IFN-β) (Fig. 3k) and *DDX58* (RIG-I) mRNA (Fig. 3l) and production of CXCL10, CXCL11, CCL3, and CCL4 (Fig. 3m). Since ASC specks and assembled inflammasome complexes are often released from the cells together with mature IL-1β^55^, we analyzed RIG-I protein expression also in the supernatants of the cells. Accordingly, we found that activation of RIG-I inflammasome led to increased release of RIG-I protein from the epithelial cells (Fig. 3n), which may translate to the decreased expression of RIG-I protein assessed in bronchial biopsies in vivo (Fig. 3o). In summary, these data suggest that increased RV-dependent RIG-I inflammasome activation in bronchial epithelium disturbed the effectiveness of RIG-I dependent anti-RV responses in asthma.

### House dust mite enhanced rhinovirus-induced inflammasome activation in bronchial epithelium in asthma

**Fig. 4:**
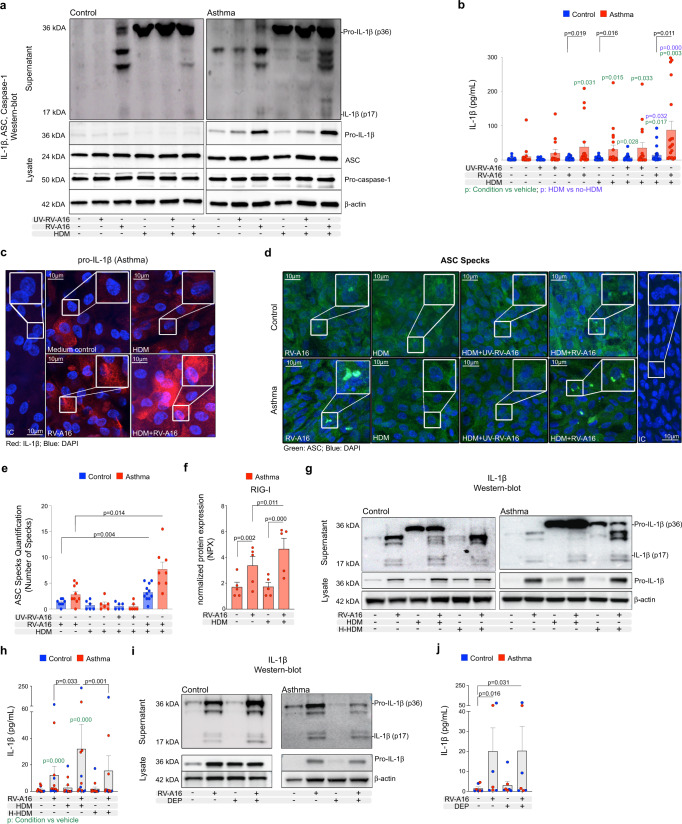
House dust mite enhanced rhinovirus-induced inflammasome activation in bronchial epithelium in asthma. **a** Representative Western Blot images of secreted IL-1β (apical compartment), and pro-IL-1β, ASC, pro-caspase-1 and β-actin (cell lysates) in in vitro-cultured HBECs from control subjects (*n* = 3, left panel) and patients with asthma (*n* = 3, right panel). **b** IL-1β release to the apical compartment in in vitro-cultred HBECs from controls (*n* = 22, vehicle, HDM; *n* = 14 UV-RV-A16, HDM + UV-RV-A16; *n* = 23, RV-A16, HDM + RV-A16; *n* = 21, HDM + RV-A16) and patients asthma (*n* = 17, vehicle; *n* = 14, UV-RV-A16; *n* = 16, HDM + UV-RV-A16; *n* = 18, RV-A16; *n* = 19, HDM, HDM + RV-A16) assessed by ELISA. **c** Representative confocal images of IL-1β in in vitro-cultured HBECs from patients with asthma (*n* = 3); scale bars: 10 μm. **d** Representative confocal images of ASC speck formation in in vitro-cultured HBECs from control individuals (*n* = 3) and patients with asthma (*n* = 3); scale bars: 10 μm. **e** Quantification of ASC specks, presented as a number of specks (mean from 5–11 equal epithelial areas from two technical replicates from control *n* = 3, asthma *n* = 3). **f** RIG-I release to the apical compartment assessed by the PEA in in vitro-cultured HBECs from patients with asthma (*n* = 5). Data are presented as normalized protein expression (NPX). **g** Representative Western Blot images of secreted IL-1β (apical compartment), and pro-IL-1β and β-actin (cell lysates) in in vitro-cultured HBECs from control subjects (*n* = 3, left panel) and patients with asthma (*n* = 3, right panel). **h** IL-1β release to the apical compartment assessed by ELISA in in vitro-cultured HBECs from patients with asthma (*n* = 7) and healthy controls (*n* = 6) in indicated conditions. **i** Representative Western Blot images of secreted IL-1β (apical compartment), and pro-IL-1β and β-actin (cell lysates) in in vitro-cultured HBECs from control subjects (*n* = 3, left panel) and patients with asthma (*n* = 3, right panel) after DEP + RV-A16 treatment. **j** IL-1β release to the apical compartment assessed by ELISA in in vitro-cultured HBECs from patients with asthma (*n* = 3) and healthy controls (*n* = 4). HBECs from patients with asthma are presented in red, HBECs from control individuals are presented in blue. (*n*) indicates the number of biologically independent samples examined over at least three independent experiments. Bar graph data show mean ± SEM analyzed with one-way ANOVA (Kruskal–Wallis test), RM one-way ANOVA (Friedman test) or mixed-effects model with post-hoc analysis, as appropriate, depending on the data relation (paired or unpaired) and distribution. Green *p*-values demonstrate differences between marked condition and vehicle. Purple *p*-values demonstrate differences between HDM stimulation vs the same condition without HDM. PEA data were analyzed by Bioconductor limma package^104^, raw *p*-value presented. Vehicle conditions from the same experiments presented on Fig. 2. Source data are provided as Source Data files. DEP diesel exhaust particles, HBECs human bronchial epithelial cells, HDM house dust mite, H-HDM heat-inactivated HDM, IC Isotype control, NPX normalized protein expression, RV-A16 rhinovirus A16, UV-RV-A16 UV-treated rhinovirus A16.

Knowing that house dust mite (HDM) exposure combined with rhinovirus infection have an especially detrimental impact on severity of asthma exacerbations in children and adults^6,19^, we investigated the effect of HDM on rhinovirus-induced RIG-I inflammasome activation in bronchial epithelium in a dose, time and source-dependent manner in vitro (Supplementary Fig. 5a–c). HDM stimulation combined with RV infection, significantly increased mature IL-1β secretion in bronchial epithelium of controls and patients with asthma, yet this effect was much more pronounced in asthma (Fig. 4a, b). HDM alone did not lead to release of inflammasome-processed mature IL-1β, but it induced the abundant release of non-mature pro-IL-1β in both groups (Fig. 4a). HDM pre-exposure followed by RV infection further increased expression of pro-IL-1β protein, especially in epithelium in asthma (Fig. 4a, c, Supplementary Fig. 5d), suggesting combined effects of HDM and RV-replication independent and dependent mechanisms on pro-IL-1β expression. Expression of ASC and pro-caspase-1 proteins was stable and comparable between controls and patients with asthma (Fig. 4a, Supplementary Fig. 5e, f). HDM prestimulation also increased RV-induced ASC specks formation in HBECs from controls and patients with asthma (Fig. 4d, e), confirming HDM involvement in the enhancement of inflammasome activation. In line with the lack of the release of processed IL-1β, we did not observe formation of ASC-specks after HDM stimulation alone (Fig. 4d, e). Additionally, HDM pre-exposure increased RV-induced release of RIG-I from the epithelial cells of patients with asthma (Fig. 4f). To understand if HDM effects on RV-induced RIG-I inflammasome activation depends on the protein content of the HDM extract, we treated HBECs with HDM and heat-inactivated HDM (H-HDM). Notably, heat inactivation of HDM revoked HDM-enhanced RV-induced secretion of mature IL-1β in patients with asthma and healthy controls (Fig. 4g, h). Additionally, it decreased the release of pro-IL-1β from the cells of both groups (supernatant) (Fig. 4g). Thus, our data demonstrate that increased secretion of RV-dependent mature IL-1β, as well as increased release of RV-independent pro-IL-1β from the cells upon HDM stimulation, depends on the protein content of the HDM extract. Next, we aimed to understand if the observed HDM-mediated increase of RV-induced RIG-I inflammasome activation is exclusive to HDM, or if a similar effect can be elicited by other stimuli, also reported as exacerbating factors in asthma, such as Alternaria alternata (A. alternata)^56^ or diesel exhaust particles (DEP)^57^. We observed similar pattern of changes in expression of inflammasome-related and antiviral genes in patients with asthma sensitized to HDM or other allergens after experimental rhinovirus infection in vivo (Supplementary Fig. 5g). Similarly, we indeed observed that prestimulation of bronchial epithelium with A. alternata significantly increased RV-induced mature IL-1β secretion in controls and patients with asthma (Supplementary Fig. 5h) to the same level. Interestingly, however, in contrast to HDM extract, A. alternata alone was able to induce a potent release of mature IL-1β only in asthma, but not in healthy individuals (Supplementary Fig. 5h). Finally, DEP stimulation alone increased pro-IL-1β priming (Fig. 4i) in controls and in asthma, but it did not have any effect on RV-induced RIG-I inflammasome activation in the bronchial epithelium of any group (Fig. 4i, j).

In summary, we demonstrated here, that while HDM alone did not activate any epithelial inflammasome in human bronchial epithelium, it led to the release of pro-IL-1β. Additionally, HDM pre-exposure increased RV-induced RIG-I inflammasome activation in bronchial epithelium, which was augmented in patients with asthma and depended on the protein content of HDM. A. alternata also enhanced RV-induced mature IL-1β release in control subjects, whereas in asthma, it potently induced release of mature IL-1β even without RV infection, suggesting a different mechanism of action. We did not observe any crosstalk between DEP-induced effects and epithelial RV-induced RIG-I inflammasome.

### House dust mite impaired interferon responses in rhinovirus-infected bronchial epithelium of patients with asthma

**Fig. 5:**
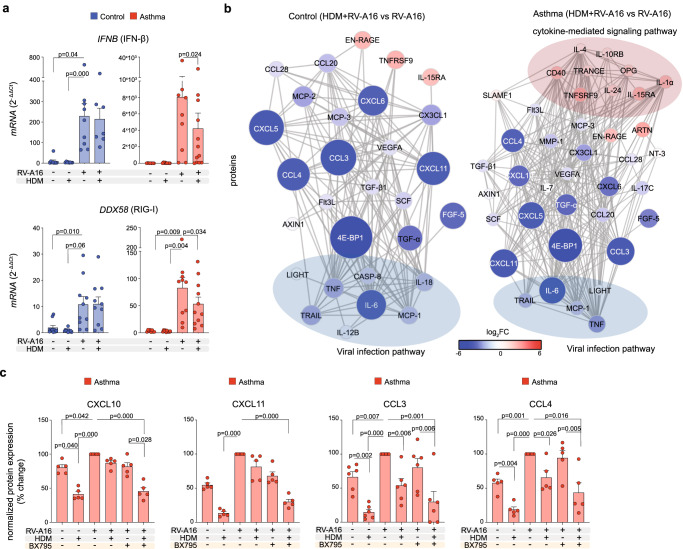
House dust mite impaired interferon responses in rhinovirus-infected bronchial epithelium of patients with asthma. **a** mRNA expression of *IFNB* (IFN-β) (upper panel) in in vitro-cultured HBECs from controls (*n* = 9, vehicle, RV-A16; *n* = 8, HDM; *n* = 7, HDM + RV-A16) and asthma (*n* = 7, vehicle; *n* = 9, HDM, RV-A16; *n* = 12, HDM + RV-A16), and *DDX58* (RIG-I) (lower panel) in in vitro-cultured HBECs from controls (*n* = 10) and asthma (*n* = 8, vehicle; *n* = 9, HDM, RV-A16; *n* = 11, HDM + RV-A16) assessed using RT-PCR and presented as relative quantification (RQ = 2^-ΔΔCt^) as compared to the vehicle condition. **b** Visualization of interaction network of significant proteins secreted to the apical compartment in in vitro-cultured HBECs from control individuals (*n* = 5, left panel) and patients with asthma (*n* = 8, right panel) after in vitro treatment with HDM + RV-A16, when compared to RV-A16 infection alone assessed with PEA targeted proteomics. Network nodes represent log_2_FC of significantly upregulated (red), and downregulated (blue) proteins; proteins not interacting with each other are not shown. Edges represent protein-protein interactions. Proteins enriched in viral infection or cytokine-mediated signaling pathway are marked with blue and red eclipses, respectively. **c** Expression of CXCL10, CXCL11, CCL3, and CCL4 in the apical compartment of in vitro-cultured HBECs from patients with asthma pre-treated with HDM or vehicle, followed by the in vitro infection with RV-A16 in the presence of BX795 or vehicle and assessed with PEA proteomics (*n* = 5). Data presented as the percentage of the response to the RV-A16 condition. HBECs from patients with asthma are presented in red, HBECs from control individuals are presented in blue. (*n*) indicates the number of biologically independent samples examined over at least three independent experiments. Bar graph data present mean ± SEM analyzed with one-way ANOVA (Kruskal–Wallis test), RM one-way ANOVA (Friedman test) or mixed-effects model with post-hoc analysis, as appropriate, depending on the data relation and distribution. PEA data were analyzed by Bioconductor limma package^104^, raw *p*-value presented. Source data are provided as Source Data files. *BX795*, TBK1/IKKε inhibitor; HBECs differentiated human bronchial epithelial cells, HDM house dust mite, RV-A16 rhinovirus A16.

Having demonstrated that HDM increased RV-induced RIG-I inflammasome activation, we continued to explore the effect of HDM pre-exposure on the timing and strength of antiviral responses. We found that HDM pre-treatment decreased RV-induced mRNA expression of *IFNB* (IFN-β) and *DDX58* (RIG-I) only in HBECs from patients with asthma after RV infection in vitro (Fig. 5a, Supplementary Fig. 6a, b). Notably, HDM stimulation did not influence RV infection (Supplementary Fig. 6c). When we analyzed protein expression and enriched biological pathways by targeted proteomics in the same conditions, we observed decreased cell- and IFNs- mediated antiviral responses in HBECs from control individuals and patients with asthma at 24 h post-infection (Fig. 5b). In addition, HDM in the presence of RV stimulated the release of epithelial to mesenchymal transition factors such as interleukin 15 receptor subunit alpha (IL-15RA)^58,59^, artemin (ARTN)^60^, tolerance inducing TNF receptor superfamily member 9 (TNFRSF9)^61^ (also called 4-1BB and CD137), extracellular newly identified RAGE-binding protein (EN-RAGE) alarmin^62^ in both studied groups. However, only in asthma, complementary with all our previous data, HDM simultaneously increased the activation status and release of proinflammatory and pro-remodeling proteins, such as IL-1α, signaling lymphocytic activation molecule family member 1 (SLAMF1)^63^, cluster of differentiation (CD) 40^64,65^ and TRANCE (RANKL)^66,67^ (Fig. 5b, Supplementary Table 5). Importantly, HDM pre-stimulation had a slightly additive effect to the antiviral RIG-I pathway inhibitor (BX795) and further reduced BX795-decreased protein expression of the ISGs: CXCL10, CXCL11, CCL3, and CCL4 (Fig. 5c). It all suggests that pre-exposure to HDM, before RV infection decreases IFN type I response and this way it further contributes to the enhanced inflammasome-mediated impairment of antiviral responses in patients with asthma.

### Rhinovirus and SARS-CoV-2 co-infection augmented epithelial inflammation after house dust mite exposure in asthma

**Fig. 6:**
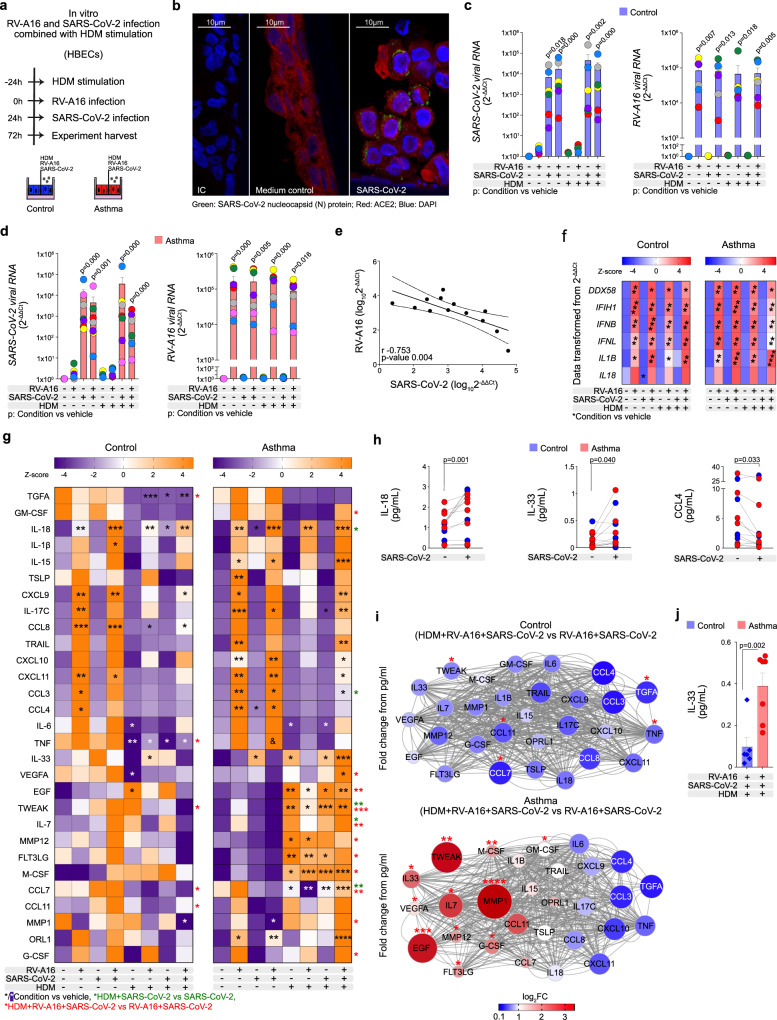
Rhinovirus and SARS-CoV-2 co-infection augmented epithelial inflammation after house dust mite exposure in asthma. **a** Overview of the in vitro RV-A16 and severe acute respiratory syndrome coronavirus 2 (SARS-CoV-2) co-infection with/without HDM pre-treatment. **b** Representative confocal images of SARS-CoV-2 nucleocapsid (N) protein and ACE2 in in vitro-cultured HBECs from patients with asthma after SARS-CoV-2 infection (*n* = 3); scale bars: 10 μm. Expression of SARS-CoV-2 virus load (average expression of *N protein*, *S protein* and *ORF1AB*) *and RV-A16 positive strand* (RV-A16 viral RNA) in in vitro-cultured HBECS from (**c**) healthy controls (*n* = 6) and **d** patients with asthma (*n* = 7) was assessed using RT-PCR and presented as relative quantification (RQ = 2^-ΔΔCt^) compared to medium condition separately for HBECs from controls and patients with asthma. **e** Correlation of log_10_-transformed RV-A16 and SARS-CoV-2 viral loads in in vitro-cultured HBECs from patients with asthma (*n* = 7) and healthy controls (*n* = 6). **f** Heatmap of gene expression in in vitro-cultured HBECs from healthy controls (*n* = 6, left panel) and patients with asthma (*n* = 7, right panel) assessed using RT-PCR and transformed from relative quantification (RQ = 2^-ΔΔCt^). **g** Heatmap of secreted proteins assessed in the apical compartments of in vitro-cultured HBECs from controls (*n* = 6, left panel) and patients with asthma (*n* = 7, right panel) after in vitro HDM pre-stimulation and RV-A16 and SARS-CoV-2 co-infection analyzed with the quantitative PEA targeted proteomics. Data are transformed from the concentrations in pg/mL. **h** IL-18, IL-33, and CCL4 secreted to the apical compartment after in vitro SARS-CoV-2 infection in in vitro-cultured HBECs from healthy controls (*n* = 6) and patients with asthma (*n* = 7) measured with the quantitative PEA. **i** Interaction network analysis of all detected proteins secreted to the apical compartment of in vitro-cultured HBECs from control individuals (*n* = 6, top panel) and patients with asthma (*n* = 7, bottom panel) after in vitro treatment with HDM + RV-A16 + SARS-CoV-2, when compared to RV-A16 + SARS-CoV-2 assessed with quantitative PEA. Network nodes represent log_2_FC of significantly upregulated (red), and downregulated (blue) proteins. Edges represent protein-protein interactions. Significantly changed proteins are indicated by asterisks (*). **j** IL-33 secreted to the apical compartment after in vitro HDM prestimulation and RV and SARS-CoV-2 coinfection in in vitro-cultured HBECs from healthy controls (*n* = 6) and patients with asthma (*n* = 7) measured with the quantitative PEA. HBECs from patients with asthma are presented in red, HBECs from control individuals are presented in blue. (*n*) indicates the number of biologically independent samples examined over two independent experiments. Black *p*-values demonstrate differences between marked conditions and vehicle. Black or white asterisks (*) represent a significant difference as compared to the vehicle from the same group. Green asterisks (*) represent a significant difference between HDM + SARS-CoV-2 vs SARS-CoV-2 conditions, red asterisks (*) represent a significant difference between HDM prestimulation combined with RV-A16 and SARS-CoV-2 coinfection compared to infection with both viruses without HDM. Bar graphs depict the mean ± SEM, whereas color-coded circles show individual data from the same donor. Data are presented as mean ± SEM analyzed with one-way ANOVA (Kruskal–Wallis test), RM one-way ANOVA (Friedman test) or mixed-effects model, with post-hoc analysis as appropriate, depending on the data relation and distribution, *p-value ≤0.05, **p-value ≤0.01, ***p-value ≤0.001, ****p-value ≤0.0001. Correlation between viral loads was calculated with Spearman’s rank correlation. Source data are provided as Source Data files. HBECs Human Bronchial Epithelial Cells, HDM House Dust Mite, IC isotype control, RV-A16 rhinovirus A16, SARS-CoV-2 Severe Acute Respiratory Syndrome Coronavirus 2.

Finally, facing the current pandemic and noting the contradictory results about asthma as a risk factor for COVID-19 in different populations^8–10,13,14^, we investigated if RV-induced RIG-I inflammasome activation and HDM-mediated decrease of IFN responses may affect SARS-CoV-2 infection. We first treated primary HBECs from healthy controls and patients with asthma with or without HDM, next after 24 h we infected them with RV, and after a further 24 h we infected them with SARS-CoV-2 for 48 h (Fig. 6a) in vitro. We confirmed infection with SARS-CoV-2 by the detection of its nucleocapsid (N) protein (Fig. 6b) and the increase of SARS-CoV-2 viral RNA (Fig. 6c, d, Supplementary Fig. 7a). In patients with asthma, we observed a trend to lower infection with SARS-CoV-2 in samples pre-infected with RV (Fig. 6d), but it did not reach statistical significance. Interestingly, however, individual samples with high RV infection (Fig. 6c, d) had lower potential to SARS-CoV-2 infection, and vice versa (Fig. 6e). This trend disappeared when SARS-CoV-2 infection preceded RV-A16 infection (Supplementary Fig. 7b–d). Next, we analyzed mRNA and protein expression of several antiviral and proinflammatory molecules. In line with our previous data, RV infection increased the expression of *DDX58* (RIG-I), *IFIH1* (MDA5), *IFNB* (IFN-β)*, IFNL1* (IFN-λ1), and *IL1B* (IL-1β) (Fig. 6f). RV infection alone also strongly induced the secretion of IFN-stimulated (CXCL10, CXCL9, CCL3, CCL4, CCL8, CXCL11) proteins and proinflammatory cytokines (IL-1β, IL-15, IL-17C, TSLP, TRAIL, IL-18, and ORL1) (Fig. 6g, Supplementary Table 6). SARS-CoV-2 alone did not induce expression of *DDX58* (RIG-I)*, IFIH1* (MDA5)*, IFNB* (IFN-β)*, IFNL1* (IFN-λ1), and *IL1B* (IL-1β) (Fig. 6f) but it increased secretion of IL-33, IL-18 and decreased CCL4 at this timepoint (Fig. 6g–h, Supplementary Table 6). Next, we aimed to investigate the effects of HDM on SARS-CoV-2 infection alone or in combination with RV-A16. We did not observe any significant effects of HDM prestimulation on replication of SARS-CoV-2 or RV (Fig. 6c, d, Supplementary Fig. 7a). HDM prestimulation increased the release of IL-18, EGF, TWEAK, and M-CSF and decreased CCL3 in SARS-CoV-2-infected HBECs from patients with asthma in comparison to SARS-CoV-2 infection alone (Fig. 6g, Supplementary Fig. 7e). Importantly, HDM pre-stimulation significantly increased the release of GM-CSF, IL-33, VEGFA, EGF, TWEAK, IL-7, MMP12, FTL3G, M-CSF, MMP1, G-CSF in double infected epithelium of patients with asthma, whereas it decreased secretion of TGFA, TNF, TWEAK, CCL7 and CCL11 in healthy controls (Fig. 6g, i). Some of these HDM-induced effects on single and double-infected cells resulted from aberrant response to HDM in asthma in terms of an increase in IL-33, RGF, TWEAK, MMP12, FTL3G, and M-CSF (Fig. 6g, i). Finally, HDM stimulation combined with RV and SARS-CoV-2 infection resulted in higher secretion of IL-33 in the epithelium of patients with asthma when compared to healthy controls (Fig. 6j).

In summary, we found here that SARS-CoV-2 infection alone did not induce *DDX58* (RIG-I)*, IFIH1* (MDA5)*, IFNB* (IFN-β)*, IFNL1* (IFN-λ1) or *IL1B* (IL-1β) at 48 h after infection, but induced IL-33, IL-18, and decreased CCL4 secretion, suggesting its early proinflammatory inflammasome-inducing and delaying antiviral capacity in asthma. In addition, especially in the presence of HDM, we observed enhanced proinflammatory responses in patients with asthma after SARS-CoV-2 co-infection with RV.

## Discussion

In the current study we demonstrated in vivo and in vitro that recognition of replicating RV by RIG-I helicase in bronchial epithelial cells of patients with asthma led to the augmented ASC recruitment, oligomerization, activation of caspase-1, processing and release of mature IL-1β via formation of RIG-I inflammasome, independently of NLRP3 or MDA5. This excessive activation of RIG-I inflammasome by RV in patients with asthma compromised RIG-I-dependent type I/III IFNs and ISG responses, leading to less effective virus clearance and to unresolved airway inflammation. In addition, we found that pre-exposure to HDM amplified RV-induced epithelial injury in patients with asthma via enhancement of pro-IL1β expression and release, additional inhibition of type I/III IFNs and activation of auxiliary pro-inflammatory and pro-remodeling proteins. Finally, we showed that prior infection with RV restricted SARS-CoV-2 replication, but co-infection augmented RIG-I inflammasome activation and epithelial inflammation in patients with asthma, especially in the presence of HDM.

There are four main inflammasomes described to date to be involved in innate antiviral immunity against RNA viruses – the NLRP3, RIG-I and in some cases MDA5 and absent in melanoma 2 (AIM2) inflammasomes^34,38,68^. They are activated by several stimuli involved in the viral infection, such as viral nucleic acids, viroporins, RNA-modulating proteins, reactive oxygen species (ROS) and others^44^. Here, we found, that in vivo in humans and in fully differentiated primary human bronchial epithelium, infection with RV, a single stranded RNA virus, leads to increased priming of pro-IL-1β in a replication independent and dependent manner, and to assembly of RIG-I/ASC inflammasome, in a replication dependent manner. We did not see involvement of NLRP3 inflammasome upon RV infection or HDM exposure or even significant expression of NLRP3 in airway epithelium at baseline in any of our in vivo, in vitro, or data mining approaches. We also did not see MDA5 forming inflammasome. Other groups observed that infection of human peripheral blood mononuclear cells, human macrophages and mouse bone-marrow derived cells with other single stranded RNA viruses, vesicular stomatitis virus (VSV) and IAV, activates RIG-I/MAVS-dependent pro-IL-1β transcription and RIG-I/ASC-dependent, but NLRP3-independent, inflammasome activation and mature IL-1β and IL-18 production^34,52^. In contrast, in undifferentiated, submerged cultures of primary human airway epithelial cells infection with IAV revealed RIG-I and NLRP3-inflammasome-dependent mature IL-1β release^36^, while infection with RV in a similar model led to activation of NLRP3/NLRC5/ASC complexes and mature IL-1β release^53^. These differences might come from (i) the undifferentiated state of the cells in the previous studies, as in non-differentiated epithelium, lacking ciliated cells, viral infection might engage different pathways than in human airways in vivo;^69,70^ (ii) from different expression of inflammasome components in undifferentiated and differentiated mature epithelium lining human airways, as we showed previously;^71^ as well as (iii) from the differences in the virus strains and serotypes^72^. Importantly, our in vitro and in vivo data consistently showed the same results that RIG-I is engaged as inflammasome in bronchial epithelium upon RV infection, which constitutes an important early time-point event, triggering subsequent airway inflammation. It is also possible, that both inflammasomes are engaged in different cellular compartments: RIG-I/ASC in airway epithelium and NLRP3 in the infiltrating inflammatory cells in the airways. Indeed, RV infection in mice leads to partly macrophage-derived, NLRP3 inflammasome-dependent airway inflammation^35^. However, neither depletion of macrophages nor NLRP3 knockout leads to the complete blockade of mature caspase-1 and IL-1β processing upon RV infection, underlining that RV induces other inflammasomes in airway bronchial epithelium^35^.

An appropriate balance between activation of RIG-I epithelial inflammasome with RIG-I-dependent type I/III IFN responses lead to the limitation of viral replication, efficient virus clearance and timely resolution of airway inflammation in case of IAV infection^36^. Likewise, we observed here that in the bronchial epithelium of healthy subjects at early time points during RV infection there was an activation of RIG-I inflammasome and inflammasome-mediated immune responses, together with efficient type I/III IFN and ISG-responses. Importantly all of these responses were actively inhibited or went back to the pre-infection state, already 4 days after in vivo infection. In contrast, in epithelium of patients with allergic asthma, there was enhanced RIG-I inflammasome activation starting early after infection and still non-resolved in vivo 4 days after infection. Overactivation of epithelial RIG-I inflammasome and subsequent increases in mature IL-1β release were at least partially responsible for the impairment of type I/III IFN/ISG responses. We demonstrated this here by blocking caspase-1 with YVAD which led to an increase in IFN-β *(IFNB)* and RIG-I (*DDX58*) together with IFN-responsive chemokines such as CXCL10, CXCL11, CCL3, and CCL4. Our findings are in line with early observations showing that IL-1β is able to attenuate transcription and translation of type I IFNs and excessive IFNα/β-induced effects^73,74^. Interestingly, we also noted here that this crosstalk between IL-1β and type I IFNs is reciprocal, as blocking phosphorylation of TBK1 and IKKε by their inhibitor BX795 and thus reducing RIG-I-induced type I interferons, significantly increased pro-IL-1β transcription and its processing by RIG-I inflammasome in airway epithelium. With this finding, we added an important point to the long-lasting discussion about the underlying origins and mechanisms of the frequent viral infections and exacerbations in patients with asthma^75,76^.

Presence of other airway barrier-damaging and/or activating factors, such as exposure to allergens in addition to the viral infection, worsens the clinical outcomes, leads to a more severe exacerbation, hospitalization or respiratory failure^7^. Mechanistically, it might be connected with the multiplication of activated pathways and/or with the additive effects of different triggers on the same pathway. HDM activates airway epithelium via, among others, TLR2/4, C-type lectins, and protease-activated receptors (PARs) in an allergen-non-specific way to initiate allergen sensitization, but also to perpetuate already developed allergic and probably non-allergic airway inflammation in the absence of the sufficiently developed inhibitory signals^46,77,78^. It was recently observed in the clinical trials that one of the strongest effects of HDM-specific immunotherapy in patients with asthma is the reduction of the rate of asthma exacerbations^20^. However, it is not well understood if and how HDM-induced signaling in the airway epithelium interferes with the effectiveness of early antiviral response. Here, we found that HDM contributes significantly to RV-induced pathologic responses in human airway epithelium in patients with asthma by (i) enhancement of non-mature pro-IL-1β expression and release, (ii) overactivation of RIG-I inflammasome and subsequent release of mature caspase-1, mature IL-1β, and RIG-I, (iii) inhibition of type I/III IFNs and ISG-responses and (iv) activation of extra proinflammatory and pro-remodeling proteins, such as IL-1α, SLAMF1^63^, CD40^64^ and TRANCE (RANKL)^67^. In the presence of both HDM and RV, we noted that increased usage of RIG-I protein engaged in inflammasome formation and its subsequent expulsion is paired with HDM-dependent inhibition of RIG-I, type I/III IFNs, and several ISGs. Notably, these effects were independent of sensitization status to HDM. At an early stage of RV infection, it might explain disturbance of the antiviral response dynamics observed in asthma by us and others^54,79^. Due to this functional reduction of RIG-I availability, the following type I/III IFN-response is less effective, not able to quickly and efficiently clear the infection and thus it is sustained up to later time points, together with the enhanced inflammasome-related proinflammatory response. Importantly, also some other airborne stimuli, such as Alternaria alternata, but not diesel exhaust particles, can contribute in its own unique way to RV-induced RIG I inflammasome activation, constituting independent or add-on signals damaging airway epithelium in asthma.

Co-infections with two or more respiratory viruses occur often and likely act as additional factors increasing airway epithelial damage. Patients with asthma are at a greater risk of developing respiratory failure as shown in the case of H1N1 influenza infection^80^. Thus, it has been somewhat surprising that during the current SARS-CoV-2 pandemic, epidemiological cohorts of COVID-19 patients from different geographical locations have resulted in partially contradictory observations that asthma is (USA, United Kingdom, Australia) or is not (Europe, China) a risk factor for SARS-CoV-2 infection and/or severity of COVID-19^12,13,16,81^. However, accumulating data from the bigger cohorts seem to agree that patients with asthma in general have increased risk of hospitalization due to COVID-19^11,82^, whereas patients with the more severe disease, patients in need of usage higher doses of control medications, or patients with frequent asthma exacerbations are also at higher risk of intensive care admission or death due to COVID-19^8,10,11,83–87^. We and others demonstrated that the expression of angiotensin-converting enzyme 2 (ACE2), the main SARS-CoV-2 receptor and other plausible points of entry, are not changed in patients with asthma^88^, even though different types of airway inflammation or inhaled steroids^89^ might modulate their expression and as such it seems unlikely that it would be the main reason for observed discrepancies^90^. Different geographical locations might represent variable levels and quality of environmental exposures such as viruses or allergens, which may interfere with the rate of SARS-CoV-2 infections and COVID-19 severity^22^. SARS-CoV-2, an enveloped, positive-sense, single-stranded (ss)RNA virus has been shown to be sensed, depending on the cell type, by MDA5^91^, RIG-I^91^ and NLRP3^92,93^. However, due to several evasion properties and encoding by non-structural (Nsp) and accessory proteins, such as Nsp1,6,12,13, various open read frames (ORFs), protein M, protein N and others, which antagonize interferon pathways on many levels, induction of IFNs by SARS-CoV-2 itself is reduced or delayed^30,31,94^ with the augmented proinflammatory mediator release^89^. Indeed, in our hands, SARS-CoV-2 infection alone led to an increased release of inflammasome-dependent and independent proinflammatory cytokines such as IL-18 and IL-33, respectively, as well as to the decrease of anti-viral CCL4 in controls and in asthma. However, only in asthma, infection with both viruses in the presence of HDM, resulted in an excessive pro-inflammatory signaling, which might be partially due to the observed aberrant response to HDM. In context of timing and possible clinical relevance it may mean that patients with asthma with pre-existing RV-infection and exposed to HDM might have excessive inflammasome-related damage and proinflammatory and in fact may succumb eventually to more severe COVID-19. However, the exact mechanisms of how HDM influences the balance between RIG-I inflammasome activation and IFN production, as well as the therapeutic approaches targeting described pathways, remain to be understood.

All in all, we showed here in vivo and in vitro that the lack of balance between activation of RIG-I inflammasome and the RIG-I-IFNs-axis in response to common respiratory viruses is an important driving factor of epithelial damage, lack of viral clearance and sustained airway inflammation in patients with asthma (Supplementary Fig. 8). Timely targeting of this abnormal response by the yet-to-be developed early therapeutics or even prophylactic approaches might provide in the future a beneficial strategy to prevent RV-induced exacerbations of asthma and potentially severe COVID-19.

## Methods

### Inclusion and ethics

Ethical permissions were obtained for all studies including humans and human-derived material presented in this study. In vivo rhinovirus infection in controls and patients with asthma was approved by the St. Mary’s Hospital Research Ethics Committee (United Kingdom), permission number 09/H0712/59 (observational trial registration number NCT01159782)^48^. The observational study on controls and patients with asthma referred as the Cohort SIBRO was approved by the Kantonale Ethik-Kommission Zürich (Switzerland), permission number KEK-ZH-Nr. 20212-0043, and the Bioethical Committee, Wroclaw Medical University (Poland), permission numbers KB-70/2013 and KB-567/2014^95^. The observational study referred as the Cohort A was approved by Jagiellonian University Bioethics Committee (Poland), with permission numbers KBET/68/B/2008 and KBET/209/B/2011^96^. All participants gave written informed consent. Further use and additional analyses were permitted and consented. Any other primary HBECs used for in vitro experiments were purchased from the commercially available sources. The current study included local researchers throughout the research process.

### Reagents

House dust mite (HDM) extract (HDM) (Allergopharma, Reinbek, Germany), house dust mite extract B (HDM B) (Citeq, York, UK), and Alternaria alternata (A. alternata) (Citeq, York, UK) were diluted in sterile 0.9% saline (NaCl) and stored in −20 °C. The concentration of the extracts used for the experiments was calculated according to the total protein content. Detailed description of the HDM and A. alternata extracts including protein, main allergens, and endotoxin content is presented in the Supplementary Table 7. Diesel Particulate matter (DEP) from an industrial forklift (Standard reference material 2975) was obtained from National Institute of Standards and Technology (Maryland, USA) and diluted in sterile ddH_2_0, followed by ultrasonication for 30 min, max power, RT (Elmasonic P, Elma, Germany). All commercially available antibodies and reagents used in the manuscript are described in the Supplementary Table 8. Mouse IgG2a monoclonal anti-human ICAM-1 antibody (antibody R6.5) was produced from the hybridoma cells (ATCC HB-9580, mouse hybridoma). The hybridoma cells were expanded in RPMI 1640 medium supplemented with 10% (v/v) IgG depleted fetal bovine serum, 2 mM L-glutamine, 1.25 g/L D-( + )-Glucose, 1 mM Sodium Pyruvate, 10 mM HEPES, 100 U/mL Penicillin-Streptomycin at 37 °C in 5% CO_2_. The IgG in culture media were affinity purified from the cell culture supernatant using a 1 mL HiTrap™ Protein G HP column (GE Healthcare, 29-0485-81). Eluted fractions were immediately neutralized and buffer exchanged into PBS using dialysis. The antibody was then filtered through a 0.22 µm filter and stored at 4 °C.

### Viruses

Rhinovirus A16 (RV-A16) was purchased from Virapur (San Diego, USA). UV-light inactivated RV-A16 (UV-RV-A16) was used as a control after an exposure to UV-light of the 254 nm at a 2 cm distance for 60 min. Cells were infected with RV-A16 or UV-RV-A16 at the multiplicity of infection (MOI) 0.1, 0.01 and 0.001 as determined by plaque assay. Briefly, HeLa cells were infected with the virus serial dilutions from 10^−2^ to 10^−8^ in duplicates. Seven days after infection, cells were fixed with formaldehyde solution and stained with 1% crystal violet in 20% ethanol and dH_2_0. Visible plaques were counted under a microscope.

Recombinant SARS-CoV-2 Wuhan-Hu-1 viral genome was generated from the synthetic DNA fragments produced by GenScript (Piscataway, USA) using the in-yeast transformation-associated recombination (TAR) cloning method, as previously described^97^. In-vitro transcription was performed for the linearized Yeast artificial chromosome (YAC), containing the cDNA of the SARS-CoV-2 genome, as well as a PCR amplified SARS-CoV-2 N gene using theT7 RiboMAX Large Scale RNA production system (Promega, Madison, USA) with m7G(5′)ppp(5′)G cap provided as described previously^98^. Transcribed, capped mRNA was subsequently electroporated into baby hamster kidney cells (BHK-21) expressing SARS-CoV-2 N protein. Co-culture of electroporated BHK-21 cells with susceptible Vero E6 cells produced passage 0 of SARS-CoV-2 virus. Passage 0 was used to infect Vero E6 cells to generate passage 1 working stocks, which were used for all experiments. Titers were determined using standard plaque assay, as described previously^97^.

### Experimental in vivo RV-A16 infection in humans

Experimental in vivo rhinovirus infection in 11 control individuals and 28 patients with asthma was performed and reported previously^48^. Briefly, non-smoking, non-atopic control individuals, and non-smoking mild/moderate patients with asthma without any recent viral illness and without serum neutralizing antibodies towards RV-A16, who passed inclusion criteria, underwent infection on day 0 with RV-A16 at the dose of 100 TCID_50._ Bronchial brushings, bronchial biopsies and bronchoalveolar lavage (BAL) fluid were collected around 2 weeks before and at 4 days after RV-A16 infection. Additionally, nasal lavage (NL) samples at the peak of RV-A16 infection were collected to assess RV-A16 infection rates. Only subjects who had sufficient remaining biobanked samples from the original study^48^ to be newly analyzed and/or subjects who had a successful infection in the lungs, as assessed by viral RNA copies by qPCR, were included in the BAL, NL, and biopsies analyses (*n* = 9 healthy control, *n* = 19 patients with asthma), and bronchial brushing microarray analysis (*n* = 7 healthy controls, *n* = 17 patients with asthma). The study received ethical approval from the St. Mary’s Hospital Research Ethics Committee (09/H0712/59). All participants gave written informed consent. Further use and additional analyses, including RNA microarray, were permitted and consented. All samples from the in vivo RV-A16 infection used in the current manuscript, were derived from the previous study^48^, and no additional in vivo RV-A16 infections were performed for the purposes of the current study. This observational cohort was registered at clinicaltrials.gov under the identifier NCT01159782. The clinical characteristics of the 9 control and 19 asthma study participants who had sufficient remaining samples to be analyzed in this study is presented in Supplementary Tables 1 and 9. Additionally, all details regarding the study cohorts are disclosed in Supplementary Table 10.

### In vitro air-liquid interface (ALI) cultures of primary human bronchial epithelium from healthy controls and patients with asthma

Control individuals and patients with asthma were enrolled in the ALL-MED Medical Research Institute, Wroclaw, Poland; the Pulmonary Division, University Hospital of Zurich, Switzerland (cohort SIBRO)^95^, or at the University Hospital, Jagiellonian University Medical College, Cracow, Poland (cohort A)^96^, as described previously. Briefly, bronchoscopy with epithelial cells brushings and BAL fluid collection was performed. The study was granted ethical permission from Switzerland and Poland (KEK-ZH-Nr. 20212-0043 – Kantonale Ethik-Kommission Zürich; KB-70/2013 and KB-567/2014 – Bioethical Committee, Wroclaw Medical University) or the Jagiellonian University Bioethics Committee (KBET/68/B/2008 and KBET/209/B/2011). Asthma diagnosis and severity were assessed according to the GINA guidelines^2^. All participants gave written, informed consent. Further use and additional analyses were permitted and consented. All samples used in the current manuscript for the in vitro experiments were derived from these two previous studies^95,96^, and no additional sampling was performed for the purpose of the current study. Clinical characteristics of the study participants is presented in Supplementary Table 9. Additionally, all details regarding the study cohorts are disclosed in Supplementary Table 10.

Primary Human Bronchial Epithelial cells (HBECs) were obtained from the above-listed cohort SIBRO and cohort A, or from the doctor-diagnosed asthma and control individuals from two independent commercial sources: Lonza (Basel, Switzerland), and Epithelix (Plan-les-Ouates, Switzerland). Characteristics of the HBECs used in the manuscript are presented in Supplementary Table 11.

HBECs, were cultured and differentiated in vitro in the air-liquid interface (ALI) conditions as described previously, with minor alterations of the previous protocol^96^. Briefly, cells from passage 2 were grown in 20 mL of bronchial epithelial basal medium (Lonza, Basel, Switzerland) supplemented with the SingleQuot Kit (Lonza, Basel, Switzerland) placed in 150 cm^2^ T-flask in humidified incubator at 37 °C with 5% CO_2_ for maximum 10 days, or until 80%–90% confluency. Next, cells were trypsinized (ThermoFisher Scientific, Waltham, USA) and seeded at a density of 1.5 × 10^5^ cells/well on the 6.5-mm-diameter polyester membranes with the 0.4 μm pore size and growth area of 0.33 cm^2^ (Costar, Corning, NY, USA; Oxyphen, Wetzikon, Switzerland) in 24-well culture plates. Bronchial Epithelial Growth Medium (BEGM) (Lonza, Basel, Switzerland) supplemented with the SingleQuot kit (Lonza, Basel, Switzerland), with an exception of the retinoic acid (ATRA, Sigma-Aldrich, St. Louis, USA) and triiodothyronine, was mixed in the 1:1 ratio with the Dulbecco modified Eagle medium (DMEM, Gibco, Thermofisher Scientific, Waltham, USA). Fresh all-trans ATRA (Sigma-Aldrich, Merck, Kenilworth, USA) was supplemented at a concentration of 15 ng/mL. Cells were grown submerged for 3–5 days in the apical medium and were in contact with the basolateral medium. After they obtained a full confluence, the apical medium was removed and cells were kept in the air-liquid interface (ALI) cultures for at least 21 days. BEGM/DMEM/ATRA medium was maintained only basolaterally to differentiate the HBECs. During the cell culture process, medium was exchanged every 2–3 days and, periodically, excess of produced mucus was removed from the wells. All experiments were performed on the fully differentiated HBECs from the same passage, between 21 and 28 days of ALI culture (Supplementary Fig. 3a, 5a).

### In vitro stimulations and rhinovirus A16 infection model in the primary HBECs

House dust mite (HDM) stimulation, followed by rhinovirus A16 (RV-A16) infection experiments were performed in the OptiMEM medium (LifeTechnologies, ThermoFisher Scientific, Waltham, USA). In vitro ALI-differentiated HBECs from control individuals and patients with asthma were treated apically with the HDM extract (Allergopharma, Reinbek, Germany) at a dose of 200 μg/mL of the total protein (or vehicle) in 200 μl OptiMEM on the apical side, and 600 μl of clear OptiMEM on the basolateral side (Supplementary Fig. 3a, 5a), in the humidified incubator at 37 °C with 5% CO_2_. After 24 h of HDM stimulation cells, were apically infected with RV-A16 at the MOI of 0.1 or as otherwise specified or stimulated with UV-RV-A16 at the same MOI and cultured in the humidified incubator at 34.5 °C with 5% CO_2_ for the next 24 h (Supplementary Fig. 3a, 5a). Next, cell supernatants (apical and basolateral), RNA, and protein cellular lysates were collected and stored in −80 °C. Some cells were fixed with 4% PFA (Fluka/Sigma Aldrich Buch, Switzerland) and were stored wet at 4 °C for 1–2 weeks before the subsequent confocal analyses. All doses and time-points used for the final experiments were based on the preliminary dose-dependent and time-course experiments. Briefly, two different HDM extracts: main HDM extract used in the manuscript (Allergopharma, Reinbek, Germany) at the dose of 200 μg/mL, and HDM extract B (Citeq, York, UK) at the dose of 200 and 100 μg/ml were investigated (Supplementary Fig. 5b, 6a-b). RV-A16-infection of HBECs from patients with asthma was performed in 6 h and 24 h time-points (Supplementary Fig. 3b). Lastly, RV-A16 infection at the MOI 0.001, MOI 0.01, and MOI 0.1 was investigated (Supplementary Fig. 3c). Based on the secretion of the mature IL-1β, HDM extract from the Allergopharma, Reinbek, Germany at the dose of 200 μg/mL, RV-A16 MOI 0.1 and the 24 h time-point were chosen, and are presented through the manuscript, if not mentioned differently. Additionally, cells were stimulated with heat-inactivated HDM (H-HDM) (Allergopharma, Reinbek, Germany) at 200 μg/mL of protein content, A. alternata (Citeq, York, UK) at 25 μg/mL of protein content or DEP (NIST®SRM®2975, National Institute of Standards and Technology, Maryland, USA) at 25 μg/mL, following the same experimental design as for HDM stimulations. H-HDM was prepared by heating up for 30 min in 65 °C. For the experiments with inhibitors, 40 μM of the caspase-1 inhibitor: ac-YVAD-cmk (Acetyl-tyrosine-valine-alanine-aspartate-chloromethyl ketone, Invivogen, San Diego, USA), 1μM of the IKKε/TBK1 inhibitor: BX795 (N-[3-[[5-iodo-4-[[3-[(2-thienylcarbonyl)amino]propyl]amino]−2-pyrimidinyl]amino]phenyl]−1-Pyrrolidinecarboxamide hydrochloride, Sigma Aldrich, Merck, Kenilworth, USA), or 1 μM of the NLRP3 inflammasome inhibitor: MCC950 (C20H23N2NaO5S, Avistron, Bude, UK) or appropriate vehicle controls were used apically and basolaterally, 24 h prior RV-A16 infection. To block ICAM-1, a receptor responsible for RV-A16 infection of HBECs, anti-ICAM-1 antibodies were added to the apical and basolateral compartment, 3 h prior RV-A16 infection at the dose of 10 ug/mL (Supplementary Fig. 3a).

### In vitro co-infection model in primary HBECs

The in vitro ALI-differentiated MucilAir cultures (Epithelix, Plan-les-Ouates, Switzerland) from primary human bronchial epithelium obtained from 6 control individuals and 7 patients with asthma (Supplementary Table 11) were cultured for 7 days in the MucilAir Medium (Epithelix, Plan-les-Ouates, Switzerland) in ALI conditions, with basolateral medium changed every other day or ALI-differentiated in house as described above. On the day of the experiment, performed in a biosafety level 3 (BSL3) laboratory, cells were washed with warm PBS to remove an excess of mucus. The experiment was performed in the OptiMEM medium (LifeTechnologies, ThermoFisher Scientific, Waltham, USA) in the volume of 250 μl on the apical, and 600 μl on the basolateral side. Throughout the whole experiment cells were kept in the humidified incubator at 37 °C with 5% CO_2._ First, HBECs were stimulated apically with 200 μg/mL of protein content of HDM extract or vehicle. 24 h after HDM stimulation, cells were apically infected with/without RV-A16 at the MOI of 0.1. After the next 24 h, HBECs were apically infected with/without SARS-CoV-2 at the MOI of 0.1. Finally, 48 h after SAR-CoV-2 infection experiment was harvested (Fig. 6a). Next, an additional approach was performed, where 24 h after HDM stimulation, cells were apically infected with/without SARS-CoV-2 at the MOI of 0.1. After the next 48 h, HBECs were apically infected with/without RV-A16 at the MOI of 0.1 for another 24 h (Supplementary Fig. 7b–d). In order to inactivate SARS-CoV-2, all collected supernatants were treated at 65 °C for 30 min. Cells were fixed in 4% PFA for at least 20 min. Inactivated supernatants were frozen in −80 °C until further analyses. For RNA analyses, insert with the fixed cells were preserved in RNAlater (Qiagen, Hilden, Germany), left overnight in 4 °C, and stored in −20 °C in the new, dry tube. In order to perform confocal staining, inserts with the fixed cells were snap frozen in the Clear Frozen Section Compound (FSC22, Leica, Wetzlar, Germany).

### THP-1 cell culture

THP-1-XBlue cells (Invivogen, San Diego, USA) were defrosted in 32 mL of RPMI-1640 medium (Sigma-Aldrich, St. Louis, USA) supplemented with the Penicillin/Streptomycin/Kanamycin, MEM vitamins, Na-Pyruvate/MEM Non-essential Amino Acid Solution and heat-inactivated FCS (cRPMI medium) in the 75cm^2^ T-flask, and cultured for 1 day in the humidified incubator at 37 °C with 5% CO_2_. In the following day, cells were counted, checked for viability (98%) and transferred to the 12-well cell cultures plate (0.5 mio cells/well in 1 mL of cRPMI medium). Next day, cells were stimulated with LPS (100 ng/mL, Invivogen, San Diego, USA) or vehicle for 4 h followed by 2 mM ATP or vehicle (Invivogen, San Diego, USA) for 20 min. Cytospins (250 x g, 3 min, Shandon Cytospin 2, Marshall Scientific, Hampton, USA) were prepared, and cells were immediately fixed with 4% PFA (Fluka/Sigma Aldrich, Buchs, Switzerland), and stored in wet chamber before the confocal staining.

### Immunoassays

#### ELISA and MSD multiplex

Secreted IL-1β in majority of in vitro experiments was measured with the ELISA kit (R&D Systems, McKinley Place, USA), according to the manufacturers instruction, on Mithras LB940 (Berthold Technologies) with MicroWin 2010 software. The detection limit for the kit is 3.91 pg/mL. IL-1β in BAL fluid from control individuals and patients with asthma was measured using mesoscale discovery platform (MSD, Kenilworth, USA) kits as described previously^95^. Additionally, BAL samples from controls and patients with asthma before and after experimental intranasal RV-A16 infection in vivo were analyzed with V-PLEX human IL-1β Kit (MSD, Kenilworth, USA), according to the manufacturer’s instructions, on MSD Discovery Workbench 4.0.12. V-PLEX IL-1β is presented as arbitrary units (arb. units) corresponding to percentage of total protein concentration measured by BCA (ThermoFisher Scientific, Waltham, USA) and multiplied by factor 1000000.

#### Proximity extension assay (PEA) targeted proteomics

Protein expression in the apical compartments of the HBECs were measured using the proximity extension assay targeted proteomics technology (Olink, Stockholm, Sweden) on Fusion FX (Vilber) and Fluidigm Real Time PCR Analysis and Olink NPX Manager. Targeted 96-proteins Inflammation, Immune Response and Immuno-Oncology panels were used according to the manufacturer’s instructions with the suggested adaptations to the cell cultures conditions. Apical compartments from RV-A16 + SARS-CoV-2 model were analyzed with the human Target 48 Cytokine Panel (Olink, Uppsala, Sweden). Final results for 96-plex assay are reported as Normalized Protein eXpression (NPX), an arbitrary unit in log_2_-scale. Results from the Target 48 Cytokine Panel are in pg/mL.

#### Western Blotting

Western Blotting experiments from the cell lysates and the apical supernatants were performed as previously described^96,99^. Briefly, cells were lysed in RIPA Lysis and Extraction buffer (ThermoFisher Scientific, Waltham, USA) supplemented with the protease inhibitor (Roche, Merck, ThermoFisher Scientific, Waltham, USA) for 15 min on ice, centrifuged (15 min, full speed, 4 °C), and debris-free protein lysates were frozen in −80 °C for further analyses. Protein concentration was assessed with the BCA kit (ThermoFisher Scientific, Waltham, USA), according to the manufacturer’s instructions. Protein from the apical supernatants was precipitated with 1 volume of methanol (Fisher Scientific, Reinach, Switzerland) and ¼ volume of chloroform (Merck Millipore, Burlington, USA) as described previously^99^. Equal amounts of cell lysate proteins (10–20 μg) were loaded on the 4–20% Mini-PROTEAN TGX Gel (Bio-Rad, Hercules, USA) or 4–20% SuperPAGE gel (GenScript, Leiden, Netherlands) in Tris/Glycine/SDS buffer (Bio-Rad Lab, Hercules, USA) or MOPS buffer (GenScript, Leiden, Netherlands). After electrophoresis, the proteins were transferred to the nitrocellulose membranes (Bio-Rad, Hercules, USA or Advansta, San Jose USA) using the Trans-Blot Turbo Blotting System (Bio-Rad, Hercules, USA) or eBlot L1 Protein Transfer System (GenScript, Leiden, Nederlands). The membranes were blocked with 5% nonfat milk in 0.1% Tween20 PBS (PBST) for 1 h, washed with 10x PBST, and incubated with the primary antibodies for overnight in 4 °C. Dilutions of primary antibodies used for the analyses of the cell lysates: 1:100 IL-1β (R&D Systems, McKinley Place, USA), 1:200 RIG-I (Santa Cruz Biotechnology, Dallas, USA), 1:1000 ASC (Santa Cruz Biotechnology, Dallas, USA), 1:1000 caspase-1 (Cell Signaling, Danvers, USA), and 1:1000 NLRP3 (Adipogen, San Diego, USA). The membranes were subsequently washed in 10xPBST and incubated with an appropriate secondary antibody conjugated with the horseradish peroxidase (HRP) (1:10,000 dilution) (Jackson ImmunoResearch, West Grove, USA; Santa Cruz Biotechnology, Dallas, USA) for 1 h at room temperature. β-actin expression was determined with HRP-conjugated antibodies in 1:25,000 dilution (Abcam, Cambridge, UK). Protein samples precipitated from the apical supernatants were analyzed with the use of goat anti-IL-1β antibodies (1:1000, R&D Systems, Minneapolis, USA) and HRP conjugated anti-goat (1:10,000, Santa Cruz Biotechnology, Santa Cruz, USA) antibodies. After washing with 10xPBST, the blots were developed with the SuperSignal West Femto Kit (ThermoFisher Scientific, Waltham USA) or WesternBright Quantum/Sirius HRP substrate (Advansta, San Jose, USA) and visualized on the Luminescent Image Analyzed LAS-1000 (Fujifilm, Tokyo, Japan) or the Fusion FX (Vilber, Collegien, France). To assess more than one protein, the membranes were stripped with the Restore PLUS Western Blot Stripping Buffer (ThermoFisher Scientific, Waltham, USA). Quantification of the protein expression was performed in Fiji Software (version 1.0.0-rc-49/1.51d)^100^. Briefly, an area of the peak of the protein of interest was measured in triplicates, and average was used to calculate the ratio between expression of the protein of interest and β-actin (protein/β-actin). Protein/β-actin ratio from HBECs from control individuals and patients with asthma upon HDM stimulation, RV-A16 infection, or both was further normalized to the vehicle control condition from control individuals, and log transformed with Y = log(Y) function.

#### Co-immunoprecipitation

For co-immunoprecipitation cells were lysed with the Lysing Buffer (1 μM DTT + 10% Triton X100) in ddH_2_0 supplemented with the protease inhibitor (Roche, Merck, ThermoFisher Scientific, Waltham, USA) for 15 min on ice, centrifuged (15 min, full speed, 4 °C), and the debris-free protein lysates were frozen in −80 °C for further analyses. Protein concentration was assessed with the BCA kit (ThermoFisher Scientific, Waltham, USA) according to the manufacturer’s recommendation. 100 μg of the pooled protein was pre-cleared with 100 μl of Protein A beads (Bio-Rad, Hercules, USA), magnetized, and incubated with 10 μg of anti-ASC antibodies (Santa Cruz Biotechnology, Santa Cruz, USA) overnight at 4 °C, followed by ASC immunoprecipitation with 100 μl of Protein A beads (Bio-Rad, Hercules, USA) for 2 h in room temperature. Co-IP samples and input (protein not bound to the beads) were collected and together with the cell lysates were further analyzed with the Western Blot protocol with the use of mouse anti-human RIG-I antibodies (1:200, Santa Cruz Biotechnology, Santa Cruz, USA) or rabbit anti-human MDA5 antibodies (1:1000, Abcam, Cambridge, UK) and HRP conjugated anti-mouse or anti-rabbit antibodies (1:10,000, Jackson ImmunoResearch, West Grove, USA).

#### mRNA isolation and RT-PCR in HDM and RV-A16 model

Cells were lysed on ice with the RLT buffer (Qiagen, Hilden, Germany) supplemented with β-mercaptoethanol (Sigma-Aldrich, St. Louis, USA), and stored at −80 °C until further analyses. mRNA isolation was performed with the RNeasy Micro Kit (Qiagen, Hilden, Germany) according to the manufacturer’s instructions. Quality and quantity of isolated RNA was assessed by the Nanodrop 2000 (ThermoFisher Scientific, Waltham, USA). Reverse transcription was performed with RevertAid RT kit (ThermoFisher Scientific, Waltham, USA) with random hexamers, according to the manufacturer’s recommendations. Gene expression (5–10 ng of cDNA/well) was assessed by RT-PCR using the SYBR Green/ROX qPCR Master Mix (ThermoFisher Scientific, Waltham, USA), performed on the QuantStudio 7 Flex Real-Time PCR System (ThermoFisher Scientific, Waltham, USA). The sequences of used primers are summarized in Supplementary Table 12. Gene expression was normalized to the housekeeping gene, elongation factor 1α (EEF1A), and presented as a relative quantification calculated with the ΔΔCt formula, as described previously^46^. Depending on the analyses, data were calibrated according to the vehicle condition from HBECs from control individuals, or vehicle condition calculated separately for control individuals and patients with asthma. Data are presented as 2^-ΔΔCt^ values, or percentage change normalized to the specified condition.

#### mRNA isolation and RT-PCR in co-infection in vitro model

Samples preserved in the RNAlater, as described above, were immersed in the increasing concentrations of ethanol (30% up to 100%, increasing every 10%). After this initial step, RNA was isolated with use of RecoverAll kit (Invitrogen, Waltham, USA) according to the manufacturer’s protocol. Isolated mRNA was concentrated with the use of SpeedVac (DNA Speed Vac, DNA110, Savant, Hayanis, USA) for 1 h in the ambient temperature. Sample concentration did not affect its quality, as measured with use of NanoDrop One^C^ (ThermoFisher Scientific, Waltham, USA). Reverse transcription was performed with use of the SuperScript IV VILO Master Mix (Thermofisher Scientific, Waltham USA), according to the manufacturer’s recommendations. Gene expression (5 ng of cDNA/well) was assessed by RT-PCR using (i) SYBR Green PCR Master Mix (ThermoFisher Scientific, Waltham, USA) for *DDX58*, *IFNB*, *IFNL1*, *IL1B*, *MDA5*, and (ii) TaqMan assays for RV-A16 and SARS-CoV-2 *Protein N*, *Protein S*, *ORF1AB* detection (ThermoFisher Scientific, Waltham, USA) and was performed on the QuantStudio 7 Flex Real-Time PCR System (ThermoFisher Scientific, Waltham, USA). The sequences of used primers are summarized in Supplementary Table 12. Gene expression was normalized to the elongation factor 1α (EEF1A) and presented as a relative quantification calculated with -ΔΔCt formula, compared to the vehicle condition separately for controls and patients with asthma, as described previously^46^. SARS-CoV-2 viral RNA in presented as 2^-ΔΔCt^ values averaged from the expression of *N protein*, *S protein* and *ORF1AB* in each condition.

#### Rhinovirus quantification in in vivo RV-A16 infection in humans

Rhinovirus infection in control individuals and patients with asthma after experimental RV-A16 infection in vivo was performed in the nasal lavages (peak of infection) and BAL fluid (4 days post infection) with use of qPCR, as previously described^48^. Results are presented as viral RNA copies in 1 mL.

#### Confocal microscopy

Cells were fixed on the inserts with 4% paraformaldehyde (Fluka/Sigma Aldrich, Buch, Switzerland) for 10 min, permeabilized with detergent (PBS + 0.1% TritonX100 + 0.02% SDS) for 5 min and blocked with 10% goat serum (Dako, Agilent, Santa Clara, USA) in 1% BSA/PBS for 60 min at room temperature. All antibodies were diluted in 4% goat serum + 1% BSA/PBS, and cells were stained from apical and basolateral sides with 100 μl of antibodies working solution. Samples were stained for ASC (2 μg/mL, mouse anti-ASC, Santa Cruz Biotechnology, Santa Cruz, USA), IL-1β (10 μg/mL, mouse anti- IL-1β, Abcam, Cambridge, UK), and RIG-I (2 μg/mL, mouse anti-RIG-I, Santa Cruz Biotechnology, Santa Cruz, USA) for 60 min at room temperature. Proper mouse isotype controls, in the corresponding concentrations were used to control for unspecific binding. (Dako, Agilent, Santa Clara, USA). Subsequently, samples were incubated with the goat anti-mouse Alexa Fluor 488 (for ASC), and the goat anti-mouse Alexa Fluor 546 (for IL-1β and RIG-I) secondary antibodies at the concentrations of 1:2000 (Invitrogen, Waltham, USA) for 60 min at room temperature. Samples were mounted in the ProLong Gold mounting medium containing DAPI (Life Technologies, Carlsbad, USA) according to the manufacturer’s instructions, analyzed with a Zeiss LSM780 confocal microscope (Zeiss, Oberkochen, Germany) and Zen 3.2 Blue Edition Software (Zeiss, Oberkochen, Germany). All pictures were taken at the 40x magnification and are presented as maximal projection (orthogonal projection) from z-stacks (3-22 for ASC, 4 for IL-1β and RIG-I). ¼ of the original picture is shown on the figures, with appropriate scale bar and further magnification of the area of interest. Additionally, to quantify ASC speck formation specks from 3–5 pictures per condition were counted in duplicates and averaged.

Differentiated HBECs upon HDM + RV-A16 stimulation and THP-1 cells stimulated with LPS + ATP were used for NLRP3 and Occludin visualization, whereas HBECs from RV-A16 + SARS-CoV-2 model were used for ACE2 and SARS-CoV-2 Protein N staining. Following fixation in 4% (wt/vol) PFA in PBS (Fluka, Fluka/Sigma Aldrich, Buchs, Switzerland), THP-1 cytospins were lined with the PAP pen (Sigma Aldrich, St. Louis, USA), HBECs were stained on the insert, whereas ¼ of the ALI insert from RV-A16 + SARS-CoV-2 model were prepared for cryosections by freezing in Clear Frozen Section Compound (FSC22, Leica, Wetzlar, Germany), cut at 6 µm in a cryostat (LEICA CM3050S, Leica Microsystems, Wetzlar, Germany) and mounted on *SuperFrost Plus*^*TM*^ glass slides (Menzel, ThermoFisher, Waltham, USA). Samples were incubated in the blocking solution (10% normal goat serum, 1% bovine serum albumin and 0.2% TritonX-100 in PBS) (Dako, Agilent, Santa Clara, USA) for 1 h at room temperature. Primary antibodies for NLRP3 (5 µg/mL, mouse anti-NLRP3, Adipogen, San Diego, USA), Occludin (2.5 µg/mL, mouse anti-Occludin, ThermoFisher, Waltham, USA), ACE-2 (2 µg/mL, rabbit anti-ACE2, Abcam, Cambridge, UK), and SARS-CoV-2 Protein N (1 µg/mL, mouse anti-Protein N, ThermoFisher, Waltham, USA) diluted in blocking solution (1:1 in PBS) and incubated at 4 °C overnight. Proper isotype controls, in the concentrations corresponding to the antibodies, were used (Dako, Agilent, Santa Clara, USA). Following three washing steps in 0.05% Tween20 in PBS, secondary antibodies with DAPI (1 µg/mL, Sigma Aldrich, St. Louis, USA) in diluted blocking solution (1:1 in PBS) were applied for 2 h at room temperature in the dark. Sections have been washed three times in 0.05%Tween20 in PBS before mounting with Fluoromount (Sigma Aldrich, St. Louis, USA). Image acquisition was performed with Zeiss LSM780 (Zeiss, Oberkochen, Germany), by using 40x objective and ZEN software (Zeiss, Oberkochen, Germany). ImageJ/Fiji^100^ was used for tale scan stitching and image analysis.

Bronchial biopsies were collected from the control individuals and patients with asthma at baseline and 4 days after in vivo RV-A16 infection. Biopsies were fixed and embedded in the paraffin blocks, sections were cut, and placed on the glass slides as described previously^101^. Prepared slides were baked 30 min in 65 °C, followed by the deparaffinization with xylol (2 × 10 min), graded isopropanol (2 × 3 min 100%, 2 × 3 min 96% and 3 min 70%), and rehydration (2 × 5 min in H_2_0). Samples were boiled in the sodium citrate buffer (10 mM sodium citrate with 0.05% Tween 20 in PBS at pH6) in the pressure cooker for 4 min, as described previously^102^. Samples were permeabilized and blocked with the Perm/Block Buffer (1%BSA + 0.2%TritonX100 + 10% goat serum in PBS) for 25 min in room temperature. All antibodies were diluted in 4% goat serum + 0.05% Tween20 in PBS, and 50 μl of antibodies per sample were used. Primary antibodies for IL-1β (10 μg/mL, mouse anti-IL1β, Abcam, Cambridge, UK), caspase-1 (1:50, rabbit anti-caspase 1, Cell Signaling, Danvers, USA), and RIG-I (1:250, mouse anti RIG-I, Santa Cruz Biotechnology, Santa Cruz, USA) were incubated in the wet chamber overnight at 4 °C. Appropriate mouse and rabbit isotype controls, in the concentrations corresponding to the antibodies, were used (Dako, Agilent, Santa Clara, USA). Subsequently, samples were incubated with the goat anti-rabbit Alexa Fluor 488 (for caspase-1), and the goat anti-mouse Alexa Fluor 546 (for IL-1β and RIG-I) secondary antibodies for 60 min in room temperature in the concentration of 1:1000 (Invitrogen, Waltham, USA). After 3 min incubation in 1% PFA in room temperature, samples were mounted in the ProLong Gold mounting medium containing DAPI (Life Technologies, Carlsbad, USA) according to the manufacturer’s instructions, analyzed with a Zeiss LSM780 (Zeiss, Oberkochen, Germany) and Zen Software (Zeiss, Oberkochen, Germany). All pictures were taken at the 40x magnification and are presented as the maximal projection (orthogonal projection) from 4 z-stacks, with appropriate scale bar. For the quantification, 10 equal squares from epithelial areas of the tissue (assessed using Haematoxylin and Eosin staining of the adjacent slice) from stained and isotype control samples were measured for signal intensity and averaged. Isotype control signal was further subtracted from the intensity of the stained samples. and values from 10 squares per sample were presented as the mean fluorescent intensity (MFI) of the protein expression.

#### Transcriptome analyses

Next generation sequencing (NGS) from the differentiated human bronchial epithelial cells (HBECs) from control individuals and patients with asthma (cohort A) was performed as previously described^90^. Briefly, total RNA was isolated with a RNeasy Plus Micro Kit (Qiagen, Hilden, Germany). Library was prepared with the TruSeq Stranded mRNA Sample Prep Kit (Illumina, San Diego, USA), and sequenced on the Illumina HiSeq 4000 platform. Description of the study subjects is presented in Supplementary Table 9.

HBECs from 6 control individuals and 6 patients with asthma, infected with RV-A16 in the MOI 10 for 24 h were harvested and sequenced with the use of Illumina HiSeq 2000 platform, as described previously^51^. The mRNA expression data are publicly available at the Gene Expression Omnibus platform (https://www.ncbi.nlm.nih.gov/) under the accession number: GSE61141^51^.

Bronchial brushings from control individuals and patients with asthma before and after experimental RV-A16 infection in vivo were analyzed by Affymetrix HuGene 1.0 array according to the manufacturer’s instructions and Transcriptome Analysis Console v4.0 (Santa Clara, United States)^54^.

#### Statistics and Reproducibility

Data delivered from the experimental in vivo RV-A16 infection in healthy controls and patients with asthma, performed only once by Jackson et al^48^, represent biological replicates obtained from one experimental infection with RV-A16. The number of biological samples is stated in each figure legend. All available samples from the patients with the successful RV infection detected in the BAL, remaining in the biobank after the original study^48^ were included in the present study. Investigators were not blinded during analyses. In vitro experiments were performed in multiple biological replicates (disclaimed in detail in figure legends) in at least three independent experiments. No statistical method was used to predetermine the sample size. No data were excluded from the analyses. The in vitro experiments were not randomized. Due to the characteristics of the study, investigators were not blinded during experiments and outcome assessments. Quantification of the western blot and confocal images were performed in a blinded manner.

Distribution (normality) of the data was assessed with Shapiro–Wilk test. One-way ANOVA (Kruskal–Wallis test), RM one-way ANOVA (Friedman test) or mixed-effects model tests were performed for more than three groups comparisons depending on the data relation (paired/not-paired) and distribution (normal/not-normal). Two-tailed paired/not-paired t-test or Wilcoxon/*U*-Mann–Whitney tests were performed for two groups comparisons depending on the data relation and distribution. Correlation between viral, RV-A16 and SARS-CoV-2 loads were calculated with Spearman’s rank correlation test. The data are presented as the mean ± SEM, with the number of samples in each experiment indicated in the figure description. IL-1β expression in BAL fluid from cohort SIBRO was analyzed with the Welsh’s test. All differences were considered significant when *p* ≤ 0.05. Statistical analysis was performed with the Prism 9 software (Redmond, USA).

Transcriptome data were processed with the workflow available here [https://github.com/uzh/ezRun], with the significance threshold for differentially expressed genes set to *p*-value < .05 calculated for the entire gene lists in each project using the edgeR R package^103^. Microarray data was analyzed by the following Bioconductor microarray analysis workflow [https://www.bioconductor.org/packages/release/workflows/vignettes/arrays/inst/doc/arrays.html]. Power of the microarray analyses calculated by G*power 3.1 [https://www.psychologie.hhu.de/arbeitsgruppen/allgemeine-psychologie-und-arbeitspsychologie/gpower] was sufficient to detect major differences. Differentially expressed probe was identified by the limma R package with empirical Bayes estimation. Threshold for significance for transcriptome data presented on the figures are as follows: *p*-value: *<0.05; **<0.005; ***<0.0005, ****<0.00005. Heatmaps display normalized gene expression across the gene in the groups (row normalization). Additionally, enrichment analysis of the most significant process networks in bronchial brushings after in vivo RV-A16 infection in patients with asthma when compared with bronchial brushings from control individuals after in vivo RV-A16 infection (Asthma (RV-A16 infection vs baseline) vs Control (RV-A16 infection vs baseline)) was performed with Metacore software version 19.2.69700 (Thomson Reuters, Toronto, Canada) (Supplementary Table 2). Top 100 genes upregulated after RV-A16 infection in the HBECs from control individuals and patients with asthma from GSE61141^51^ were analyzed for the enriched pathways using Metacore software version 20.3.70200 (Thomson Reuters, Toronto, Canada) (Supplementary Table 3). Inflammasome-mediated immune response and antiviral response gene sets were curated from GSEA and MSigDB Database (Broad Institute, Massachusetts Institute of Technology, and Reagent of the University of California, USA). Full sets of analyzed genes are described in Supplementary Table 13.

Proximity Extension Assay (PEA) normalized protein expression (NPX) data were analyzed with the use of the internal Shiny App Olink data analysis toolkit. The statistical comparison of protein expression between groups was performed with the Bioconductor limma package^104^. The fold change and p-value were estimated by fitting a linear model for each protein. Proteins with *p*-value <0.05 were considered significant. Additionally, for Target 96 Inflammation panel data are presented as: (i) heatmaps of curated signatures of inflammasome-mediated immune responses and antiviral responses (Supplementary Table 13) and (ii) protein interactions and pathways analysis prepared using the STRING (version 11.0)^105^, and further processed with the Cytoscape software (version 3.8.2)^106^ (Supplementary Table 5). The list of all proteins available for PEA measurements at the moment of the current analysis, that were used as a background reference for STRING analyses for targeted proteomics data is presented in Supplementary Table 14. Protein interactions and pathways analysis for quantitative PEA were prepared using the STRING (version 11.5)^105^, and further processed with the Cytoscape software (version 3.9.1)^106^

### Reporting summary

Further information on research design is available in the Nature Portfolio Reporting Summary linked to this article.

## Supplementary information

Supplementary Information

Reporting Summary

## Data Availability

Transcriptome data from bronchial brushings from control individuals and patients with asthma infected in vivo with RV used in the study have been deposited in the NCBI GEO database and are available under accession number: GSE185658. Publicly available RNAseq data GSE61141 were downloaded from the NCBI gene expression omnibus. All other data generated in the study, including inflammasome-mediated immune response and antiviral response gene sets curated from GSEA, and MSigDB Database (Broad Institute, Massachusetts Institute of Technology, and Reagent of the University of California, USA) are provided in Supplementary Information and Source data files. Source data are provided with this paper.

## Source data

Source Data

## Code availability

The codes for transcriptome data analysis are available here: NGS [https://github.com/uzh/ezRun], microarray^107^ [https://github.com/ge11232002/p1688-Ula]. Code for Proximity Extension Assay (PEA) data analysis is available here [https://github.com/ge11232002/OlinkR].
