# Supplementary material for: Rhinovirus-induced epithelial RIG-I inflammasome suppresses antiviral immunity and promotes inflammation in asthma and COVID-19
Source: Nat Commun. 2023 Apr 22;14:2329. doi: 10.1038/s41467-023-37470-4 (PMC10122208; doi:10.1038/s41467-023-37470-4)

Rhinovirus-induced epithelial RIG-I inflammasome suppresses antiviral immunity and promotes inflammation in asthma and COVID-19

Radzikowska Urszula[1,2,3], Eljaszewicz Andrzej[1,2,3], Tan Ge[1,4], Stocker Nino[1], Heider Anja[1], Westermann Patrick[1], Steiner Silvio[5,6,7], Dreher Anita[1,2], Wawrzyniak Paulina[1,2,8,9], Rückert Beate[1], Rodriguez-Coira Juan[1,10,11], Zhakparov Damir[1], Huang Mengting[1], Jakiela Bogdan[12], Sanak Marek[12,] Moniuszko Marcin[3,13], O`Mahony Liam[1, 14], Jutel Marek[15,16], Kebadze Tatiana[17,18], Jackson J David[19,20], Edwards R Michael[17,21], Thiel Volker[5,22], Johnston L Sebastian[17, 21, 23], Akdis A Cezmi[1,2,&], Sokolowska Milena[1,2,&,*]

[&] These authors contributed equally

* Corresponding author (milena.sokolowska@siaf.uzh.ch)

[1] Swiss Institute of Allergy and Asthma Research (SIAF), University of Zurich, Herman-Burchard-Strasse 9, 7265 Davos Wolfgang, Switzerland

[2] Christine Kühne – Center for Allergy Research and Education (CK-CARE), Herman-Burchard-Strasse 1, 7265 Davos Wolfgang, Switzerland

[3] Department of Regenerative Medicine and Immune Regulation, Medical University of Bialystok, Waszyngtona 13 Str., 15-269 Bialystok, Poland

[4] Functional Genomics Center Zurich, ETH Zurich/University of Zurich Winterthurerstrasse 190, 8057 Zurich, Switzerland

[5] Institute of Virology and Immunology (IVI), Laenggassstrasse 122, 3012 Bern, Switzerland

[6] Department of Infectious Diseases and Pathobiology, Vetsuisse Faculty, University of Bern, Laenggassstrasse 122, 3012 Bern, Switzerland

[7] Graduate School for Cellular and Biomedical Sciences, University of Bern, Mittelstrasse 43, 3012 Bern, Switzerland

[8] Division of Clinical Chemistry and Biochemistry, University Children`s Hospital Zurich, Raemistrasse 100, 8091 Zurich, Switzerland

[9] Children`s Research Center, University Children`s Hospital Zurich, Raemistrasse 100, 8091 Zurich, Switzerland

[10] IMMA, Department of Basic Medical Sciences, Facultad de Medicina, Universidad San Pablo-CEU, CEU Universities Madrid, C. de Julian Romea 23, 28003 Madrid, Spain

[11] Centre for Metabolomics and Bioanalysis (CEMBIO), Department of Chemistry and Biochemistry, Facultad de Farmacia, Universidad San Pablo-CEU, CEU Universities Madrid, Urb. Monteprincipe 28925 Alcorcon, Madrid, Spain

[12] Department of Internal Medicine, Jagiellonian University Medical College, M. Skawinska 8 Str., 31-066 Krakow, Poland

[13] Department of Allergology and Internal Medicine, Medical University of Bialystok, M. Sklodowskiej-Curie 24A Str., 15-276 Bialystok, Poland

[14] Department of Medicine and School of Microbiology, APC Microbiome Ireland, University College Cork, College Rd, T12 E138 Cork, Ireland

[15] Department of Clinical Immunology, Wroclaw Medical University, wyb. Lidwika Pasteura 1 Str., 50-367 Wroclaw, Poland

[16] All-MED Medical Research Institute, Gen. Jozefa Hallera 95 Str., 53-201 Wroclaw, Poland

[17] National Heart and Lung Institute, Imperial College London, Guy Scadding Building, Cale Street, London SW3 6LY, United Kingdom
[18] Department of Infectious Diseases, Imperial College London, School of Medicine, St Mary`s Hospital, Praed Street, London W21NY, United Kingdom
[19] Guy's Severe Asthma Centre, School of Immunology & Microbial Sciences, King's College London, Strand, London WC2R 2LS, United Kingdom
[20] Guy's & St Thomas' NHS Trust, St Thomas` Hospital, Westminster Bridge Rd, London SE1 7EH, United Kingdom
[21] Asthma UK Centre in Allergic Mechanisms of Asthma, Norfolk Place, London W2 1PG, United Kingdom
[22] Multidisciplinary Center for Infectious Diseases, University of Bern, Hallerstrasse 6, 3012 Bern, Switzerland
[23] Imperial College Healthcare HNS Trust, The Bays, S Wharf Rd, London W2 1NY, United Kingdom

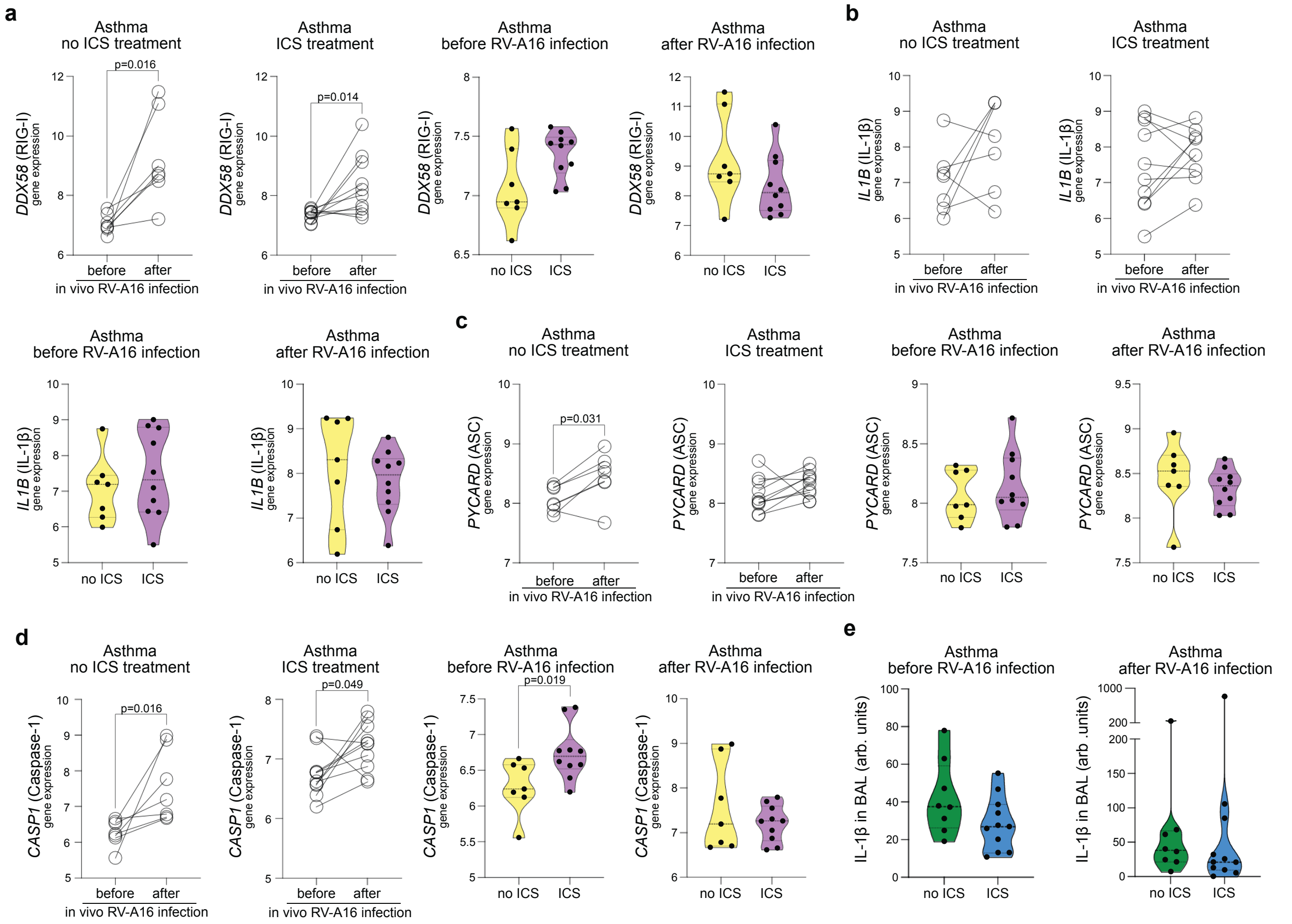

Supplementary Figure 1

Rhinovirus-induced inflammasome-mediated immune responses in epithelium of lower airways in asthma do not depend on patients' regular treatment.

Gene expression of **a** *DDX58* (RIG-I), **b** *IL1B* (IL-1β), **c** *PYCARD* (ASC), and **d** *CASP1* (caspase-1) in bronchial brushings from patients with asthma 14 days before, and 4 days after in vivo RV-A16 infection, and **e** secreted IL-1β protein in BAL, stratified according to the treatment with (n=10 for gene expression, n=11 for IL-1β in BAL) and without (n=7 for gene expression, n=8 for IL-1β in BAL) inhaled corticosteroids (ICS). (n) indicates the number of biologically independent samples examined over one in vivo RV-A16 infection. Analyzed with two-sided Wilcoxon or U-Mann Whitney test, as appropriate. Source data are provided as Source Data files. *Arb. units*, arbitrary units; *BAL*, bronchoalveolar lavage fluid; *ICS*, inhaled glucocorticosteroids, *RV-A16*, rhinovirus A16.

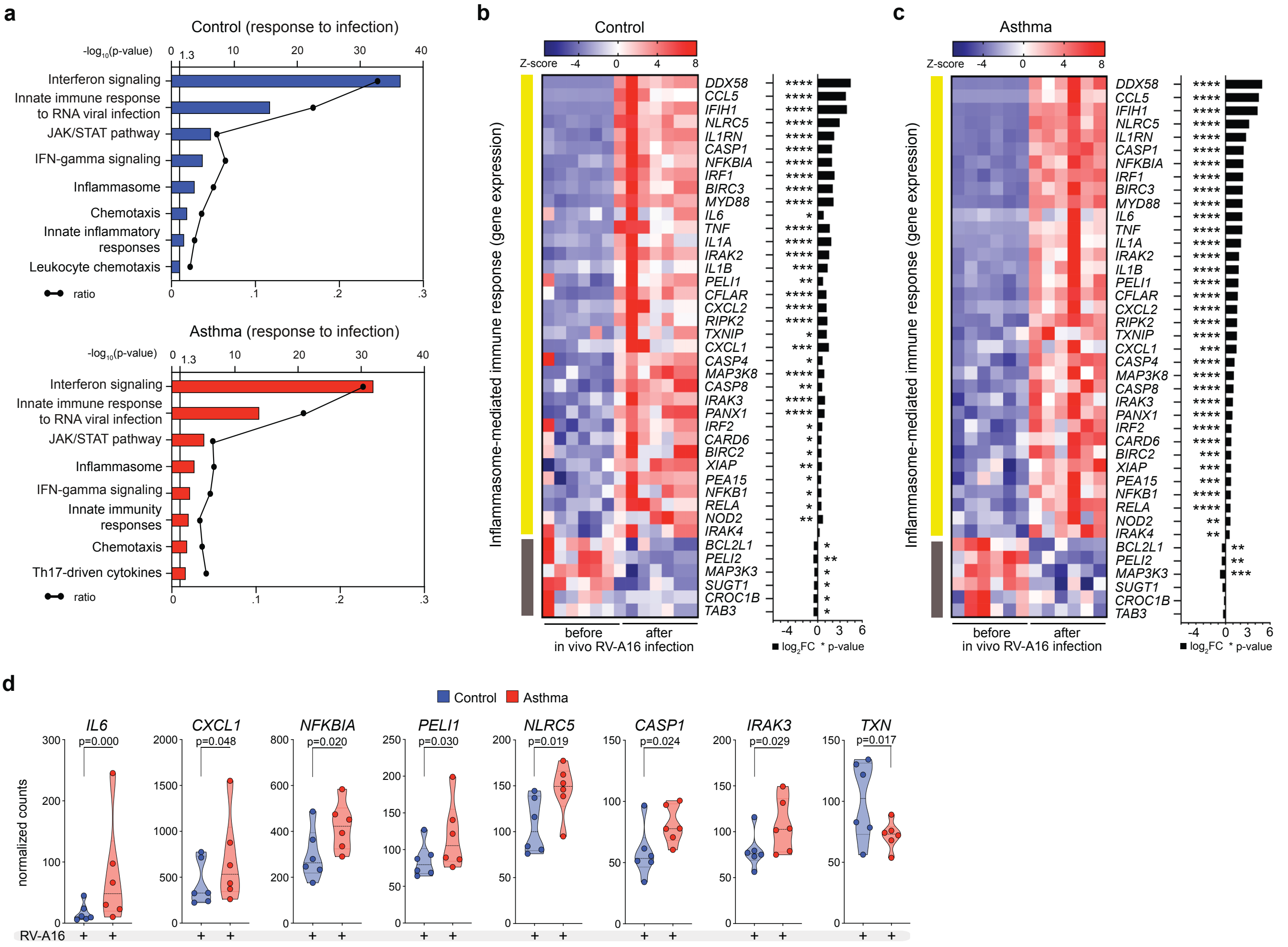

Supplementary Figure 2

Inflammasome- and IL-1β-mediated immune responses are augmented in patients with asthma after infection with rhinovirus

a Top significantly enriched pathways within 100 most significantly upregulated genes in in vitro-cultured HBECs from controls (upper panel) and patients with asthma (lower panel) after in vitro RV-A16 infection (control n=6, asthma n=6). Black line represents a ratio of genes in the experiment over the whole pathway set. **b-c** Heatmap of genes encoding inflammasome-mediated immune responses in in vitro-cultured HBECs from **b** control individuals (n=6) and **c** patients with asthma (n=6) after in vitro RV-A16 infection presented together with the corresponding $\log_2$ fold change (FC). Yellow and grey left side color bars represent genes upregulated or downregulated, respectively. **d** Genes encoding inflammasome-mediated immune responses significantly different between control individuals (n=6) and patients with asthma (n=6) after in vitro RV-A16 infection. HBECs from patients with asthma are presented in red, HBECs from control individuals are presented in blue. Data analyzed from GSE61141. Transcriptome data was analyzed with the edgeR R package[1], raw p-value was presented. Asterisks represent a significant difference as indicated, p-value: *<0.05; **<0.005; ***<0.0005, ****<0.00005. Heatmap displays normalized gene expression across the groups (row normalization). Source data are provided as Source Data files. *HBECs*, differentiated human bronchial epithelial cells; *RV-A16*, rhinovirus A16.

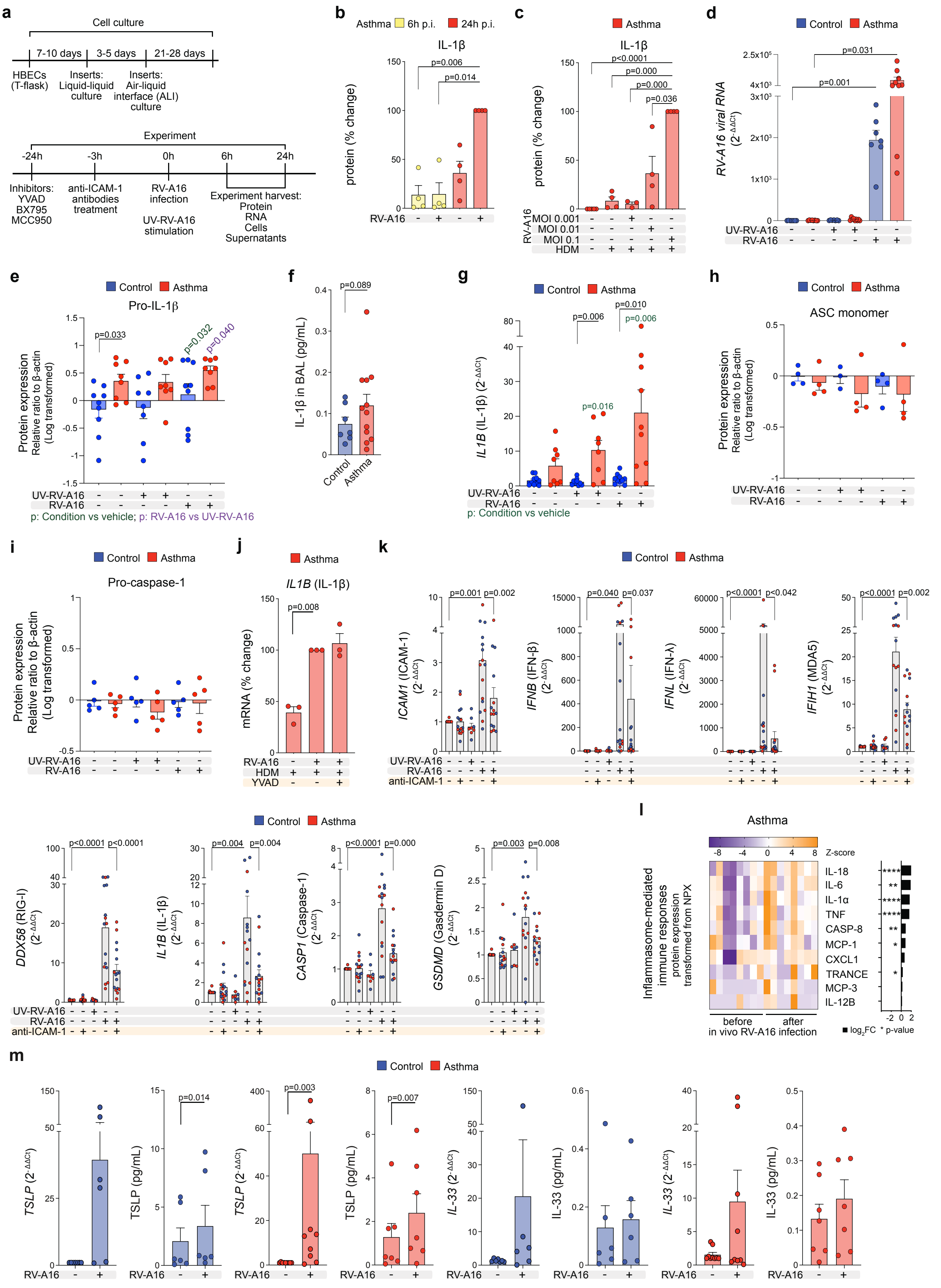
a
Cell culture
7-10 days
3-5 days
21-28 days
HBECs (T-flask)
Inserts: Liquid-liquid culture
Inserts: Air-liquid interface (ALI) culture
Experiment
-24h
-3h
0h
6h
24h
Inhibitors: YVAD BX795 MCC950
anti-ICAM-1 antibodies treatment
RV-A16 infection
UV-RV-A16 stimulation
Experiment harvest: Protein RNA Cells Supernatants
b
Asthma 6h p.i. 24h p.i.
IL-1β
protein (% change)
p=0.006
p=0.014
RV-A16
c
Asthma
IL-1β
protein (% change)
p<0.0001
p=0.000
p=0.000
p=0.036
RV-A16
MOI 0.001
MOI 0.01
MOI 0.1
HDM
d
Control Asthma
RV-A16 viral RNA
p=0.031
p=0.001
UV-RV-A16
RV-A16
e
Control Asthma
Pro-IL-1β
Protein expression Relative ratio to β-actin (Log transformed)
p=0.033
p=0.032
p=0.040
UV-RV-A16
RV-A16
p: Condition vs vehicle; p: RV-A16 vs UV-RV-A16
f
IL-1β in BAL (pg/mL)
p=0.089
Control
Asthma
g
Control Asthma
IL1B (IL-1β)
p=0.006
p=0.010
p=0.006
p=0.016
UV-RV-A16
RV-A16
p: Condition vs vehicle
h
Control Asthma
ASC monomer
Protein expression Relative ratio to β-actin (Log transformed)
UV-RV-A16
RV-A16
i
Control Asthma
Pro-caspase-1
Protein expression Relative ratio to β-actin (Log transformed)
UV-RV-A16
RV-A16
j
Asthma
IL1B (IL-1β)
mRNA (% change)
p=0.008
RV-A16
HDM
YVAD
k
Control Asthma
ICAM1 (ICAM-1)
p=0.001 p=0.002
IFNB (IFN-β)
p=0.040 p=0.037
IFNL (IFN-λ)
p<0.0001 p<0.042
IFIH1 (MDA5)
p<0.0001 p=0.002
UV-RV-A16
RV-A16
anti-ICAM-1
Control Asthma
DDX58 (RIG-I)
p<0.0001 p<0.0001
IL1B (IL-1β)
p=0.004 p=0.004
CASP1 (Caspase-1)
p<0.0001 p=0.000
GSDMD (Gasdermin D)
p=0.003 p=0.008
UV-RV-A16
RV-A16
anti-ICAM-1
l
Asthma
Z-score
Inflammasome-mediated immune responses protein expression transformed from NPX
IL-18
IL-6
IL-1α
TNF
CASP-8
MCP-1
CXCL1
TRANCE
MCP-3
IL-12B
before
after
in vivo RV-A16 infection
log2FC * p-value
m
Control Asthma
TSLP
TSLP (pg/mL)
p=0.014
TSLP
p=0.003
TSLP (pg/mL)
p=0.007
IL-33
IL-33 (pg/mL)
IL-33
IL-33 (pg/mL)
RV-A16

Supplementary Figure 3

Augmented rhinovirus-induced inflammasome activation in airway epithelium from patients with asthma
a Detailed experimental in vitro model overview. Primary human bronchial epithelial cells (HBECs) from control individuals or patients with asthma were differentiated for 21-28 days in the Air-Liquid Interface (ALI) culture. 24h prior in vitro rhinovirus A16 (RV-A16) infection, cells were stimulated in vitro with caspase-1 inhibitor (YVAD), TBK1/IKKε inhibitor (BX795) and NLRP3 inflammasome inhibitor (MCC950) or vehicle. To block cell entry of RV-A16, HBECs were incubated in vitro with anti-ICAM-1 antibodies 3h before the infection. Cells were infected in vitro with RV-A16 at the multiplicity of infection (MOI) 0.1, 0.01 and 0.001 or the respective UV-RV-A16 controls. Protein, RNA, cells, and supernatants were harvested at 6h and 24h after infection (p.i.). **b** IL-1β release to the apical compartment was assessed by ELISA in in vitro-cultured HBECs from patients with asthma for 6h (yellow) and 24h p.i. (red) (n=4). Data are presented as the percentage of the response after 24h p.i. **c** IL-1β release to the apical compartment assessed by ELISA in in vitro-cultured HBECs from patients with asthma 24h after in vitro RV-A16 infection in the MOI 0.1, 0.01, and 0.001, combined with HDM pre-stimulation (n=4; n=3 for HDM+RV-A16 MOI 0.001). Data are presented as the percentage of the response after RV-A16 infection in the MOI 0.1. **d** expression of *RV-A16 positive strand* (RV-A16 viral RNA) was assessed using RT-PCR and presented as the relative quantification ($RQ=2^{-\Delta\Delta Ct}$) as compared to the vehicle condition in in vitro-cultured HBECs from control individuals (n=7) and patients with asthma (n=6, vehicle; n=8, UV-RV-A16, RV-A16). **e** Quantification of densitometry results of pro-IL-1β protein assessed in in vitro-cultured HBECs from control individuals (n=9; n=8, UV-RV-A16) and patients with asthma (n=8) by Western Blot, presented as a log-transformed ratio relative to β-actin and normalized to the vehicle condition in control individuals. **f** IL-1β protein in vivo in the bronchoalveolar lavage (BAL) fluid of control subjects (n=7) and patients with asthma (n=12) (cohort SIBRO), assessed using the mesoscale platform and Welsh`s t-test. **g** mRNA expression of *IL1B* (IL1β) was assessed in in vitro-cultured HBECs from controls (n=10, vehicle, RV-A16; n=9, UV-RV-A16) and patients with asthma (n=8, vehicle, UV-RV-A16; n=10, RV-A16) using RT-PCR and presented as a relative quantification ($RQ=2^{-\Delta\Delta Ct}$) compared to the vehicle from the controls. **h-i** Quantification of densitometry results from **h** ASC and **i** pro-caspase-1 protein expression assessed in in vitro-cultured HBECs from controls (ASC: n=4; n=3 for UV-RV-A16; pro-caspase-1: n=5) and patients with asthma (ASC: n=4; pro-caspase-1: n=5) by Western Blot, presented as a log-transformed ratio relative to β-actin expression and normalized to the vehicle condition separately for each group. **j** mRNA expression of *IL1B* (IL1β) in in vitro-cultured HBECs from patients with asthma (n=3) in the presence or absence of caspase-1 inhibitor (YVAD). Data are presented as the percentage of the response after HDM+RV-A16 treatment. **k** mRNA expression of *ICAM-1*, antiviral and inflammasome-related genes in the presence or absence of anti-ICAM-a antibody in in vitro-cultured HBECs from patients with asthma and healthy controls (n=16; n=8 for UV-RV-A16). **l** Heatmap of proteins associated with the inflammasome-mediated immune responses after in vitro RV-A16 infection in in vitro-cultured HBECs from patients with asthma (n=8) analyzed with the Proximity Extension Assay (PEA) proteomics, transformed from the normalized protein expression (NPX), and presented together with the $\log_2$ fold change (FC) (black bars). Proteins were measured in the apical compartment of the ALI cultures. **m** mRNA and protein expression of TSLP and IL-33 measured with RT-PCR or quantitative PEA in in vitro-cultured HBECs from controls (RT-PCR: n=6, PEA: n=6) and patients with asthma (RT-PCR: n=9, PEA: n=7) and presented as a relative quantification ($RQ=2^{-\Delta\Delta Ct}$) compared to the vehicle or pg/ml, respectively. HBECs from patients with asthma are presented in red, HBECs from control individuals are presented in blue. (n) indicates the number of biologically independent samples examined over at least three independent experiments. Bar graph data show mean ± SEM analyzed with one-way ANOVA (Kruskal-Wallis test), RM one-way ANOVA (Friedman test) or mixed-effects model with post-hoc analysis, as appropriate, depending on the data relation (paired or unpaired) and distribution (if not mentioned differently). Green p-values demonstrate differences between marked conditions and vehicle. Purple p-values demonstrate differences between RV-A16 and UV-RV-A16 conditions. Proximity Extension Assay (PEA) data were analyzed by Bioconductor limma package[2], raw p-value presented. Asterisks represent a significant difference as indicated. *p-value≤0.05, **p-value≤0.01, ***p-value≤0.001, ****p-value≤0.0001. Source data are provided as Source Data files. *anti-ICAM-1*, anti-ICAM-1 antibody; *ALI*; Air-liquid interface cultures*; BAL*; Bronchoalveolar lavage; *BX795*, TBK1/IKKε inhibitor; *HBECs*, differentiated human bronchial epithelial cells; *HDM*, house dust mite; *MCC950*, NLRP3 inflammasome inhibitor; *MOI*, multiplicity of infection; *NPX*, normalized protein expression; *p.i.*, post-infection; *RV-A16*, rhinovirus A16; *UV-RV-A16*, UV-treated rhinovirus A16; *YVAD*, ac-YVAD-cmk (caspase-1 inhibitor).

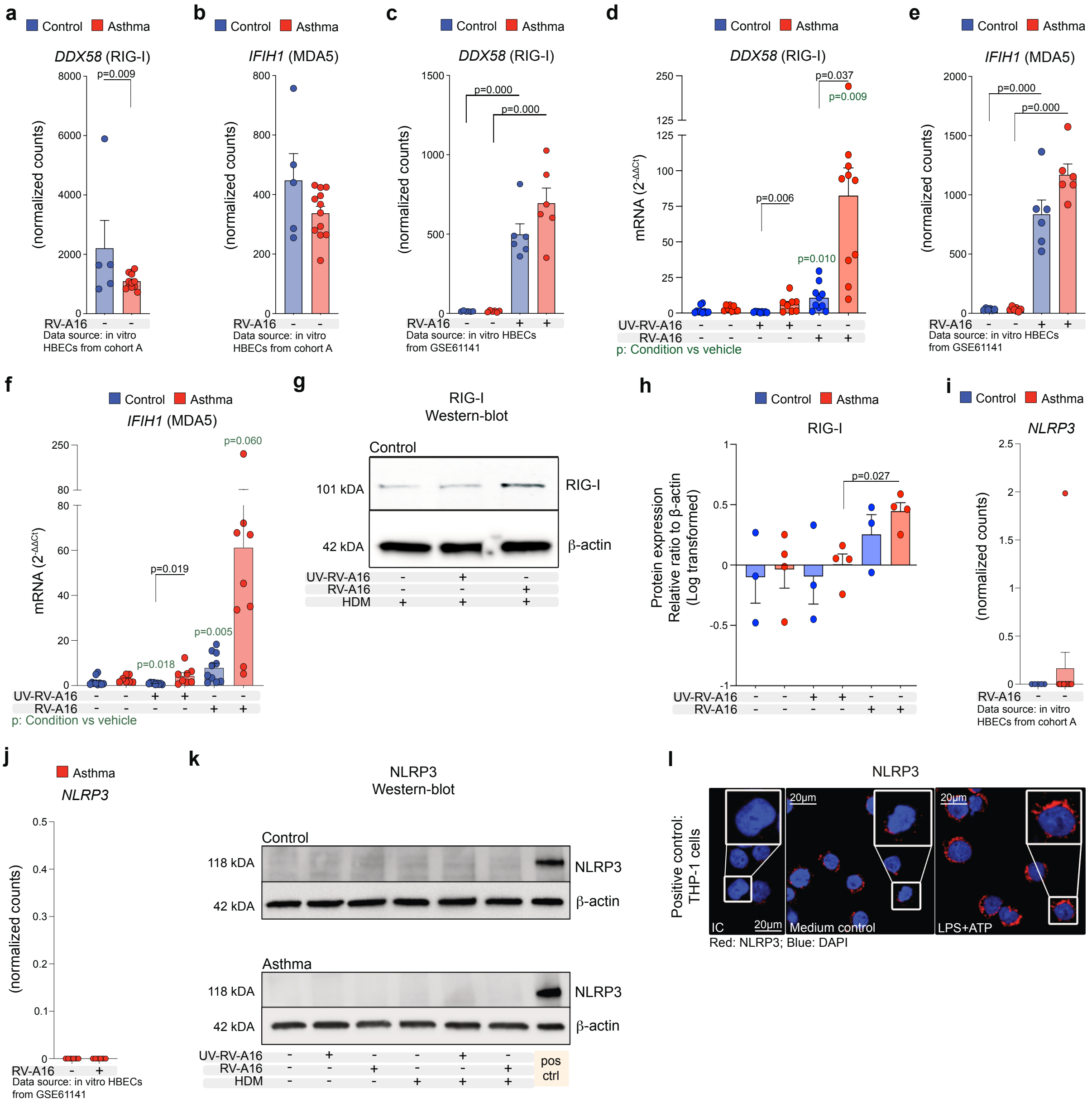

Supplementary Figure 4

Rhinovirus-induced RIG-I, but not NLRP3 inflammasome activation in bronchial epithelium in asthma

a-b Expression of **a** *DDX58* (RIG-I) and **b** *IFIH1* (MDA5) in in vitro-cultured HBECs at baseline (control n=5, asthma n=12). **c** Expression of *DDX58* (RIG-I) in in vitro-cultured HBECs after in vitro RV-A16 infection (control n=6; asthma n=6). **d** mRNA expression of *DDX58* (RIG-I) was assessed in in vitro-cultured HBECs using RT-PCR and presented as a relative quantification (RQ=$2^{-\Delta\Delta Ct}$) as compared to the vehicle condition in HBECs from control subjects (control: n=10, vehicle, RV-A16; n=9, UV-RV-A16; and asthma: n=8, vehicle, UV-RV-A16; n=10, RV-A16). **e** Expression of *IFIH1* (MDA5) in in vitro-cultured HBECs after in vitro RV-A16 infection (control n=6, asthma n=6). **f** mRNA expression of *IFIH1* (MDA5) in in vitro-cultured HBECs assessed using RT-PCR and presented as a relative quantification (RQ=$2^{-\Delta\Delta Ct}$) as compared to the vehicle condition in HBECs from control subjects (control: n=10, vehicle, RV-A16; n=9, UV-RV-A16; and asthma: n=7, vehicle; n=8, UV-RV-A16; n=9, RV-A16). **g** Representative Western Blot images of RIG-I protein expression in in vitro-cultured HBECs from control individuals (n=4). **h** Quantification of densitometry of RIG-I protein expression in in vitro-cultured HBECs from controls (n=3) and patients with asthma (n=4) presented as a log-transformed ratio relative to β-actin and normalized to the vehicle condition in HBECs from control subjects. **i-j** Expression of *NLRP3* in in vitro-cultured HBECs **i** at baseline (control n=5, asthma n=12), and **j** from patients with asthma after in vitro RV-A16 infection (n=6). **k** Representative Western Blot images of NLRP3 protein in in vitro-cultured HBECs from control subjects (n=3, upper panel) and patients with asthma (n=4, lower panel) in all analyzed conditions showed next to the positive control of lipopolysaccharide (LPS)-stimulated monocytes (n=7). **l** Representative confocal images of THP-1 cells used as

positive controls (n=3); scale bars: 20μm. HBECs from patients with asthma are presented in red, HBECs from control individuals are presented in blue. (n) indicates the number of biologically independent samples examined over at least three independent experiments. Bar graph data show mean ± SEM analyzed with one-way ANOVA (Kruskal-Wallis test), RM one-way ANOVA (Friedman test) or mixed-effects model with post-hoc analysis, as appropriate, depending on the data relation (paired or unpaired) and distribution (if not mentioned differently). Green p-values demonstrate differences between marked condition and vehicle. Transcriptome data are presented as normalized counts and analyzed with the edgeR R package[1], raw p-value presented, p: *<0.05; **<0.005; ***<0.0005, ****<0.00005. Data presented on the Supplementary Figure 4 a, b, i: cohort A; Figure 4 c, e, j: GSE61141. Source data are provided as Source Data files. *ATP*, adenosine triphosphate; *HBECs*, differentiated human bronchial epithelial cells; *HDM*, house dust mite; *IC*; Isotype control; *LPS*, lipopolysaccharide; *pos ctrl*, positive control; *RV-A16*, rhinovirus A16; *UV-RV-A16*, UV-treated rhinovirus A16.

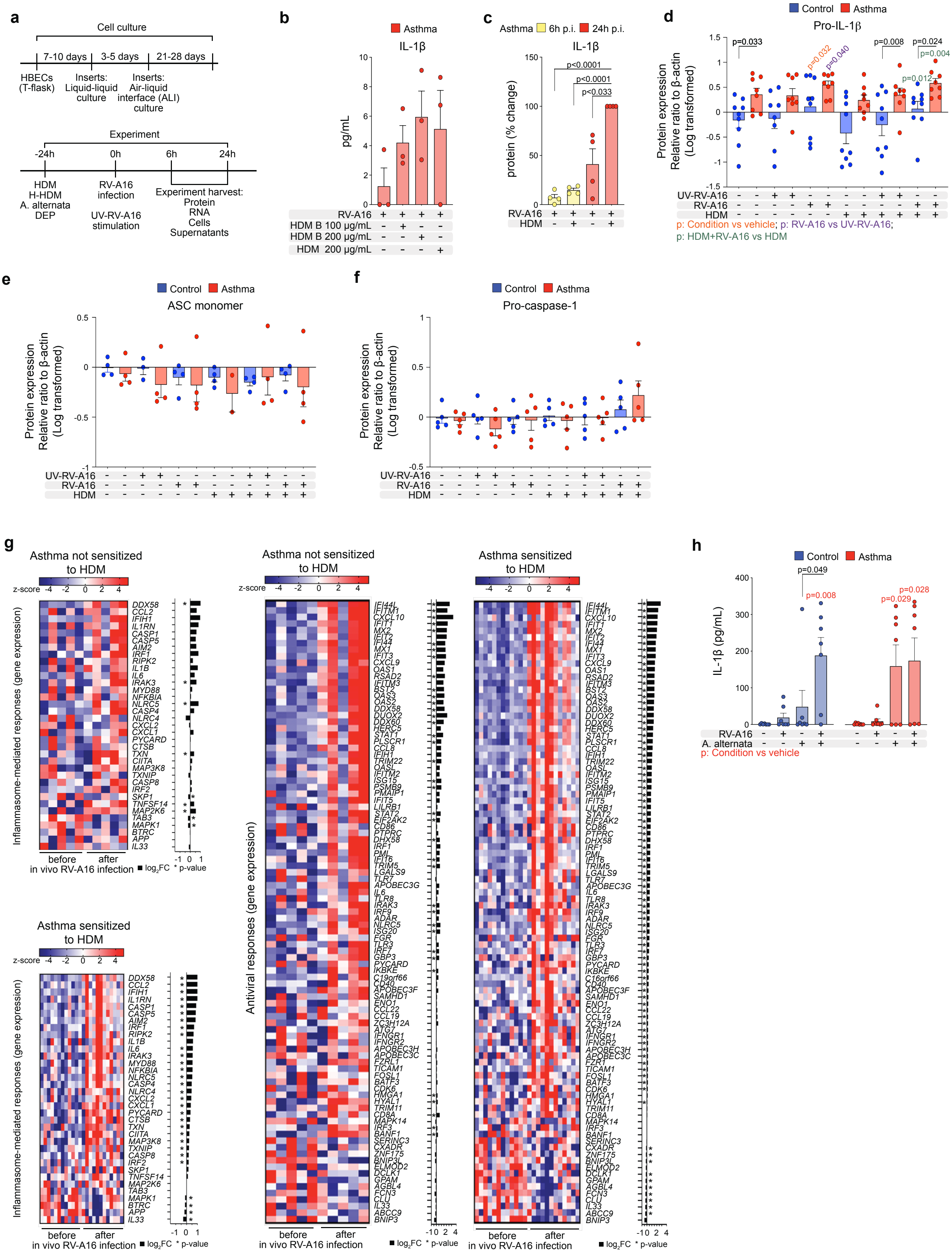

a
Cell culture
7-10 days
3-5 days
21-28 days
HBECs
(T-flask)
Inserts:
Liquid-liquid
culture
Inserts:
Air-liquid
interface (ALI)
culture
Experiment
-24h
0h
6h
24h
HDM
H-HDM
A. alternata
DEP
RV-A16
infection
UV-RV-A16
stimulation
Experiment harvest:
Protein
RNA
Cells
Supernatants
b
Asthma
IL-1β
pg/mL
RV-A16
HDM B 100 µg/mL
HDM B 200 µg/mL
HDM 200 µg/mL
c
Asthma 6h p.i. 24h p.i.
IL-1β
protein (% change)
p<0.0001
p<0.0001
p<0.033
RV-A16
HDM
d
Control Asthma
Pro-IL-1β
Protein expression
Relative ratio to β-actin
(Log transformed)
p=0.033
p=0.032
p=0.040
p=0.008
p=0.024
p=0.004
p=0.012
UV-RV-A16
RV-A16
HDM
p: Condition vs vehicle; p: RV-A16 vs UV-RV-A16;
p: HDM+RV-A16 vs HDM
e
Control Asthma
ASC monomer
Protein expression
Relative ratio to β-actin
(Log transformed)
UV-RV-A16
RV-A16
HDM
f
Control Asthma
Pro-caspase-1
Protein expression
Relative ratio to β-actin
(Log transformed)
UV-RV-A16
RV-A16
HDM
g
Asthma not sensitized
to HDM
z-score
Inflammasome-mediated responses (gene expression)
before
after
in vivo RV-A16 infection
log2FC * p-value
Asthma sensitized
to HDM
Asthma not sensitized
to HDM
Antiviral responses (gene expression)
Asthma sensitized
to HDM
DDX58
CCL2
IFIH1
IL1RN
CASP1
CASP5
AIM2
IRF1
RIPK2
IL1B
IL6
IRAK3
MYD88
NFKBIA
NLRC5
CASP4
NLRC4
CXCL2
CXCL1
PYCARD
CTSB
TXN
CIITA
MAP3K8
TXNIP
CASP8
IRF2
SKP1
TNFSF14
MAP2K6
TAB3
MAPK1
BTRC
APP
IL33
h
Control Asthma
IL-1β (pg/mL)
p=0.049
p=0.008
p=0.029
p=0.028
RV-A16
A. alternata
p: Condition vs vehicle

Supplementary Figure 5

House dust mite enhanced rhinovirus-induced inflammasome activation in bronchial epithelium in asthma
a Detailed experimental in vitro model overview. Primary human bronchial epithelial cells (HBECs) from control individuals or patients with asthma were differentiated for 21-28 days in the Air-Liquid Interface (ALI) culture. 24h prior in vitro rhinovirus A16 (RV-A16) infection, cells were stimulated in vitro with house dust mite (HDM) (200 µg/mL), heat-inactivated HDM (H-HDM) (200 µg/mL), Alternaria alternata (A. alternata) (25µg/mL), diesel exhaust particles (DEP) (25µg/mL), or vehicle. Cells were infected in vitro with RV-A16 at the multiplicity of infection (MOI) 0.1 or the respective UV-RV-A16 controls. Protein, RNA, cells, and supernatants were harvested at 6h and 24h after infection. **b-c** IL-1β release to the apical compartment in in vitro-cultured HBECs was assessed by ELISA **b** for two independent HDM extracts, HDM (the main one used in the manuscript) and HDM B (similar extract from a different company), were tested 24h after in vitro RV-A16 infection (asthma, n=3); and **c** for 6h (yellow) and 24h p.i. (red) (asthma, n=4). Data are presented as the percentage of the response after 24h p.i. **d** Quantification of densitometry results of pro-IL-1β protein assessed by Western Blot in in vitro-cultured HBECs from controls (n=9; n=8, UV-RV-A16), and patients with asthma (n=8) presented as a log-transformed ratio relative to β-actin and normalized to the vehicle condition in control individuals. **e-f** Quantification of densitometry results in in vitro-cultured HBECs of **e** ASC and **f** pro-caspase-1 protein expression assessed by Western Blot, presented as a log-transformed ratio relative to β-actin expression and normalized to the vehicle condition in the HBECs from the control individuals (ASC: control n=4, n=3 for UV-RV-A16; and asthma: n=4, n=2 for HDM; caspase-1: control: n=5; asthma: n=5). d-f no-HDM conditions as in Supplementary Figure 3e, h, i. **g** Heatmap of genes encoding inflammasome-mediated (left) and antiviral (right) immune responses in vivo in bronchial brushings from allergic patients with asthma not sensitized to HDM, but sensitized to six grass pollen mix, cat, dog, Aspergillus fumigatus, Cladosporium herbarium, Alternaria alternata, birch, three tree mix and nettle pollen (n=5) and allergic patients with asthma sensitized to HDM (n=12) and/or six grass pollen mix, cat, dog, Aspergillus fumigatus, Cladosporium herbarium, Alternaria alternata, birch, three tree mix and nettle pollen after in vivo RV-A16 infection presented together with the corresponding $\log_2$ fold change (FC) (Cohort: in vivo RV-A16 infection). **h** IL-1β release to the apical compartment in in vitro-cultured HBECs after A. Alternaria treatment was assessed by ELISA (control n=7, asthma n=7). HBECs from patients with asthma are presented in red, HBECs from control individuals are presented in blue. (n) indicates the number of biologically independent samples examined over at least three independent in vitro experiments. Bar graph data show mean ± SEM analyzed with one-way ANOVA (Kruskal-Wallis test), RM one-way ANOVA (Friedman test) or mixed-effects model with post-hoc analysis, as appropriate, depending on the data relation (paired or unpaired) and distribution. Red p-values demonstrate differences between marked conditions and vehicle. Purple p-values demonstrate differences between RV-A16 and UV-RV-A16 conditions. Green p-values demonstrate differences between HDM+RV-A16 and HDM conditions. Asterisk represents a significant difference as indicated. Transcriptome data analyzed with Bioconductor microarray analysis workflow [https://www.bioconductor.org/packages/release/workflows/vignettes/arrays/inst/doc/arrays.html], raw p-value presented, *p-value≤0.05, **p-value≤0.01, ***p-value≤0.001, ****p-value≤0.0001. Heatmap displays normalized gene expression across the groups (row normalization). Source data are provided as Source Data files. *A. alternata*, Alternaria alternata; *ALI*; Air-liquid interface cultures; *anti-ICAM-1*, anti-ICAM-1 antibody; *DEP*, diesel exhaust particles; *HBECs*, differentiated human bronchial epithelial cells; *HDM*, house dust mite; *H-HDM*, heat-inactivated HDM; *MOI*, multiplicity of infection; *p.i.*, post-infection; *RV-A16*, rhinovirus A16; *UV-RV-A16*, UV-treated rhinovirus A16.

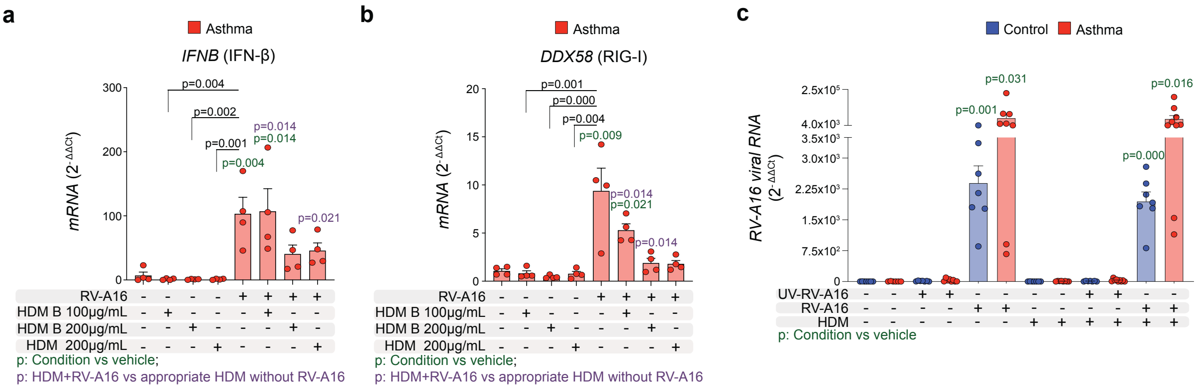

Supplementary Figure 6

House dust mite impaired interferon responses in rhinovirus-infected bronchial epithelium of patients with asthma mRNA expression of **a** *IFNB* (IFN-β) and **b** *DDX58* (RIG-I) in in vitro-cultured HBECs from patients with asthma pre-treated with two HDM extracts from different manufacturers or vehicle for 24h followed by in vitro infection with rhinovirus A16 (RV-A16) in the multiplicity of infection (MOI) 0.1 (n=4), assessed using RT-PCR. **c)** expression of *RV-A16 positive strand* (RV-A16 viral RNA) was assessed in in vitro-cultured HBECs from controls (n=7) and patients with asthma (n=8, HDM; n=9, HDM+UV-RV-A16, HDM+RV-A16) using RT-PCR and presented as the relative quantification (RQ=$2^{-\Delta\Delta Ct}$) as compared to the vehicle condition in the HBECs from control individuals. No-HDM conditions as in Supplementary figure 3d. HBECs from patients with asthma are presented in red, HBECs from control individuals are presented in blue. (n) indicates the number of biologically independent samples examined over at least three independent experiments. Graph data present mean ± SEM analyzed with one-way ANOVA (Kruskal-Wallis test), RM one-way ANOVA (Friedman test) or mixed-effects model with post-hoc analysis, as appropriate, depending on the data relation and distribution. Green p-values demonstrate differences between marked conditions and vehicle. Purple p-values demonstrate differences between HDM+RV-A16 and appropriate HDM without RV-A16 conditions. Source data are provided as Source Data files. *HBECs*, differentiated human bronchial epithelial cells; *HDM*, house dust mite; *MOI*, multiplicity of infection; *RV-A16*, rhinovirus A16; *UV-RV-A16*, UV-treated rhinovirus A16.

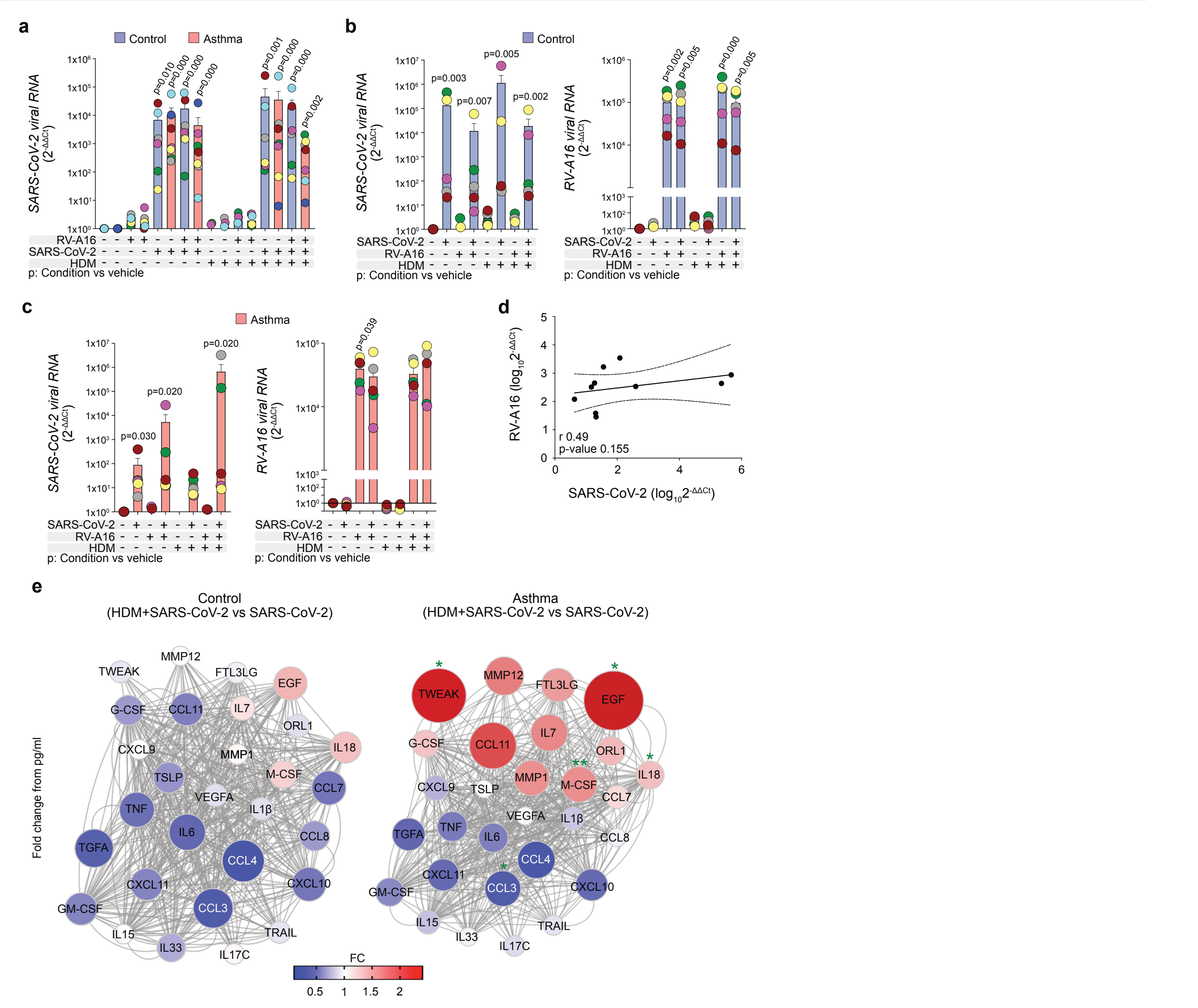

Supplementary Figure 7

Rhinovirus and SARS-CoV-2 co-infection augmented epithelial inflammation after house dust mite exposure in asthma

a SARS-CoV-2 virus load (average expression of *N protein*, *S protein* and *ORF1AB)* in in vitro-cultured HBECs from healthy controls (n=6) and patients with asthma (n=7) was assessed for primary in vitro RV-A16 infection, followed by SARS-CoV-2 co-infection using RT-PCR and presented as relative quantification ($RQ=2^{-\Delta\Delta Ct}$) compared to medium condition for HBECs from controls and patients with asthma separately. **b-d** In vitro-cultured HBECs from control subjects (n=5) and patients with asthma (n=5) were treated in vitro with house dust mite (HDM) (200 µg/mL) or vehicle for 24h, infected in vitro first with SARS-CoV-2 in the multiplicity of infection (MOI) 0.1 for 48h, and then co-infected with RV-A16 in the MOI 0.1 for the following 24h. SARS-CoV-2 virus load (average expression of *N protein*, *S protein* and *ORF1AB*) *and RV-A16 positive strand* (RV-A16 viral RNA in in vitro-cultured HBECS from **b** healthy controls (n=5) and **c** patients with asthma (n=5) were assessed using RT-PCR and presented as relative quantification ($RQ=2^{-\Delta\Delta Ct}$) compared to medium condition separately for HBECs from controls and patients with asthma. **d** Correlation of log_{10}-transformed RV-A16 and SARS-CoV-2 viral loads in in vitro-cultured HBECs in the model of primary SARS-CoV-2 infection followed by secondary RV-A16 co-infection in patients with asthma (n=5) and healthy controls (n=5). **e** Visualization of interaction network of significant proteins secreted to the apical compartment in in vitro-cultured HBECs from control individuals (n=6, left panel) and patients with asthma (n=7, right panel) after in vitro treatment with HDM+SARS-CoV-2, when compared to SARS-CoV-2 infection assessed with quantitative PEA in the approach of first RV-A16 infection, followed by secondary SARS-CoV-2 infection, as presented on Figure 6a. Network nodes represent logFC of significantly

upregulated (red), and downregulated (blue) proteins. Edges represent protein-protein interactions. HBECs from patients with asthma are presented in red, HBECs from control individuals are presented in blue. Green asterisks (*) represent a significant difference between HDM+SARS-CoV-2 vs SARS-CoV-2 conditions. Bar graphs depict the mean ± SEM, whereas color-coded circles show individual data from the same donor (separately for controls and patients with asthma). Data are present as mean analyzed with one-way ANOVA (Kruskal-Wallis test), RM one-way ANOVA (Friedman test) or mixed-effects model with post-hoc analysis, as appropriate, depending on the data relation and distribution, *p-value≤0.05, **p-value≤0.01, ***p-value≤0.001. Black p-values demonstrate differences between marked conditions and vehicle. Correlation between viral loads were calculated with Spearman`s rank correlation test. Source data are provided as Source Data files. *ALI*, air-liquid interface; *HBECs*, Human Bronchial Epithelial Cells; *HDM*, House Dust Mite; *MOI*, multiplicity of infection; *RV-A16*, rhinovirus A16; *SARS-CoV-2*, Severe Acute Respiratory Syndrome Coronavirus 2.

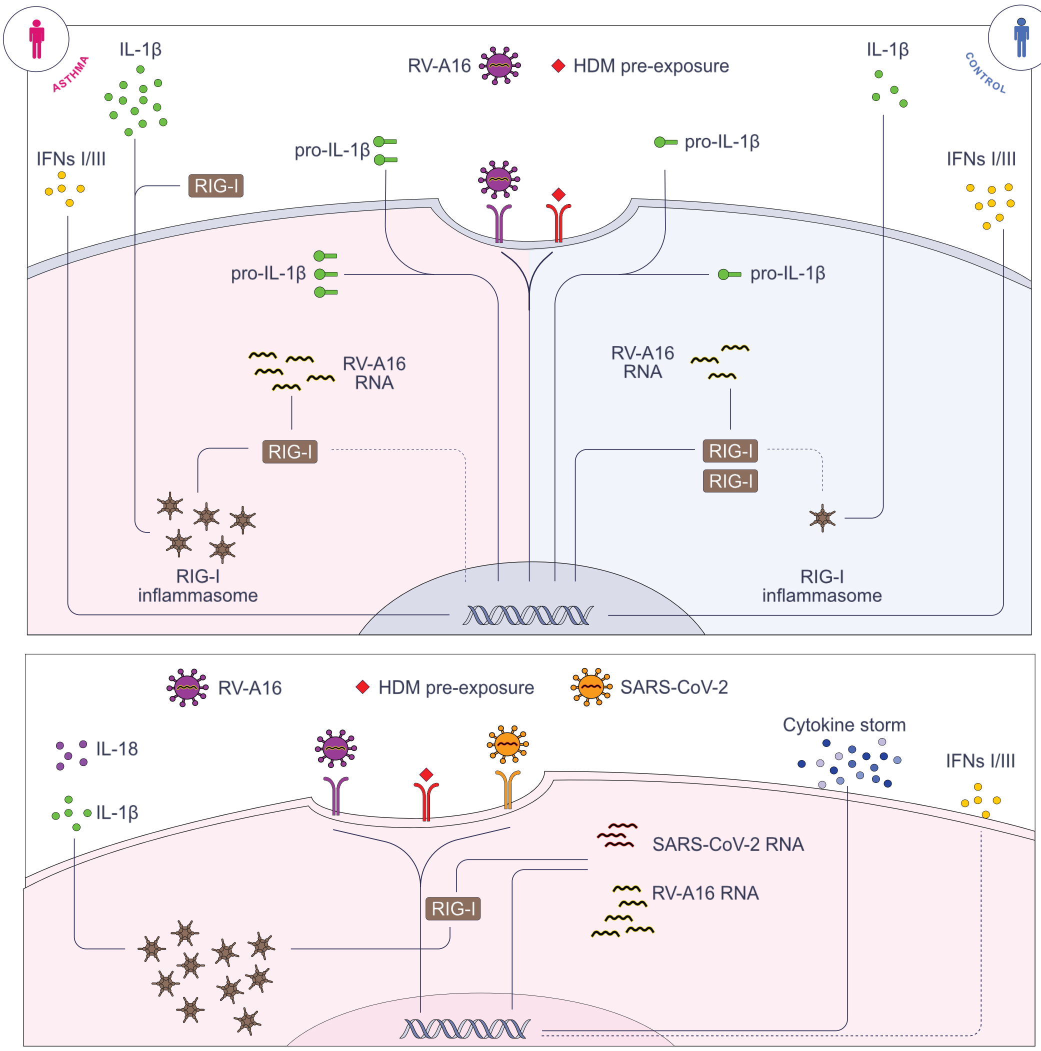

Supplementary Figure 8

The role of epithelial RIG-I signaling in asthma

(Upper panel) Rhinovirus infection in humans is sensed in bronchial airway epithelium via retinoic acid-inducible gene I (RIG-I) helicase. This leads to the recruitment of apoptosis-associated speck like protein containing a caspase recruitment domain (ASC), oligomerization and RIG-I inflammasome activation. Virus-induced RIG-I inflammasome activation- and IL-1β-mediated immune responses are highly augmented in patients with asthma, which is responsible for the functional impairment of the RIG-I-dependent antiviral response, prolonged viral clearance, and unresolved inflammation in asthma. Pre-exposure to house dust mite (HDM) amplifies rhinovirus-induced epithelial injury in patients with asthma via i) enhancement of non-mature pro-IL-1β release, ii) overactivation of RIG-I inflammasome and subsequent release of mature IL-1β, and RIG-I, iii) inhibition of type I/III IFNs and ISG-responses, and iv) activation of extra proinflammatory and pro-remodelling proteins. **(Lower panel)** Pre-existing rhinovirus infection followed by SARS-CoV-2 infection augments RIG-I inflammasome activation and epithelial inflammation in patients with asthma, especially in the presence of HDM.

Supplementary Tables

Supplementary Table 1. Summarized characteristics of experimental in vivo rhinovirus infection study group.

	**Control (9)**	**Asthma (19)**	**p-value**
**Age**<br>Years (mean, SEM)	31 (4.2)	37.05 (2.5)	ns
**Sex**<br>Male, %	66.7%	47.4%	ns
**FeV_1 (%) day 0**<br>% Predicted (mean, SEM)	101.4 (2.3)	83.53 (3.2)	0.0013
**Blood eosinophils**<br>cells/µl (mean, SEM)	0.1125 (0.02)	0.274 (0.06)	0.04
**Total serum IgE**<br>IU/mL (mean, SEM)	17.44 (3.1)	453.7 (136.8)	<0.0001
**Total SPT weal size**<br>mm (mean, SEM)	0.0 (0)	11.89 (1.5)	<0.0001
**Atopy status** (n)	0	19	<0.0001
**Bronchial IL-5 at baseline**<br>pg/ml (mean, SEM)	0.255 (0.14)	1.01 (0.31)	0.047
**Treatment**<br>% of total	na	no ICS – 42%<br>ICS – 58%	ns
**Asthma phenotypes**			
**Severity (GINA)**[3] (n)	na	mild – 6<br>moderate - 13	ns
**Asthma control (ACQ)** (n)	na	well – 7<br>partial -6<br>poor - 6	ns
**Blood eosinophil counts**<br>(cut-off 0.15/µl) (n)	na	low - 7<br>high - 12	ns

FeV_1, forced expiratory volume in 1 second; *ICS*, inhaled corticosteroids; *IgE*, immunoglobulin E; *IU,* arbitrary units; *na*, not applicable; *ns*, not significant; *SEM*, standard error of the mean; *SPT*, skin prick test. Statistical differences were assessed with two-tailed U-Mann Whitney, Fisher`s exact test, or Chi-square test, as appropriate.

Supplementary Table 2. Enrichment analysis of the most significant process networks in bronchial brushings after in vivo rhinovirus A16 (RV-A16) infection in patients with asthma when compared with control individuals.

Genes changed in Asthma after RV-A16 infection (RV-A16 infection vs baseline) vs genes changed in Control after RV-A16 infection (RV-A16 infection vs baseline)					
	**Networks**	**Total**	**p-value**	**In data**	**Network objects from active data**
1	Immune response_Phagosome in antigen presentation	243	6.154E-22	124	NF-kB p50/p50, Profilin I, RhoA, TAP1 (PSF1), I-kB, Beta-2-microglobulin, ITGB1, SHPS-1, PSMD3, PSMD1, PSMD2, ROCK, PSMA1, MHC class II, PI3K cat class IA, Hck, ELMO2, GRP78, Profilin, CD74, TRAM1, PSMB4, Slp76, p38beta (MAPK11), CD14, NCK1, GRB2, NF-kB1 (p105), PSMB6, alpha-M/beta-2 integrin, NF-kB1 (p50), ROCK2, PSMA4, c-Cbl, PSME2, Vinculin, PKC-alpha, MSN (moesin), PI3K cat class IA (p110-beta), ERM proteins, PSMD7, IKK-alpha, VASP, Fc epsilon RI gamma, C3, Lyn, N-WASP, TLR4, Calreticulin, IKK-beta, PSMB2, DOCK1, ROCK1, TAP2 (PSF2), BLNK, FGR, ELMO1, SEC61 beta, PSMB1, MHC class I, PSMB9, HLA-DPA1, PSMD11, Calnexin, PSMB3, alpha-5/beta-1 integrin, WASP, Actin cytoskeletal, PSMF1, HLA-DQB1, JNK(MAPK8-10), PSMD14, IKK (cat), SHP-1, PA28 (11S regulator), PSMB7, PSMD5, VCP, JNK3(MAPK10), iC3b, PSMD12, Fc epsilon RI beta, Legumain, NF-kB, Actin cytoplasmic 2, RDX (radixin), C3dg, FYB1, PSMA7, SEC61 gamma, PSMA3, CD21, HSP90, NF-kB p50/p65, Tapasin, PSMA5, JMJD6, HSP70, LAT, ITGA5, HLA-DQA1, RalA, Sec10, TAP, JNK1(MAPK8), Actin, Derlin1, p38 MAPK, Btk, HSP90 beta, PSMA6, ERp72, HLA-DPB1, PSMA2, NFKBIA, PSME1, FPR, ACTB, Paxillin, Cofilin, Rac1, IP3 receptor, Immunoproteasome (20S core), NF-kB p65/p65
2	Immune response_Antigen presentation	197	3.137E-18	101	STAT3, NF-kB p50/p50, TAP1 (PSF1), IFNGR1, I-kB, Beta-2-microglobulin, JAK2, PSMD3, PSMD1, IP-30, HLA-DRB1, PSMD2, STAT2, PSMA1, CD80, MHC class II, NFYC, GRP78, CD74, CD45, PSMB4, NF-kB1 (p105), PSMB6, alpha-M/beta-2 integrin, NF-kB1 (p50), PSMA4, JAK1, ICAM1, PSME2, PSMD8, PSMD7, IKK-alpha, Fc epsilon RI gamma, TRAP-1, Calreticulin, TRAF6, IKK-beta, PSMB2, IFN-gamma, ECM29, TAP2 (PSF2), SEC61 beta, HLA-DMA, ICAM3, PSMB1, CREB1, MHC class I, PSMB9, HLA-DPA1, PSMD11, Calnexin, PSMB3, HLA-DOA, HLA-F, PSMF1, HLA-DQB1, IFNGR2, PSMD14, IKK (cat), PA28 (11S regulator), IFN-gamma receptor, PSMB7, PSMD5, CD40(TNFRSF5), CEACAM1, PSMD12, Legumain, NF-kB, PSMA7, SEC61 gamma, PSMA3, SP1, HSP90, NF-kB p50/p65, ITGAM, Tapasin, MHC class II beta chain, PSMA5, RFX5, HSP70, TNF-R1, ICOS, HLA-DQA1, KLRK1 (NKG2D), HLA-DMB, ULBP1, LFA-3, TAP, AP-3 beta subunits, HSP90 beta, PSMA6, HLA-DPB1, PSMA2, CD3 delta, CD86, NFKBIA, PSME1, STAT1, Immunoproteasome (20S core), NF-kB p65/p65, TNF-alpha
3	Inflammation_Interferon signaling	110	7.886E-17	66	STAT3, IRF8, IL-1 beta, IFI17, TAP1 (PSF1), IFNGR1, JAK2, MIG, IDO1, MxA, STAT2, MxB, Pyrin (MEFV), IFI27, iNOS, PML, c-Fos, ISG15, JAK1, ICAM1, PKR, CCL2, IFI6, I-TAC, C/EBPbeta, CCL8, ISGF3, ISG54, IL18RAP, ISG20, SSAT, SOCS3, IFN-gamma, TAP2 (PSF2), FasR(CD95), MNDA, IFNAR2, STAT1/STAT2, SERPINB9, IRF7, IRF2, IFP 35, MIP-1-beta, STAT5, IRF9, IFNGR2, IFITM2, SHP-1, IFN-gamma receptor, CD40(TNFRSF5), Apo-2L(TNFSF10), IFN-alpha/beta receptor, IFI44, IRF1, IFNAR1, Caspase-1, Caspase-8, ILT4, TLR3, KLF4, IFI56, CD86, ILT3, TIMP1, STAT1, GBP2
4	Immune response_Phagocytosis	223	1.195E-13	101	NF-kB p50/p50, Profilin I, RhoA, Dectin-1, alpha-X/beta-2 integrin, I-kB, PKC-beta, ITGB1, SHPS-1, ROCK, PI3K cat class IA, IL-15RA, ILT2, Hck, PRK2, RelA (p65 NF-kB subunit), ELMO2, Profilin, PKC-beta1, Slp76, p38beta (MAPK11), CD14, NCK1, GRB2, NF-kB1 (p105), alpha-M/beta-2 integrin, c-Fos, NF-kB1 (p50), ROCK2, ITGB2, c-Cbl, Myosin I, Vinculin, PKC-alpha, p22-phox, MSN (moesin), PI3K cat class IA (p110-beta), ERM proteins, IKK-alpha, VASP, Fc epsilon RI gamma, C3, CORO1A(CLABP, TACO), Lyn, N-WASP, C3b, TLR4, Calreticulin, IKK-beta, MRLC, DOCK1, ROCK1, gp91-phox, c-Jun, BLNK, FGR, ELMO1, C/EBP, Fc gamma RII beta, Myosin II, c-Jun/c-Fos, alpha-5/beta-1 integrin, WASP, Actin cytoskeletal, JNK(MAPK8-10), IKK (cat), SHP-1, PKC-gamma, JNK3(MAPK10), VAV-3, iC3b, Fc epsilon RI beta, NF-kB, Actin cytoplasmic 2, RDX (radixin), C3dg, p40-phox, FYB1, p67-phox, CD21, NF-kB p50/p65, JMJD6, MELC, APOLPA, LAT, ITGA5, ILT4, MyHC, PKC-epsilon, JNK1(MAPK8), Actin, p38 MAPK, Btk, NFKBIA, CD47, ACTB, Paxillin, Cofilin, Rac1, IP3 receptor, NF-kB p65/p65
5	Inflammation_Neutrophil activation	215	7.577E-11	92	Syntaxin 6, STAT3, NF-kB p50/p50, GRO-2, FPRL1, RhoA, I-kB, PKC-beta, TNF-R2, ROCK, PI3K cat class IA, AP-1, G-protein alpha-15, PA2G6, iNOS, p38beta (MAPK11), GRB2, alpha-M/beta-2 integrin, TRAF3, NF-kB1 (p50), ROCK2, ITGB2, JAK1, ICAM1, Cytochrome b-558, MEKK1(MAP3K1), CCL2, PKC-alpha, IL-10, p22-phox, PI3K cat class IA (p110-beta), Apaf-1, IKK-alpha, IL-8, PSGL-1, cPLA2, c-Jun/c-Jun, G-protein alpha-i family, tBid, CX3CR1, H-Ras, Adenylate cyclase, IKK-beta, PAK2, IFN-gamma, Caspase-3, ROCK1, gp91-phox, c-Jun, SODD, NSGPeroxidase, Adenylate cyclase type VI, IL-6, G-protein beta/gamma, Galpha(i)-specific peptide GPCRs, c-Jun/c-Fos, Actin cytoskeletal, Rac2, VTI1B, IKK (cat), PA24A, VAV-3, NF-kB, p40-phox, p67-phox, G-protein alpha-q/11, Syntaxin 1A, NF-kB p50/p65, ITGAM, PLA2, TNF-R1, IL8RA, Caspase-8, SNAP-23, Adenylate cyclase type IX, Actin, GRO-3, p38 MAPK, Btk, G-protein alpha-i2, PKC-beta2, NFKBIA, FPR, ACTB, Cofilin, Rac1, IP3 receptor, ATF-2/c-Jun, IL8RB, NF-kB p65/p65, Bid, TNF-alpha
6	Inflammation_Amphoterin signaling	118	4.657E-10	58	NF-kB p50/p50, RhoA, IL-1 beta, I-kB, ROCK, PI3K cat class IA, AP-1, RelA (p65 NF-kB subunit), p38beta (MAPK11), NF-kB1 (p105), alpha-M/beta-2 integrin, NF-kB1 (p50), ROCK2, ITGB2, Calgranulin A, ICAM1, MyD88, CCL2, PI3K cat class IA (p110-beta), IKK-alpha, Calgranulin B, IL-8, S100B, c-Jun/c-Jun, TLR4, Calgranulin C, H-Ras, TRAF6, IKK-beta, MRLC, ROCK1, c-Jun, IL-6, c-Jun/c-Fos, WASP, Actin cytoskeletal, S100P, JNK(MAPK8-10), IKK (cat), IL1RN, JNK3(MAPK10), NF-kB, SP1, NF-kB p50/p65, ITGAM, MELC, MyHC, JNK1(MAPK8), Actin, p38 MAPK, NFKBIA, ACTB, TLR2, Cofilin, Rac1, NF-kB p65/p65, TNF-alpha, RAGE

7	Cytoskeleton_Regulation of cytoskeleton rearrangement	183	2.707E-09	78	Plectin 1, Tubulin beta, Profilin I, Tubulin gamma, RhoA, RAP-1A, Desmin, 14-3-3 beta/alpha, RhoGDI beta, ROCK, Galpha(i)-specific amine GPCRs, CAPZA, Tubulin beta 1, Tubulin alpha, Cofilin, non-muscle, Profilin, PKC, CAPZ beta, NCK1, ARP3, CD44, PTEN, Vinculin, MSN (moesin), Tubulin gamma 1, ERM proteins, DIA1, Filamin B (TABP), CAPZA1, ACK1, N-WASP, Beta-fodrin, G-protein alpha-i family, DAL1, Zyxin, Nebulin, DOCK2, 14-3-3 zeta/delta, MRLC, G-protein alpha-o, DOCK1, ROCK1, VAV-2, GRAF, Thymosin beta-10, ELMO1, 14-3-3, ARPC1B, Myosin II, SPTBN(spectrin1-4), G-protein beta/gamma, RAP-2A, WASP, Actin cytoskeletal, ARF3, Filamin A, VAV-3, RDX (radixin), Spectrin beta 4, 14-3-3 eta, ARF1, ARPC5, MELC, ARPC1, MyHC, FGD1, PLK1, Actin, ECT2, Tubulin beta 2, ARPC2, ACTB, Paxillin, Cofilin, Actin muscle, Rac1, WaspIP, Tubulin (in microtubules)
8	Inflammation_Innate inflammatory response	180	7.200E-09	76	STAT3, NF-kB p50/p50, COX-2 (PTGS2), IL-1 beta, TLR1, I-kB, DMBT1, PKC-beta, APOBEC3G, G-protein alpha-15, PGRP-S, IRAK4, iNOS, p38beta (MAPK11), CD14, IP10, NF-kB1 (p50), ECSIT, IL-18, MyD88, Beta-defensin 1, MEKK1(MAP3K1), RIPK2, I-TAC, IKK-alpha, C3, IL-8, TLR6, PLAP, Factor I, cPLA2, C3b, G-protein alpha-i family, TLR4, SP-D, TRAF6, IKK-beta, IP3R3, IRAKM, IL1RAP, RIPK1, c-Jun, TBK1, MD-2, IRF7, IL-6, G-protein beta/gamma, SP-C, JNK(MAPK8-10), IKK (cat), IRF3, TANK, PLUNC, JNK3(MAPK10), NF-kB, TAB2, NF-kB p50/p65, Tissue factor, PLA2, TLR3, IP3R2, C3a, C5 convertase (C3b2Bb), JNK1(MAPK8), p38 MAPK, Btk, G-protein alpha-i2, PKC-beta2, NFKBIA, C3aR, sCD14, TLR2, ERAP1, IP3 receptor, NF-kB p65/p65, TNF-alpha
9	Cell adhesion_Leucocyte chemotaxis	180	7.200E-09	76	Tubulin beta, Tubulin gamma, GRO-2, RhoA, RAP-1A, ITGB1, MIG, ROCK, CCL22, CD80, G-protein alpha-12 family, MHC class II, PI3K cat class IA, Tubulin alpha, Profilin, CCL17, Calmodulin, Slp76, CCR7, NCK1, GRB2, IP10, CCL13, GCP2, ROCK2, ITGB2, alpha-4/beta-1 integrin, c-Cbl, ICAM1, PTEN, CCL2, Vinculin, CCL14, PI3K cat class IA (p110-beta), Tubulin gamma 1, I-TAC, VASP, IL-8, N-WASP, G-protein alpha-i family, CX3CR1, DOCK2, CCR2, ROCK1, CXCL13, ARHGEF1 (p115RhoGEF), ICAM3, MIP-1-beta, G-protein beta/gamma, Galpha(i)-specific peptide GPCRs, WASP, Fyn, CCL19, Rac2, CCR1, Galpha(q)-specific peptide GPCRs, FYB1, CCBP2 (CCR9), IL8RA, LAT, ICAM5, SKAP55, LFA-3, GRO-3, Btk, G-protein alpha-i2, CD86, CXCR4, Paxillin, Cofilin, Rac1, IP3 receptor, CCL15, G-protein alpha-13, IL8RB, Tubulin (in microtubules)
10	Immune response_Innate immune response to RNA viral infection	84	1.808E-08	43	I-kB, IDO1, MxA, STAT2, WARS, IRAK4, iNOS, p38beta (MAPK11), IP10, TRAF3, JAK1, MyD88, PKR, MDA-5, I-TAC, IL-8, ISGF3, RNaseL, ADAR1, TRAF6, IKK-beta, IRAKM, RIPK1, c-Jun, TBK1, IRF7, IL-6, IRF9, IKK (cat), IRF3, TANK, TLR7, TLR8, eIF2S1, NF-kB, TAB2, IFN-alpha/beta receptor, IRF1, Caspase-8, TLR3, 2'-5'-oligoadenylate synthetase, STAT1, RLI
54	Inflammation_Inflammasome	118	2.680E-03	41	IL-1 beta, Caspase-5, I-kB, AP-1, IRAK4, p38beta (MAPK11), NF-kB1 (p105), ISG15, IL-18, MyD88, PKR, MDA-5, RIPK2, IKK-alpha, ISGF3, CARD5, TLR4, TRAF6, IKK-beta, RIPK1, TBK1, IRF7, Nod2 (CARD15), JNK(MAPK8-10), IKK (cat), IRF3, TLR7, TLR8, eIF2S1, NF-kB, TAB2, Pannexin-1, TNF-R1, Caspase-1, TLR3, p38 MAPK, Btk, eIF2S2, NFKBIA, CARD8, TNF-alpha

Data analyzed with enrichment tool using Metacore software, raw p-value presented.

Supplementary Table 3. Enrichment analysis of the ten most significant process networks in the top one hundred upregulated genes in in vitro-cultured human bronchial epithelial cells (HBECs) from control individuals and patients with asthma after in vitro rhinovirus A16 infection. Analyzed from GSE61141[4].

HBECs from control individuals					
	**Networks**	**Total**	**p-value**	**In data**	**Network objects from active data**
1	Inflammation_Interferon signaling	110	3.409E-37	27	IL29, PKR, CCL5, IFI17, IRF1, IL28A, MxB, ISG20, TAP1 (PSF1), IFI44, GBP1, I-TAC, Apo-2L(TNFSF10), STAT1/STAT2, SOCS1, IDO1, ISG54, STAT1, IRF7, MxA, IFP 35, IFI56, ISG15, IL28B, TLR3, PML, STAT2
2	Immune response_Innate immune response to RNA viral infection	83	1.840E-16	14	PKR, MDA-5, RIG-I, IP10, IRF1, I-TAC, WARS, 2'-5'-oligoadenylate synthetase, IDO1, STAT1, IRF7, MxA, TLR3, STAT2
3	Inflammation_Jak-STAT Pathway	185	4.596E-07	10	IL29, CCL5, IL28A, PLAUR (uPAR), LIFR, STAT1/STAT2, SOCS1, STAT1, IL28B, STAT2
4	Inflammation_IFN-gamma signaling	109	9.450E-06	7	PKR, CCL5, K12, IP10, IRF1, SOCS1, STAT1
5	Inflammation_Inflammasome	120	1.756E-04	6	PKR, MDA-5, RIG-I, IRF7, ISG15, TLR3
6	Chemotaxis	139	2.765E-03	5	CCL5, IP10, CX3CL1, PLAUR (uPAR), I-TAC
7	Inflammation_Innate inflammatory response	181	8.446E-03	5	APOBEC3G, IP10, I-TAC, IRF7, TLR3
8	Cell adhesion_Leucocyte chemotaxis	180	3.694E-02	4	CCL5, IP10, CX3CL1, I-TAC
9	Proliferation_Negative regulation of cell proliferation	183	3.889E-02	4	PKR, IFI17, WARS, STAT1
10	Immune response_Antigen presentation	194	4.655E-02	4	TAP1 (PSF1), STAT1, CEACAM1, STAT2

HBECs from patients with asthma					
	**Networks**	**Total**	**p-value**	**In data**	**Network objects from active data**
1	Inflammation_Interferon signaling	110	1.008E-32	25	IL29, CCL5, IFI17, IL28A, MxB, ISG20, TAP1 (PSF1), IFI44, GBP1, I-TAC, Caspase-1, Apo-2L(TNFSF10), MIG, STAT1/STAT2, IDO1, ISG54, STAT1, IRF7, MxA, IFP 35, IFI56, IL28B, TLR3, PML, STAT2
2	Immune response_Innate immune response to RNA viral infection	83	1.227E-14	13	MDA-5, RIG-I, IP10, IKK-epsilon, I-TAC, WARS, 2'-5'-oligoadenylate synthetase, IDO1, STAT1, IRF7, MxA, TLR3, STAT2
3	Inflammation_Jak-STAT Pathway	185	6.247E-06	9	IL29, IL-15RA, CCL5, IL28A, LIFR, STAT1/STAT2, STAT1, IL28B, STAT2
4	Inflammation_Inflammasome	120	2.189E-04	6	MDA-5, RIG-I, IKK-epsilon, Caspase-1, IRF7, TLR3
5	Inflammation_IFN-gamma signaling	109	1.126E-03	5	CCL5, K12, IP10, MIG, STAT1
6	Inflammation_Innate inflammatory response	181	1.918E-03	6	APOBEC3G, IP10, IKK-epsilon, I-TAC, IRF7, TLR3
7	Chemotaxis	139	3.287E-03	5	CCL5, IP10, I-TAC, IL-16, MIG
8	Immune response_Th17-derived cytokines	98	5.518E-03	4	MMP-13, I-TAC, MIG, STAT1
9	Inflammation_NK cell cytotoxicity	163	3.063E-02	4	IL-15RA, Apo-2L(TNFSF10), STAT1, STAT2
10	Cell adhesion_Platelet-endothelium-leucocyte interactions	174	3.063E-02	4	MMP-13, CCL5, STAT1, CD68

Data analyzed with enrichment tool using Metacore software, raw p-value presented.

Supplementary Table 4. All antiviral response genes significantly changed after in vivo rhinovirus A16 (RV-A16) infection in bronchial brushings from control individuals and patients with asthma.

Bronchial brushings from control individuals in vivo		
**Gene**	**p-value**	**Log_2FC**
LYST	7.65E-05	-0.443907
IVNS1ABP	1.08E-04	-0.4197461
MAPK11	3.59E-04	0.28467409
BECN1	4.00E-04	-0.3110455
CCT5	4.43E-04	-0.412418
TBK1	4.93E-04	-0.5302973
ACTA2	5.67E-04	-0.4198554
ELMOD2	6.05E-04	-0.4646418
ABCE1	7.70E-04	-0.4356744
CD207	0.00120396	0.46706636
DNAJC3	0.00121386	-0.38085
BNIP3	0.00162649	-0.4523524
UNC93B1	0.00165717	0.29162879
SPACA3	0.00186858	0.29907682
IKBKB	0.00253497	0.31654719
IL33	0.00257272	-0.5662286
IFNAR1	0.00277058	-0.3379496
SLFN11	0.00304737	-0.5547823
IRF3	0.00309228	0.26590699
APOBEC3C	0.00450924	-0.4267123
CHRM2	0.00499786	0.2406417
IFNAR2	0.00502243	-0.3416906
CXADR	0.00512015	-0.2713458
MAPK14	0.00614791	-0.2494267
HSPB1	0.00753452	-0.2903693
CXCR4	0.00848183	-0.9033424
KCNJ8	0.01022031	0.19106938
CCDC130	0.01152673	0.26813823
POLR3G	0.01328435	0.29856694
DDX1	0.01405911	-0.2643058
RELA	0.01479098	-0.2935714
TRIM56	0.01547338	0.1881259
TLR8	0.01552932	-0.7104081
PSMA2	0.01595628	-0.4307298
CHUK	0.01748839	-0.3588789
FCN3	0.0184118	0.27715324
IL12B	0.0188253	0.208578
POLR3F	0.02210039	-0.2253792
HYAL2	0.02246593	0.1740213
CFL1	0.02444936	-0.3017413
IFNGR2	0.02559222	-0.3396424
UNC93B1	0.02568665	0.28795446
FGR	0.02652297	-0.7025369
DDX21	0.02830701	-0.3674024
RNASEL	0.02979535	-0.1934832
PTPRC	0.03030047	-0.8586604
DHX36	0.03121596	-0.3011299
TNF	0.0315715	-0.5356796
TPT1	0.0326018	0.45659592
DDX3X	0.033224	-0.2460331
XCL1	0.03392249	0.68704931
MICA	0.03469049	0.24172981
BANF1	0.03533612	0.21109478
MEF2C	0.03647154	-0.4306598
CD86	0.03691275	-0.8744841
CYP1A1	0.03713228	0.25314154
BANF1	0.03814064	-0.2129742
CCL4	0.04035564	-0.703145
CDK6	0.04161901	-0.1921755
PRKRA	0.04265319	-0.1935144
IL6	0.044015	-0.6248495
BNIP3L	0.0445931	-0.2107504
GBP3	0.0463936	-0.4441241

Bronchial brushings from patients with asthma in vivo		
**Gene**	**p-value**	**Log_2FC**
IFI44L	2.7513864	2.44E-07
IFITM1	2.26654724	8.93E-07

IFI44	1.78485617	1.86E-06
IFITM3	1.49792001	2.05E-06
BNIP3	-0.4879504	2.91E-06
MX1	1.79778192	6.72E-06
BST2	1.5213133	7.38E-06
IRF9	0.5769468	8.53E-06
OAS2	1.44133293	9.67E-06
STAT1	1.26202938	1.22E-05
OAS1	1.54613253	1.25E-05
MX2	2.04970628	1.52E-05
IFIT1	2.16478729	1.85E-05
PLSCR1	1.12390665	2.00E-05
DDX60	1.41443322	2.87E-05
TRIM22	1.02773215	3.26E-05
OAS3	1.54432653	5.23E-05
IFIT3	1.81597424	5.49E-05
DHX58	0.70446222	9.76E-05
DDX58	1.47047202	9.79E-05
EIF2AK	0.7781584	1.71E-04
FCN3	-0.3050202	1.99E-04
IKBKE	0.36925583	2.21E-04
STAT2	0.82594415	2.36E-04
GPAM	-0.2662866	2.96E-04
IFI16	0.66718059	3.25E-04
ISG15	0.96431722	3.28E-04
IFIT2	1.98331393	3.34E-04
DUOX2	1.62111069	3.40E-04
CXCL10	2.51578782	3.53E-04
PSMB9	0.94885053	3.74E-04
ISG20	0.59236245	4.44E-04
ADAR	0.52414375	4.48E-04
RSAD2	1.58864248	4.60E-04
NLRC5	0.61071381	6.45E-04
TRIM5	0.68854983	6.60E-04
IFITM2	0.87811185	7.01E-04
IRF7	0.47976395	7.04E-04
IRAK3	0.5721565	8.88E-04
HERC5	1.15893187	0.00107361
PML	0.66965248	0.00129139
CLU	-0.2599909	0.00156214
IFIT5	0.78368937	0.0017862
IFIH1	1.05426744	0.00181852
CXCL9	1.63985158	0.00260229
IRF1	0.72633033	0.00279675
PMAIP1	0.78214465	0.003265
GBP3	0.43999573	0.00331372
PYCARD	0.26990835	0.00334091
ZNF175	-0.2148014	0.00442761
CCL22	0.23745083	0.00563918
CCL19	0.24199143	0.0057422
APOBEC3G	0.59811982	0.00594614
ZC3H12A	0.27829253	0.00721255
OASL	0.89995485	0.00761047
APOBEC3F	0.34347153	0.0082971
IL6	0.53328796	0.00912099
ENO1	0.23331382	0.00934609
ABCC9	-0.2962732	0.00937097
BATF3	0.20857068	0.00957246
FOSL1	0.17740254	0.00977377
LGALS9	0.49906318	0.009962
HYAL1	0.15341526	0.0102313
APOBEC3H	0.21874676	0.01147115
TLR3	0.47466227	0.01234421
SERINC3	-0.1090338	0.01379487
SAMHD1	0.25655864	0.0140242
TLR8	0.46085583	0.01454437
PTPRC	0.62254589	0.01553325
TRIM11	0.10106083	0.01588222
CCL8	1.08815918	0.01736971
BNIP3L	-0.1621839	0.01771446
DCLK1	-0.1757575	0.01996567
LILRB1	0.63895374	0.02009985
CXADR	-0.1408438	0.02034223
F2RL1	0.20734859	0.02125084
TICAM1	0.18010247	0.02131712

APOBEC3D	0.22884253	0.02143983
IL33	-0.2644045	0.02260751
MST1R	0.19118748	0.02307895
HMGA1	0.13802169	0.02337107
ATG7	0.19313255	0.02563914
CDK6	0.13288052	0.02909091
CD86	0.5879905	0.02938831
HNRNPUL1	-0.1108471	0.03039986
CD207	-0.188618	0.03084029
IFNG	0.62195628	0.03202974
AGBL4	-0.2163882	0.03219032
IFNE	0.16956428	0.03676611
PRF1	0.4861052	0.04035228
CD40	0.30119882	0.0404023
ELMOD2	-0.1612959	0.04506776
SRC	0.13429267	0.04968988

Data analyzed by Bioconductor microarray analysis workflow [https://www.bioconductor.org/packages/release/workflows/vignettes/arrays/inst/doc/arrays.html], raw p-value presented.

Supplementary Table 5. KEGG pathways enriched in significantly changed proteins secreted from the in vitro-cultured human bronchial epithelial cells of control subjects and patients with asthma after in vitro house dust mite stimulation and rhinovirus A16 (RV-A16) infection, as compared to RV-A16 infection alone.

KEGG pathways downregulated (controls)						
#term ID	term description	observed gene count	background gene count	strength	false discovery rate	matching proteins in your network (labels)
map04060	Cytokine-cytokine receptor interaction	21	143	0.79	0.0000000000139	CX3CL1, TGFB1, CCL2, CXCL6, KITLG, IL12B, TNFSF10, IL18, CXCL5, CXCL11, CCL20, CCL28, CCL7, CCL8, IL6, TNF, TNFSF14, FLT3LG, CCL3, VEGFA, CCL4
map04062	Chemokine signaling pathway	11	40	1.06	0.000000202	CX3CL1, CCL2, CXCL6, CXCL5, CXCL11, CCL20, CCL28, CCL7, CCL8, CCL3, CCL4
map05323	Rheumatoid arthritis	10	36	1.06	0.000000784	TGFB1, CCL2, CXCL6, IL18, CXCL5, CCL20, IL6, TNF, CCL3, VEGFA
map04620	Toll-like receptor signaling pathway	7	22	1.12	0.0000553	IL12B, CXCL11, CASP8, IL6, TNF, CCL3, CCL4
map04657	IL-17 signaling pathway	8	36	0.97	0.0000725	CCL2, CXCL6, CXCL5, CASP8, CCL20, CCL7, IL6, TNF
map05142	Chagas disease (American trypanosomiasis)	7	28	1.02	0.00014	TGFB1, CCL2, IL12B, CASP8, IL6, TNF, CCL3
map04668	TNF signaling pathway	7	31	0.97	0.00022	CX3CL1, CCL2, CXCL5, CASP8, CCL20, IL6, TNF
map05134	Legionellosis	5	12	1.24	0.00036	IL12B, IL18, CASP8, IL6, TNF
map05200	Pathways in cancer	10	98	0.63	0.001	TGFB1, KITLG, IL12B, AXIN1, TGFA, FGF5, CASP8, IL6, FLT3LG, VEGFA
map05168	Herpes simplex infection	6	31	0.91	0.0015	CCL2, IL12B, CASP8, IL6, TNF, TNFSF14
map05164	Influenza A	6	33	0.88	0.0019	CCL2, IL12B, TNFSF10, IL18, IL6, TNF
map04621	NOD-like receptor signaling pathway	5	21	1	0.0022	CCL2, IL18, CASP8, IL6, TNF
map05133	Pertussis	5	21	1	0.0022	CXCL6, IL12B, CXCL5, IL6, TNF
map05152	Tuberculosis	6	36	0.84	0.0023	TGFB1, IL12B, IL18, CASP8, IL6, TNF
map05321	Inflammatory bowel disease (IBD)	5	23	0.96	0.0025	TGFB1, IL12B, IL18, IL6, TNF
map04933	AGE-RAGE signaling pathway in diabetic complications	5	25	0.92	0.0033	TGFB1, CCL2, IL6, TNF, VEGFA
map05144	Malaria	5	26	0.9	0.0037	TGFB1, CCL2, IL18, IL6, TNF
map05132	Salmonella infection	4	14	1.08	0.0038	IL18, IL6, CCL3, CCL4
map05143	African trypanosomiasis	4	15	1.05	0.0044	IL12B, IL18, IL6, TNF
map04010	MAPK signaling pathway	7	71	0.61	0.0086	TGFB1, KITLG, TGFA, FGF5, TNF, FLT3LG, VEGFA
map04932	Non-alcoholic fatty liver disease (NAFLD)	4	19	0.94	0.0086	TGFB1, CASP8, IL6, TNF
map05161	Hepatitis B	4	20	0.92	0.0097	TGFB1, CASP8, IL6, TNF
map04623	Cytosolic DNA-sensing pathway	3	9	1.14	0.0109	IL18, IL6, CCL4
map05146	Amoebiasis	4	22	0.88	0.0121	TGFB1, IL12B, IL6, TNF
map05145	Toxoplasmosis	4	24	0.84	0.0154	TGFB1, IL12B, CASP8, TNF
map04622	RIG-I-like receptor signaling pathway	3	11	1.06	0.0155	IL12B, CASP8, TNF
map05212	Pancreatic cancer	3	11	1.06	0.0155	TGFB1, TGFA, VEGFA
map01521	EGF5R tyrosine kinas5e inhibitor resistance	4	26	0.81	0.0177	TGFA, EIF4EBP1, IL6, VEGFA
map05211	Renal cell carcinoma	3	12	1.02	0.0177	TGFB1, TGFA, VEGFA
map04014	Ras signaling pathway	5	45	0.67	0.0189	KITLG, TGFA, FGF5, FLT3LG, VEGFA

map05165	Human papillomavirus infection	5	46	0.66	0.02	AXIN1, EIF4EBP1, CASP8, TNF, VEGFA
map04151	PI3K-Akt signaling pathway	7	92	0.5	0.0215	KITLG, TGFA, FGF5, EIF4EBP1, IL6, FLT3LG, VEGFA
map05210	Colorectal cancer	3	14	0.95	0.0218	TGFB1, AXIN1, TGFA
map05225	Hepatocellular carcinoma	3	14	0.95	0.0218	TGFB1, AXIN1, TGFA
map05410	Hypertrophic cardiomyopathy (HCM)	3	14	0.95	0.0218	TGFB1, IL6, TNF
map04068	FoxO signaling pathway	3	17	0.87	0.0319	TGFB1, TNFSF10, IL6
map04218	Cellular senescence	3	19	0.82	0.0407	TGFB1, EIF4EBP1, IL6
map01523	Antifolate resistance	2	6	1.14	0.0408	IL6, TNF
map04672	Intestinal immune network for IgA production	3	21	0.77	0.0492	TGFB1, CCL28, IL6
map05140	Leishmaniasis	3	21	0.77	0.0492	TGFB1, IL12B, TNF
map05226	Gastric cancer	3	21	0.77	0.0492	TGFB1, AXIN1, FGF5

Pathways marked in red demonstrate proteins included in viral infection pathway on the Figure 5b

**KEGG pathways upregulated (controls)**
No significant enrichment

**KEGG pathways downregulated (asthma)**						
**#term ID**	**term description**	**observed gene count**	**background gene count**	**strength**	**false discovery rate**	**matching proteins in your network (labels)**
map04060	Cytokine-cytokine receptor interaction	21	143	0.79	0.0000000000132	CX3CL1, TGFB1, CCL2, CXCL6, KITLG, TNFSF10, IL17C, IL7, CXCL5, CXCL10, CXCL11, CCL20, CCL28, CCL7, IL6, TNF, TNFSF14, FLT3LG, CCL3, VEGFA, CCL4
map04062	Chemokine signaling pathway	11	40	1.06	0.000000192	CX3CL1, CCL2, CXCL6, CXCL5, CXCL10, CXCL11, CCL20, CCL28, CCL7, CCL3, CCL4
map04657	IL-17 signaling pathway	10	36	1.06	0.000000742	CCL2, CXCL6, IL17C, CXCL5, CXCL10, MMP1, CCL20, CCL7, IL6, TNF
map05323	Rheumatoid arthritis	10	36	1.06	0.000000742	TGFB1, CCL2, CXCL6, CXCL5, MMP1, CCL20, IL6, TNF, CCL3, VEGFA
map04668	TNF signaling pathway	7	31	0.97	0.00029	CX3CL1, CCL2, CXCL5, CXCL10, CCL20, IL6, TNF
map04620	Toll-like receptor signaling pathway	6	22	1.06	0.00045	CXCL10, CXCL11, IL6, TNF, CCL3, CCL4
map05200	Pathways in cancer	10	98	0.63	0.0012	TGFB1, KITLG, AXIN1, IL7, TGFA, FGF5, MMP1, IL6, FLT3LG, VEGFA
map04933	AGE-RAGE signaling pathway in diabetic complications	5	25	0.92	0.0063	TGFB1, CCL2, IL6, TNF, VEGFA
map05142	Chagas disease (American trypanosomiasis)	5	28	0.87	0.0088	TGFB1, CCL2, IL6, TNF, CCL3
map04010	MAPK signaling pathway	7	71	0.61	0.015	TGFB1, KITLG, TGFA, FGF5, TNF, FLT3LG, VEGFA
map04151	PI3K-Akt signaling pathway	8	92	0.56	0.015	KITLG, IL7, TGFA, FGF5, EIF4EBP1, IL6, FLT3LG, VEGFA
map05164	Influenza A	5	33	0.8	0.015	CCL2, TNFSF10, CXCL10, IL6, TNF
map04623	Cytosolic DNA-sensing pathway	3	9	1.14	0.0181	CXCL10, IL6, CCL4
map05133	Pertussis	4	21	0.9	0.0181	CXCL6, CXCL5, IL6, TNF
map04640	Hematopoietic cell lineage	5	41	0.71	0.0248	KITLG, IL7, IL6, TNF, FLT3LG
map05212	Pancreatic cancer	3	11	1.06	0.0248	TGFB1, TGFA, VEGFA
map01521	EGFR tyrosine kinase inhibitor resistance	4	26	0.81	0.0277	TGFA, EIF4EBP1, IL6, VEGFA
map04014	Ras signaling pathway	5	45	0.67	0.0277	KITLG, TGFA, FGF5, FLT3LG, VEGFA

map04926	Relaxin signaling pathway	3	13	0.98	0.0277	TGFB1, MMP1, VEGFA
map05144	Malaria	4	26	0.81	0.0277	TGFB1, CCL2, IL6, TNF
map05211	Renal cell carcinoma	3	12	1.02	0.0277	TGFB1, CCL2, IL6, TNF
map05132	Salmonella infection	3	14	0.95	0.031	IL6, CCL3, CCL4
map05210	Colorectal cancer	3	14	0.95	0.031	TGFB1, AXIN1, TGFA
map05225	Hepatocellular carcinoma	3	14	0.95	0.031	TGFB1, AXIN1, TGFA
map05410	Hypertrophic cardiomyopathy (HCM)	3	14	0.95	0.031	TGFB1, IL6, TNF
map05168	Herpes simplex infection	4	31	0.73	0.0319	CCL2, IL6, TNF, TNFSF14
map04068	FoxO signaling pathway	3	17	0.87	0.0403	TGFB1, TNFSF10, IL6

Pathways marked in red demonstrate proteins included in viral infection pathway on the Figure 5b

**KEGG pathways upregulated (asthma)**						
**#term ID**	**term description**	**observed gene count**	**background gene count**	**strength**	**false discovery rate**	**matching proteins in your network (labels)**
map04060	Cytokine-cytokine receptor interaction	8	143	0.7	0.0021	IL4, IL1A, IL10RB, TNFRSF11B, CD40, IL24, TNFSF11, TNFRSF9

Pathways marked in red demonstrate proteins included in cytokine-mediated signaling pathway on the Figure 5b

Data analyzed with STRING[5], false discovery rates presented.

Supplementary Table 6. Protein concentrations in apical compartment of in vitro-cultured human bronchial epithelial cells from control individuals and patients with asthma in indicated conditions.

**Control (n=6)** pg/mL (mean, SEM)	**Medium**	**RV-A16**	**SARS-CoV-2**	**RV-A16 + SARS-CoV-2**	**HDM**	**HDM+ RV-A16**	**HDM + SARS-CoV-2**	**HDM+RV-A16+ SARS-CoV-2**
**CCL8**	0.54 (0.34)	47.59 (34.12)	0.5653 (0.23)	51.92 (49.15)	0.2634 (0.10)	1.948 (0.79)	0.323 (0.17)	15.49 (13.75)
**IL-33**	0.1295 (0.07)	0.1578 (0.06)	0.2605 (0.13)	0.1499 (0.08)	0.08414 (0.01)	0.1683 (0.04)	0.1659 (0.04)	0.09775 (0.05)
**ORL1**	1.634 (0.9)	3.504 (1.45)	1.929 (1.42)	2.841 (0.78)	2.405 (1.72)	2.066 (0.66)	1.646 (0.9)	2.159 (0.69)
**CXCL9**	18.51 (6.0)	162.3 (58.2)	18.86 (5.83)	138.8 (69.84)	19.8 (7.41)	62.01 (17.98)	19.18 (6.65)	71.54 (25.52)
**TGFA**	124.3 (40.87)	54.59 (12.11)	66.13 (25.79)	59.63 (15.16)	15.06 (4.47)	15.24 (3.78)	16.28 (4.47)	15.58 (3.40)
**IL-1β**	0.1169 (0.03)	0.1612 (0.04)	0.108 (0.04)	0.2244 (0.07)	0.09652 (0.03)	0.1301 (0.06)	0.09381 (0.04)	0.1426 (0.05)
**IL-6**	1096 (471)	2371 (683.8)	999.2 (371.3)	2330 (727.5)	275.6 (143.7)	486.9 (270.4)	321.1 (189.9)	1318 (573.3)
**TWEAK**	59.45 (11)	68.49 (18.37)	74.84 (22.27)	84.29 (20.11)	84.15 (24.78)	64.84 (12.34)	66.95 (19.20)	50.19 (9.08)
**TSLP**	2.084 (1.126)	3.382 (1.76)	1.817 (0.9)	2.17 (1.57)	1.325 (0.68)	2.764 (1.40)	0.9833 (0.42)	1.149 (0.68)
**CCL11**	1.129 (0.49)	1.045 (0.52)	1.879 (0.943)	3.534 (1.89)	1.364 (0.61)	0.7482 (0.26)	0.8634 (0.24)	1.461 (0.84)
**FLT3LG**	23.95 (8.45)	21.65 (5.39)	24.31 (7.98)	29.3 (9.04)	21.99 (6.87)	24.56 (5.47)	22.78 (7.43)	17.97 (3.83)
**IL-7**	10.44 (3.19)	10.45 (1.19)	11.15 (1.75)	15.9 (6.67)	12.86 (6.33)	9.839 (3.55)	12.82 (5.72)	9.707 (3.28)
**IL-18**	0.8378 (0.2)	3.483 (0.88)	1.703 (0.51)	10.48 (5.35)	1.544 (0.75)	3.941 (1.035)	2.284 (0.95)	5.464 (1.42)
**TRAIL**	936.5 (264.2)	2529 (453.4)	847.2 (317.9)	2388 (790.6)	556.1 (188.9)	727.9 (240.9)	755.5 (357.2)	866.5 (256.1)
**CXCL10**	427.5 (152)	1953 (0)	584.1 (290.8)	1897 (56.06)	256.5 (151.7)	1068 (322.2)	244.9 (107.0)	1505 (284.6)
**TNF**	43.64 (17.08)	51.16 (17.06)	42.88 (14.65)	50.61 (18.35)	13.2 (2.46)	26.41 (7.44)	16.11 (2.2)	24.61 (6.54)
**IL-15**	11.2 (2.89)	14.81 (1.52)	12.42 (2.05)	18.32 (3.89)	7.484 (2.12)	12.27 (1.84)	10.8 (2.74)	15.05 (2.76)
**CCL3**	2.845 (2.05)	55.28 (31.78)	1.76 (1.31)	33.65 (31.25)	0.478 (0.34)	3.045 (1.47)	0.3401 (0.08)	5.753 (2.75)
**MMP12**	5819 (1779)	6526 (2322)	5676 (2166)	9355 (2136)	6370 (2162)	5664 (2427)	5697 (1614)	4498 (859.6)
**GM-CSF**	245.7(136.1)	140.6 (42.81)	184.6 (86.56)	134.5 (61.70)	93.53 (26.20)	81.97 (24.17)	88.92 (28.91)	81.14 (25.13)
**G-CSF**	544.7 (194.1)	1323 (565)	724.2 (289.1)	1541 (705.7)	326.9 (67.92)	1597 (875.9)	416.4 (78.94)	880.5 (468.2)
**VEGFA**	5117 (854.2)	4937 (490.5)	5081 (682.0)	5977 (512.6)	3708 (890)	4370 (398.6)	4404 (798.4)	4600 (447)
**IL-17C**	353 (212.1)	2692 (1620)	248.7 (140.5)	1798 (1271)	222.7 (129.1)	1160 (686)	257.4 (135.4)	764.6 (486.1)
**EGF**	61.37 (4.98)	75.64 (14.18)	79.93 (14.94)	83.55 (20.95)	128.1 (20.64)	91.63 (21.48)	112.5 (28.93)	74.64 (18.36)

**M-CSF**	553.2 (90.8)	546.7 (83.37)	575.7 (96.85)	666 (84.35)	671.8 (86.24)	676.9 (69.43)	717.9 (75.12)	625.7 (92.08)
**CCL4**	7.082 (4.245)	126.4 (70.37)	6.278 (4.98)	54.75 (49.77)	0.7136 (0.36)	6.525 (2.6)	0.6488 (0.15)	8.458 (3.73)
**CXCL11**	8.311 (3.24)	730.3 (313.9)	13.26 (4.31)	441 (304.2)	2.377 (1.26)	27.97 (13.82)	6.446 (3.42)	215.8 (177.5)
**CCL7**	5.086 (2.83)	4.915 (1.46)	7.593 (5.12)	9.32 (4.30)	2.813 (1.31)	1.624 (0.91)	3.124 (2.24)	1.935 (0.69)
**MMP1**	1640 (565.9)	1089 (237.1)	1352 (554.6)	1501 (477.3)	1534 (474.4)	1157 (313.3)	1403 (495.8)	886.5 (247.5)

RV-A16, rhinovirus A16; *SARS-CoV-2*, severe acute respiratory syndrome coronavirus 2; *HDM*, house dust mite

**Asthma (n=7)** pg/mL (mean, SEM)	**Medium**	**RV-A16**	**SARS-CoV-2**	**RV-A16 + SARS-CoV-2**	**HDM**	**HDM+ RV-A16**	**HDM + SARS-CoV-2**	**HDM+RV-A16+ SARS-CoV-2**
**CCL8**	0.3735 (0.13)	37.24 (31.0)	0.2833 (0.09)	24.65 (21.61)	0.3513 (0.16)	5.745 (4.20)	0.2681 (0.12)	15.86 (9.54)
**IL-33**	0.1176 (0.04)	0.1798 (0.05)	0.2863 (0.012)	0.1963 (0.05)	0.2795 (0.1)	0.2053 (0.04)	0.2747 (0.07)	0.4055 (0.06)
**ORL1**	1.386 (0.63)	2.56 (0.76)	1.468 (0.60)	2.276 (0.80)	1.655 (1.04)	2.14 (0.93)	2.013 (1.18)	3.591 (1.5)
**CXCL9**	18.67 (5.78)	136.5 (59.4)	164.6 (83.7)	14.37 (5.73)	14.37 (5.73)	71.73 (20.22)	117.3 (44.20)	117.3 (44.20)
**TGFA**	60.67 (15.05)	108.2 (63.87)	56.12 (21.6)	14.37 (5.73)	20.84 (5.40)	17.84 (4.47)	17.84 (4.47)	21.67 (4.20)
**IL-1β**	0.1052 (0.03)	0.1855 (0.06)	0.1784 (0.06)	0.1784 (0.06)	0.1163 (0.03)	0.1258 (0.04)	0.1258 (0.04)	0.2083 (0.08)
**IL-6**	1427 (469.8)	4940 (2138)	1389 (462.2)	5659 (2976)	517.5 (201.3)	1029 (333.8)	638.2 (222.9)	3108 (1262)
**TWEAK**	45.86 (14.15)	75.49 (28.9)	48.83 (12.82)	36.49 (7.09)	111.1 (46.41)	84.15 (28.88)	108.7 (27.26)	114.2 (44.07)
**TSLP**	1.285 (0.62)	2.397 (0.88)	1.122 (0.49)	1.972 (0.78)	1.612 (0.68)	1.662 (0.59)	1.108 (0.5)	1.977 (0.69)
**CCL11**	1.607 (0.57)	2.699 (1.24)	1.232 (0.43)	1.418 (0.48)	2.788 (1.7)	1.958 (0.99)	2.432 (1.63)	3.436 (1.861)
**FLT3LG**	17.62 (4.26)	22.79 (6.04)	20.39 (5.57)	18.91 (3.90)	35.2 (11.4)	31.07 (7.31)	31.37 (9.15)	33.38 (7.93)
**IL-7**	10.64 (2.96)	16.62 (7.54)	11.24 (3.26)	8.352 (2.33)	20.22 (11.24)	15.46 (7.17)	18.52 (10.72)	22.19 (11.40)
**IL-18**	1.188 (0.31)	6.262 (1.3)	1.531 (0.39)	8.199 (2.65)	1.706 (0.59)	7 (2.30)	2.059 (0.54)	7.617 (2.1)
**TRAIL**	646.6 (83.26)	2185 (437.6)	806.9 (159)	1617 (389.8)	850.4 (316.2)	973.8 (211.1)	707.2 (232.9)	1686 (334.7)
**CXCL10**	858.8 (188.5)	2181 (313.9)	772.2 (115.7)	7280 (5298)	283.2 (146.6)	1627 (187.8)	271.4 (121.3)	2284 (537.6)
**TNF**	41.68 (20.80)	114.6 (74.73)	39.46 (19.62)	112.8 (82.54)	17.82 (4.08)	34.28 (10.50)	17.13 (3.52)	52.82 (24.40)
**IL-15**	9.632 (1.2)	17.16 (1.65)	14.73 (2.66)	19.85 (3.11)	6.535 (1.57)	17.67 (2.99)	10.81 (1.43)<br>24.51 (3.11)	
**CCL3**	5.597 (3.86)	179.8 (107.8)	4.502 (2.66)	195.3. (122.2)	0.5091 (0.14)	10.47 (3.56)	0.8367 (0.46)	42.03 (27.17)
**MMP12**	5898 (1597)	5635 (1098)	5111 (1077)	4378 (913.8)	8939 (1967)	8676 (1819)	8697 (2023)	7781 (2062)
**GM-CSF**	306.4 (189)	530.4 (398.9)	357.5 (228.4)	425.6 (333.8)	182 (79.9)	195.7 (88.47)		424 (244.1)

							181.2 (76.55)	
**G-CSF**	529.1 (189.2)	1199 (520.8)	424.3 (154.6)	643 (240.2)	773 (501.3)	936.1 (289.4)	561.8 (261.4)	1286 (388.6)
**VEGFA**	4994 (925.1)	5004 (763.7)	5079 (737.5)	4807 (344.1)	5152 (1041)	4979 (750.1)	5210 (904.8)	5921 (705.3)
**IL-17C**	429 (284.7)	2372 (1406)	315.8 (219.7)	2299 (1424)	212.2 (134.2)	1191 (687.1)	266.6 (188.9)	1762 (1116)
**EGF**	54.85 (11.42)	66.9 (17.86)	56.51 (!2.82)	42.4 (7.82)	145.1 (54.5)	95.03 (22.5)	135.3 (43.4)	128.7 (39.12)
**M-CSF**	296 (81.5)	390.5 (97.45)	335.4 (70.62)	308.2 (54.44)	499.9 (120.9)	531.4 (100.7)	539.5 (124.2)	548.6 (113.2)
**CCL4**	7.631 (4.43)	121.9 (58.25)	5.711 (3.82)	100.2 (62.16)	0.4925 (0.21)	8.458 (2.84)	0.6477 (0.23)	23.97 (10.88)
**CXCL11**	13.58 (2.46)	713.9 (247.3)	17.86 (1.78)	620.3 (295.5)	1.832 (0.79)	48.19 (19.29)	6.228 (2.37)	250.3 (144.8)
**CCL7**	10.41 (4.58)	16.73 (8.34)	7.698 (3.47)	6.804 (2.40)	9.181 (6.24)	3.965 (1.97)	9.201 (7.40)	11.39 (7.12)
**MMP1**	1088 (393.3)	1654 (718.6)	984.9 (361.2)	717.4 (238.9)	1989 (813.8)	1793 (638.2)	1577 (522.9)	2451 (869.7)

RV-A16, rhinovirus A16; *SARS-CoV-2*, severe acute respiratory syndrome coronavirus 2; *HDM*, house dust mite

Supplementary Table 7. Allergen extracts characteristics.

	**HDM extract (Allergopharma)**	**HDM extract B (Citeq)**	**Alternaria alternata medium (Citeq)**
Protein content	0.525 mg protein/mg extract	0.216 mg protein/ mg extract	0.204 mg protein/ mg extract
Endotoxin content	6.27 EU/mg extract	51 EU/mg extract	-
Der p 1	46.66 µg/mg extract	21 µg/mg extract	-
Der p 2	-	2.9 µg/mg extract	-
Alt 1	-	-	3 µg/mg extract

EU, endotoxin units, *Der p 1*, house dust mite Der p1 allergen; *Der p 2*, house dust Mite Der p 2 allergen, *Alt 1*, Alternaria alternata Alt 1 allergen.

Supplementary Table 8. Commercially available reagents used in the study.

Reagent	Catalog number	Company
Human Bronchial Epithelial cells	EP51AB	Epithelix
Human Bronchial Epithelial cells	CC-2540	Lonza
THP-1 cells	thpx-sp	Invivogen
House dust mite extract	02.01.64	Citeq
Human Rhinovirus A16	C1404B	Virapur
Diesel exhaust matter (DEP)	NIST®SRM®2975	National Institute of Standards and Technology
Alternaria alternata extract (A. alternata)	09.01.26	Citeq
Ac-YVAD-cmk caspase-1 inhibitor	inh-yvad	Invivogen
MCC950, NLRP3 inflammasome inhibitor	AV02509	Avistron
TBK1/IKKε inhibitor BX795	702675-74-9	Sigma-Aldrich
LPS	tlrl-3pelps	Invivogen
ATP	tlrl-atp	Invivogen
BEGM Bronchial Epithelial Growth Medium BulletKit	CC3171+CC-4175	Lonza
DMEM	41965-039	Gibco
MucilAir Medium	EP03MD	Epithelix
RPMI-1640	R8758-500ml	Sigma-Aldrich
Opti-MEM® I Reduced Serum Medium	31985070	LifeTechnologies
Corning Inserts in 24-well culture plates, sterile	3470-COR	Corning
Retinoic Acid	R2625-50mg	Sigma-Aldrich
Trypsin-EDTA (0.5%)	15400-054	Thermofisher Scientific
Human IL1B/IL-1F2 duo set	DY201	R&D systems
TMB Substrate Reagent Kit	555214	BD
V-Plex Human IL1B kit	K151QPD-1	MSD
RIPA Lysis and Extraction Buffer	89901	ThermoFisher Scientific
cOmplete™, Mini, EDTA-free Protease Inhibitor Cocktail	4693159001	Merc (Roche)
Pierce BCA Protein Assay Kit	23225	ThermoFisher Scientific
4–20% Mini-PROTEAN® TGX™ Precast Protein Gels, 10-well, 50 µl	4561094	Biorad
Trans-Blot® Turbo™ Mini Nitrocellulose Transfer Packs	1704158	Biorad
10x Tris/Glycine/SDS Running Buffer	161-0772	Biorad
SurePAGE™, Bis-Tris, 10x8, 4-20%, 12 wells	M00656	GenScript
Nitrocellulose Transfer Membrane, 0.22um	L-08006-001	Advansta
WesternBright Sirius HRP substrate	K-12043-C20	Advansta
Restore™ PLUS Western Blot Stripping Buffer	46428	ThermoFisher Scientific
SuperSignal West Femto Maximum Sensitivity Substrate	34095	ThermoFisher Scientific
2-mercaptoetanol	63689-100ml-F	Sigma-Aldrich
RNAeasy Plus Micro Kit	74034	Qiagen
RevertAid RT Reverse Transcription Kit	K1691	ThermoFisher Scientific
Maxima SYBR Green/ROX qPCR Master Mix (2X)	K0221	ThermoFisher Scientific
RNAlater Stabilization Solution	1018087	Qiagen
SuperScript IV VILO Master Mix	11756050	ThermoFisher Scientific
RecoverAll	AM1975	ThermoFisher Scientific
FSC22 Frozen Section Media	3801480	Leica
ProLong Diamond Antifade Mountant with DAPI	P36962	ThermoFisher Scientific
TaqMan™ 2019-nCoV Assay Kit v1*	A47532	ThermoFisher Scientific
RV_20f2 TaqMan Assay - Vi99990017_po	A41333	ThermoFisher Scientific
**Western Blot analyses**		
Mouse anti-NLRP3	AG-20B-0014-C100	Adipogen
Goat anti-IL1B	AF-201-NA	R&D systems
Mouse anti-ASC	sc-514414	Santa Cruz Biotechnology

Rabbit anti-Caspase-1	2225	Cell Signaling
Goat anti-RIG-I	sc-48929	Santa Cruz Biotechnology
HRP Goat Anti Mouse IgG	111-035-146	Jackson Laboratory
HRP Mouse anti-goat IgG	sc2354	Santa Cruz Biotechnology
HRP AffiniPure Goat Anti Rabbit IgG	111-035-003	Jackson Laboratory
HRP Anti-beta Actin	ab49900	Abcam
Direct-Blot HRP anti-beta-actin antibody	664803	Biolegend
**Co-immunoprecipitation analyses**		
Rabbit anti-ASC	sc22514-R	Santa Cruz Biotechnology
Protein A Beads	161-0413	Biorad
Mouse anti-RIG-I	sc376845	Santa Cruz Biotechnology
Rabbit anti-MDA5	ab126630	Abcam
HRP Goat Anti Mouse IgG	115-035-146	Jackson Laboratory
HRP AffiniPure Goat Anti Rabbit IgG	111-035-003	Jackson Laboratory
**Western Blot from apical compartments analyses**		
Goat anti-IL1B	AF-201-NA	R&D systems
HRP Mouse anti-goat IgG	sc2354	Santa Cruz Biotechnology
**Confocal staining in HBECs**		
Mouse IgG1 anti-IL1B	ab156791	Abcam
Mouse IgG1 anti-RIG-I	sc-376845	Santa Cruz Biotechnology
Mouse IgG1 anti-ASC	sc-514414	Santa Cruz Biotechnology
Goat anti-mouse IgG, Alexa Fluor 546	A11003	Invitrogen
Goat anti-mouse IgG, Alexa Fluor 488	A11001	Invitrogen
Mouse IgG1 Control	X0931	Dako
Goat serum (Normal)	X0907	Dako
**Confocal staining in bronchial brushings**		
Rabbit anti-Caspase-1	2225	Cell Signalling
Mouse IgG1 anti-IL1B	ab156791	Abcam
Mouse IgG1 anti-RIG-I	sc-376845	Santa Cruz Biotechnology
Goat anti-Rabbit IgG, Alexa Fluor 488	A11034	Invitrogen
Goat anti-mouse IgG, Alexa Fluor 546	A11003	Invitrogen
Goat anti-mouse IgG, Alexa Fluor 546	A11003	Invitrogen
Rabbit Immunoglobin Fraction	X0936	Dako
Mouse IgG1 Control	X0931	Dako
Goat Serum (Normal)	X0907	Dako
**Confocal staining for NLRP3 and SARS-CoV-2 experiment**		
Anti ACE2	ab15348	Abcam
Anti NLRP3	AG-20B-0014-C100	Adipogen
Anti Occludin	OC-3F10	ThermoFisher
Anti N-Protein	MA1-7404	ThermoFisher
Rabbit Ig Fraction	X0936	DAKO
Mouse IgG1	X0931	DAKO
Mouse IgG2b	X0944	DAKO
Goat anti-Mouse IgG Alexa 488	A11001	Invitrogen
Goat anti-Rabbit IgG Alexa 546	A11010	Invitrogen
Goat anti mouse IgG2b	A21143	Invitrogen
DAPI	10236276001	Sigma Aldrich

Supplementary Table 9. Clinical characteristics of study participants.

In vivo RV-A16 infection cohort (in vivo)									
**Condition**	**Gina status**	**ACQ status at baseline[$]**	**Age (years, age range)**	**Sex**	**Drugs**	**FeV1 (%) day 0 (% predicted)**	**Total serum IgE (IU/mL)**	**Total SPT weal size (mm)**	**HDM SPT weal size (mm)**
Control	Healthy	Healthy	21-25	Male	na	113	22	0	0
Control	Healthy	Healthy	21-25	Male	na	97	16	0	0
Control	Healthy	Healthy	21-25	Male	na	97	16	0	0
Control	Healthy	Healthy	46-50	Female	na	88	19	0	0
Control	Healthy	Healthy	31-35	Female	na	103	13	0	0
Control	Healthy	Healthy	26-30	Male	na	105	3	0	0
Control	Healthy	Healthy	26-30	Female	na	105	38	0	0
Control	Healthy	Healthy	51-55	Male	na	105	16	0	0
Control	Healthy	Healthy	16-20	Male	na	100	14	0	0
Asthma	mild	well	26-30	Female	SABA	113	207	5	0
Asthma	moderate	poor	31-35	Male	ICS	70	870	11	3
Asthma	mild	well	21-25	Male	SABA	91	121	14	4
Asthma	moderate	well	16-20	Female	ICS	92	64	16	0
Asthma	moderate	well	46-50	Female	ICS	88	57	8	0
Asthma	mild	partial	31-35	Male	SABA	79	806	13	5
Asthma	moderate	partial	31-35	Female	ICS	65	507	8	0
Asthma	moderate	poor	31-35	Male	ICS	84	146	12	3
Asthma	moderate	partial	46-50	Male	SABA	73	1204	7	4
Asthma	mild	partial	31-35	Female	SABA	78	119	6	6
Asthma	moderate	poor	26-30	Male	SABA	73	19	11	4
Asthma	moderate	poor	51-55	Male	ICS	83	157	26	5
Asthma	moderate	partial	31-35	Female	ICS	77	67	7	0
Asthma	moderate	poor	41-45	Female	ICS	68	1593	3	4
Asthma	mild	well	21-25	Female	SABA	115	213	10	4
Asthma	moderate	poor	41-45	Male	ICS	74	228	17	0
Asthma	moderate	well	46-50	Male	ICS	81	87	9	3
Asthma	moderate	partial	46-50	Female	ICS	82	2106	29	9
Asthma	mild	well	51-55	female	SABA	101	69	14	5

RV-A16; rhinovirus A16*; GINA*; global initiative for asthma; *ACQ*, asthma control questionnaire; *FeV1*, forced expiratory volume in 1 second; *IgE*, immunoglobulin E; *SPT*, skin prick test; *HDM*, house dust mite; *na*, not applicable; *SABA*, short acting beta agonists; *ICS*, inhaled corticosteroids. $ Well controlled=ACQ score ≤0.75, partially controlled=ACQ score 0.76-1.49, poorly controlled=ACQ score ≥1.50, *=summation of all positive individual allergen weal sizes

**Cohort SIBRO (in vivo BAL IL-1ß assessment and in vitro ALI cultures of HBECs)**				
**Obesity Status**	**Asthma Status**	**Age (years, age range)**	**Sex**	**BMI (kg/m^2)**
Non-Obese	Non-asthma	41-45	Male	24.61
Non-Obese	Non-asthma	26-30	Male	21.62
Non-Obese	Non-asthma	26-30	Female	21.01
Non-Obese	Non-asthma	46-50	Male	23.57
Non-Obese	Non-asthma	41-45	Male	22.86
Non-Obese	Non-asthma	41-45	Female	22.21
Non-Obese	Non-asthma	51-55	Female	24.14
Non-Obese	Asthma	31-35	Male	21.74
Non-Obese	Asthma	26-30	Female	24.8
Non-Obese	Asthma	21-25	Male	23.94
Non-Obese	Asthma	26-30	Male	22.69
Non-Obese	Asthma	51-55	Female	21.56
Non-Obese	Asthma	36-40	Female	19.92
Non-Obese	Asthma	46-50	Female	21.1
Non-Obese	Asthma	46-50	Female	23.42
Non-Obese	Asthma	41-45	Male	25.21
Non-Obese	Asthma	51-55	Female	21.56
Non-Obese	Asthma	51-55	Female	21.83
Non-Obese	Asthma	56-60	Female	24.68

ALI, Air-liquid interface; *BMI*, body max index; HBECs, Human Bronchial Epithelial Cells

**Cohort A (in vitro ALI-cultures of HBECs)**		
**Diagnosis**	**Sex**	**Age (years, age range)**
Control	Female	26-30
Control	Male	26-30
Control	Male	61-65
Control	Male	41-45
Control	Female	31-35
Asthma	Female	26-30
Asthma	Female	61-65
Asthma	Female	26-30
Asthma	Female	61-65
Asthma	Female	51-55
Asthma	Female	41-45
Asthma	Female	36-40
Asthma	Female	61-65
Asthma	Male	51-55
Asthma	Male	56-60
Asthma	Male	31-35
Asthma	Male	51-55

ALI, Air-liquid interface; *HBECs*, Human Bronchial Epithelial Cells

Supplementary Table 10. Detailed study cohorts' description.

Cohort	Samples origin	Status of the ethics	Type of material	Storage conditions	Date of sample processing/analysis	Linked publications
**In vivo RV-A16 infection**<br><br>Experimental in vivo RV-A16 infection in healthy controls and patients with asthma	Imperial College, London, United Kingdom<br><br>All samples used in the current manuscript were collected during original sampling.[6] No additional infections were performed for the purposes of the current manuscript.	St. Mary`s Hospital Research Ethics Committee (09/H712/59)<br><br>Registration number of the observational study: NCT01159782<br><br>Patients consent was obtained at the time of sampling<br><br>Further use and additional analysis of the samples permitted and consented<br><br>RNA microarray analysis permitted and consented	Bronchial brushings	Brushings pelleted immediately after collection and lysed for RNA extraction; lysates stored in -80°C	Collected: 2009-2011<br>Microarray performed: 2011<br>Bioinformatics: 2020/21	**6, 7, 8, 9, 10, 11, 12, 13, 14, 15, 16, 17, 18**
			Bronchial biopsies	Paraffin blocks, RT	Collected: 2009-2011<br>Analyzed: 2018/19	
			BAL, NL	-80°C	Collected: 2009-2011<br>Analyzed: 2012/18/19	
**SIBRO**<br><br>Patients with asthma and healthy controls sampled at the steady state	Pulmonary Division, University Hospital of Zurich, Switzerland<br><br>ALL-MED Medical Research Institute, Wroclaw, Poland<br><br>All samples used in the current manuscript were collected during original sampling.[19] No additional sampling was performed for the purposes of the current manuscript.	KEK-ZH-Nr. 20212-0043 – Kantonale Ethik-Kommission Zürich;<br><br>KB-70/2013 and KB-567/2014 – Bioethical Committee, Wroclaw Medical University<br><br>Patients consent was obtained at the time of sampling<br><br>Further use and additional analysis of the samples permitted and consented	BAL	-80°C	Collected: 2016<br>Analyzed: 2017	**19, 20**
			HBECs	Freshly isolated from bronchial brushings; cryopreserved in liquid nitrogen	Collected: 2016<br>Analyzed: 2017-2022	
**Cohort A**<br><br>Patients with asthma and healthy controls sampled at the steady state	University Hospital, Jagiellonian University Medical College, Cracow, Poland<br><br>Biobank of HBECs; collected and deposited in Department of Medicine, Laboratory of Molecular Biology and Clinical Genetics, Cracow, Poland<br><br>All samples used in the current manuscript were collected during the original sampling. No additional sampling was performed for the purposes of the current manuscript.	Jagiellonian University Bioethics Committee (KBET/68/B/2008 and KBET/209/B/2011)<br><br>Patients consent was obtained at the time of sampling<br><br>Further use and additional analysis of the samples permitted and consented<br><br>RNA sequencing analysis permitted and consented	HBECs	Freshly isolated from bronchial brushings; cryopreserved in liquid nitrogen	Collected: 2010-2013<br>Analyzed: 2017-2022	**20, 21, 22, 23, 24, 25**
			HBECs	ALI-differentiated HBECs processed for RNA-seq and stored in -80°C	Analyzed: 2017-2022	

BAL, bronchoalveolar lavage fluid; *HBECs*, human bronchial epithelial cells; *NL*, nasal lavage; *RT*, room temperature.

Supplementary Table 11. Primary human bronchial epithelial cells (HBECs) used in the study for in vitro Air-liquid interface cultures. Commercially available and obtained from the study subjects.

(in vitro ALI-cultures of HBECs)				
**HBECs source**	**Condition**	**Age (years, age range)**	**Sex**	**Smoker**
Epithelix	Healthy	71-75	Male	No
Epithelix	Healthy	51-55	Male	No
Epithelix	Healthy	76-80	Male	No
Epithelix	Healthy	51-55	Male	No
Epithelix	Healthy	61-65	Male	No
Epithelix	Healthy	56-61	Male	No
Lonza	Healthy	66-70	Female	No
Lonza	Healthy	21-25	Female	No
Epithelix	Healthy	51-55	Female	No
Epithelix	Healthy	16-20	Male	No
Epithelix	Healthy	61-65	Female	No
Epithelix	Healthy	16-20	Male	No
Epithelix	Healthy	56-61	Male	No
Epithelix	Healthy	51-55	Male	No
Epithelix	Healthy	56-61	Female	No
Epithelix	Healthy	71-75	Male	No
Epithelix	Healthy	61-65	Male	No
Epithelix	Asthma	36-40	Male	No
Epithelix	Asthma	51-55	Male	No
Epithelix	Asthma	41-45	Male	No
Epithelix	Asthma	56-60	Male	No
Lonza	Asthma	66-70	Male	No
Lonza	Asthma	61-65	Female	No
Lonza	Asthma	11-15	Female	No
Lonza	Asthma	21-25	Male	No
Epithelix	Asthma	16-20	Female	No
Epithelix	Asthma	46-50	Male	No
Epithelix	Asthma	76-80	Female	No
Epithelix	Asthma	51-55	Female	No
Epithelix	Asthma	46-50	Female	No
Epithelix	Asthma	56-61	Female	No

ALI, Air-liquid interface; *HBECs*, Human Bronchial Epithelial Cells

(in vitro ALI-cultures of HBECs)			
**Cell source**	**Condition**	**Age (years, age range)**	**Sex**
Cohort A	Control	51-55	Female
Cohort A	Control	71-75	Male
Cohort SIBRO	Non-asthma	31-35	Female
Cohort A	Asthma	46-50	Female
Cohort A	Asthma	36-40	Female
Cohort A	Asthma	51-55	Male
Cohort A	Asthma	61-65	Male
Cohort A	Asthma	46-50	Female
Cohort A	Asthma	36-40	Male
Cohort A	Asthma	21-25	Female
Cohort A	Asthma	61-65	Female
Cohort A	Asthma	26-30	Female
Cohort A	Asthma	66-70	Female
Cohort A	Asthma	61-65	Female
Cohort A	Asthma	51-55	Female
Cohort A	Asthma	61-65	Female
Cohort A	Asthma	35-41	Female
Cohort A	Asthma	56-61	Female
Cohort A	Asthma	56-61	Female
Cohort A	Asthma	26-31	Female
Cohort A	Asthma	36-41	Male
Cohort A	Asthma	51-55	Male
Cohort A	Asthma	56-61	Male
Cohort SIBRO	Asthma	41-45	Male
Cohort SIBRO	Asthma	31-35	Male
Cohort SIBRO	Asthma	51-55	Male

ALI, Air-liquid interface; *HBECs*, Human Bronchial Epithelial Cells

Supplementary Table 12. Sequences of the primers used in the study.

Gene	Forward primer sequence	Reverse primer sequence
IL1B	CTCTTCGAGGCACAAGGCA	GGCTGCTTCAGACACTTGAG
RV-A16 POSITIVE STRAND	CGGGACTGCAAACACTACCT	CACCACGTGTGTCCCTAACA
DDX58	TGATTGCCACCTCAGTTGCT	TCCTCTGCCTCTGGTTTGGA
NLRC5	GCTGGAGGAGGTCAGTTTGC	TGTTTCGGCTCAGGTCAAGT
IFIH1	AGATGCAACCAGAGAAGATCCA	TGGCCCATTGTTCATAGGGT
CASP1	GCCCACCACTGAAAGAGTGA	TTCACTTCCTGCCCACAGAC
IFNL2/3	CTGGGAGACAGCCCAGTTCA	AGAAGCGACTCTTCTAAGGCATCTT
IFNB1.1	CGCCGCATTGACCATCTA	GACATTAGCCAGGAGGTTCTC
IFNB1.2	AGGCCAAGGAGTACAGTCAC	GAGGTAACCTGTAAGTCTGTTAATG
EEFA1	TGAAGTCTGGTGATGCTGCC	CAAAGCGACCCAAAGGTGGA
IL-18	TGCAGTCTACACAGCTTCGG	GCAGCCATCTTTATTCCTGCG
ICAM-1	ACCATCTACAGCTTTCCGGC	CTTCACTGTCACCTCGGT
**Gene**	**Primer pair ID**	**Company**
IFNL1	H_IL29_1	Sigma Aldrich
TSLP	H_TSLP_1	Sigma Aldrich
IL-33	H_IL33_1	Sigma Aldrich
GSDMD	H_GSDMD_1	Sigma Aldrich

Supplementary Table 13. List of genes included in inflammasome-mediated immune responses and antiviral responses gene sets. Curated from the gene sets available at the GSEA and MSigDB Databases.

Inflammasome-mediated immune responses	*TXNIP, NLRP3, NLRP1, PANX1, PYCARD, HSP90AB1, APP, MEFV, P2RX7, NLRC4, BCL2, BCL2L1, TXN, CASP1, PSTPIP1, AIM2, CASP4, CASP5, CASP8, NLRC5, DDX58, IFIH1, IL18, NLRP7, NOD1, TAB1, IRAK3, CHUK, MAP3K8, TAB2, PELI3, TAB3, IKBKB, IL1A, IL1B, IL1R1, IL1RAP, IL1RN, IRAK1, IRAK2, TMEM189-UBE2V1, MAP3K3, MYD88, IRAK4, TOLLIP, MAP2K1, MAP2K6, PELI2, PELI1, MAP2K4, SKP1, MAP3K7, TRAF6, UBE2N, IL1R2, TNIP2, CUL1, IKBKG, RIPK2, SQSTM1, BTRC, RBX1, NAIP, CARD18, CD40LG, CTSB, HSP90AA1, HSP90B1, PYDC1, SUGT1, TNF, TNFSF11, TNFSF14, TNFSF4, IFNG, IL12A, IL12B, IL33, IRF1, TIRAP, CIITA, NLRP12, NLRP4, NLRP5, NLRP6, NLRP9, NLRX1, BIRC2, BIRC3, CARD6, CCL2, CCL5, CCL7, CFLAR, CXCL1, CXCL2, FADD, IFNB1, IL6, IRF2, MAPK1, MAPK11, MAPK12, MAPK13, MAPK3, MAPK8, MAPK9, NFKB1, NFKBIA, NFKBIB, PEA15, RELA, XIAP, NOD2*
Antiviral responses	*ABCC9, ABCE1, ABCF3, ACTA2, ADAR, ADARB1, AGBL4, AGBL5, AIMP1, AP1S1, APOB, APOBEC1, APOBEC3A, APOBEC3B, APOBEC3C, APOBEC3D, APOBEC3F, APOBEC3G, APOBEC3H, ATG7, AZU1, BAD, BANF1, BATF3, BCL2, BCL2L1, BCL2L11, BCL3, BECN1, BNIP3, BNIP3L, BST2, BTBD17, C17orf85, C19orf2, C19orf66, CARD9, CCDC130, CCL11, CCL19, CCL22, CCL4, CCL5, CCL8, CCT5, CD207, CD40, CD86, CD8A, CDK6, CFL1, CHRM2, CHUK, CLU, CRCP, CREBZF, CXADR, CXCL10, CXCL12, CXCL9, CXCR4, CYP1A1, DCLK1, DDIT4, DDX1, DDX21, DDX3X, DDX41, DDX58, DDX60, DEFA1, DEFA1B, DEFA3, DHX36, DHX58, DMBT1, DNAJC3, DUOX2, EEF1G, EIF2AK2, EIF2AK4, ELMOD2, ENO1, EXOSC4, EXOSC5, F2RL1, FADD, FAM111A, FCN3, FGR, FOSL1, FOXP3, GATA3, GBP1, GBP3, GLI2, GPAM, GTF2F1, HBXIP, HERC5, HMGA1, HMGA2, HNRNPUL1, HSPB1, HYAL1, HYAL2, HYAL3, IFI16, IFI44, IFI44L, IFIH1, IFIT1, IFIT1B, IFIT2, IFIT3, IFIT5, IFITM1, IFITM2, IFITM3, IFNA1, IFNA10, IFNA13, IFNA14, IFNA16, IFNA17, IFNA2, IFNA21, IFNA4, IFNA5, IFNA6, IFNA8, IFNAR1, IFNAR2, IFNB1, IFNE, IFNG, IFNGR1, IFNGR2, IFNK, IFNW1, IKBKB, IKBKE, IKBKG, IL10RB, IL12A, IL12B, IL23A, IL28A, IL28B, IL28RA, IL29, IL33, IL6, ILF3, IRAK3, IRF1, IRF3, IRF5, IRF7, IRF9, ISG15, ISG20, ITCH, IVNS1ABP, KCNJ8, LGALS9, LILRB1, LSM14A, LYST, MAPK11, MAPK14, MAVS, MB21D1, MEF2C, MICA, MST1R, MX1, MX2, NLRC5, NLRP3, NPC2, OAS1, OAS2, OAS3, OASL, ODC1, OPRK1, PCBP2, PENK, PIM2, PLSCR1, PMAIP1, PML, POLR3A, POLR3B, POLR3C, POLR3D, POLR3E, POLR3F, POLR3G, POLR3H, POLR3K, PRF1, PRKRA, PSMA2, PSMB9, PTPRC, PYCARD, RELA, RNASEL, RPS15A, RSAD2, SAMHD1, SERINC3, SERINC5, SLFN11, SPACA3, SPON2, SRC, STAT1, STAT2, STMN1, TBK1, TBX21, TICAM1, TLR3, TLR7, TLR8, TMEM173, TNF, TNFSF4, TPT1, TRIM11, TRIM22, TRIM25, TRIM34, TRIM5, TRIM56, TRIM6, UNC13D, UNC93B1, XCL1, XPR1, ZC3H12A, ZC3HAV1, ZNF175*

Supplementary Table 14. All proteins available for PEA measurements used as statistical background for STRING analyses of targeted proteomics data.

UniprotID
P03950, Q9Y5C1, O95445, Q96KN2, P15907, P12830, P00915, P07451, P22748, Q9NQ79, P49747, Q16627, P55774, P13501, P13987, P08709, P03951, P39060, Q9BXJ1, P06681, Q9BXR6, P20023, P01034, P27487, Q12805, P17813, Q9UGM5, Q15485, Q16769, P22749, Q14393, P08581, P0DOY2, P17936, P24592, P11215, P05362, P32942, P16871, Q14767, Q8NHL6, Q8N423, O75023, P05451, P23141, P12318, O75015, P14151, Q9Y5Y7, P42785, P11226, P10721, P15529, Q16853, P01033, Q13361, Q9H1U4, P13591, O00533, P46531, O14786, P59665, P80188, P14543, Q99650, P19021, P55058, P05154, P07359, Q13093, O15031, Q15113, Q12884, Q13332, Q06141, P35542, Q14515, P00441, Q96H15, P24821, P22105, P35443, P05543, P20062, Q03167, Q15582, P07478, P35590, P07911, P19320, Q6EMK4, P04070, Q86SJ2, Q7Z5R6, Q8TD06, P15848, P08237, O43521, Q02742, P21810, Q06520, P11274, Q8N5S9, Q9BQT9, Q9NX58, P04637, Q49AH0, P02462, O43186, P0CG37, Q9UBG0, Q8WYN0, P16562, Q9UK85, P25685, P52564, Q13561, P22681, Q01543, P47929, A4D1B5, Q9NS71, O00451, P01275, P01242, Q9Y662, O60243, O75054, Q9NRM6, Q9UKR0, Q96I82, Q6UXK5, Q8N2G4, Q12912, Q6UB28, Q16653, O94760, O00221, Q969V3, Q92982, O95644, P23515, Q9UBM4, P30041, Q9NZ53, Q10471, Q96SM3, P58294, Q9HCU5, Q7Z5A7, O14904, O14917, Q13576, P57771, Q07960, Q9NZN5, Q6UXD5, Q9C0C4, O96013, Q96LC7, O43699, Q9P0V8, Q9H156, Q9H5Y7, Q86WV1, O14662, O43752, O95988, Q9Y6A5, Q9Y2W6, P09758, O95183, Q92558, Q9UPY6, Q96PQ0, Q13105, Q16698, Q76LX8, P35318, O00253, P35475, Q9BYF1, P22004, P35218, P31997, P07711, Q99895, O94907, P12104, P19883, P27352, P51161, P01241, Q9UK05, P04792, Q9UJM8, P18510, Q9HB29, Q8TAD2, Q8NEV9, Q14213, P24394, Q04760, P78380, P41159, P06858, P31994, P47992, Q9UEW3, Q9UKP3, P16860, Q9Y6K9, Q8IYS5, Q13219, P26022, P09874, P01833, Q99075, Q14005, P51888, Q16651, P02760, P25116, P21980, P12931, Q14242, Q15109, P00797, Q9BQR3, Q13043, Q8IW75, Q9UIB8, Q9NQ25, Q99523, Q9BUD6, P04179, P07204, P40225, P40225, P13726, O14763, Q9Y6Q6, O14836, Q12866, Q96IQ7, Q9BWV1, O00220, O43915, Q15389, Q02763, Q92583, P29965, P07585, O00182, P09601, P39900, P09237, P01127, Q9BQ51, P01730, P49763, Q96D42, P15144, P20160, Q13867, P33151, P15085, P15086, P42574, P07339, Q9UBR2, Q16663, O15467, O00175, Q13740, P36222, Q13231, P02452, Q9NPY3, Q12860, Q9H2A7, P04080, P19957, P54760, P00533, P16422, P16581, P15090, P17931, P56470, P28799, Q99988, Q9HCN6, P08833, P18065, Q16270, P05107, P13598, P14778, P27930, Q96F46, O95998, P01589, P08887, Q9Y624, Q92876, P01130, P36941, Q9NQ76, P08253, P08254, P14780, Q99727, P24158, P05164, P02144, Q9UM47, P10451, Q15166, O75594, P98160, P05121, P16284, P04085, Q8NBP7, P80370, P16109, P35247, Q9HD89, Q99969, Q86VB7, Q96PL1, Q9HCB6, Q01638, P13686, P10646, P00750, P02786, Q07654, Q5T2D2, Q9Y275, P19438, P20333, O14798, Q92956, P25445, P30530, P78324, Q03405, P04275, P16860, Q10588, Q9UKK9, P16112, P05067, Q9BY76, P15289, P50895, P15291, O43505, P08236, P08118, P00918, P23280, Q9UBX1, P11717, Q6YHK3, Q8N6Q3, Q9NNX6, P48960, Q8TCZ2, Q4KMG0, Q6UXG3, O43405, Q5KU26, Q8IWV2, P24387, Q9Y240, Q86T13, Q15828, Q9NZV1, Q6UXH1, P47712, Q02487, Q9UBP4, Q14118, Q07108, Q13822, Q96AP7, Q96RD9, P30043, O95633, P21217, Q11128, P01215, P55808, Q8TDQ0, P04233, Q6UXH9, P55103, P08648, P05556, P78552, O43278, O43291, Q08431, Q16363, Q14696, Q99538, Q6GTX8, P06734, P19256, P14174, O00339, Q99972, Q14112, Q99983, Q9UKJ1, P23284, P30086, Q5VY43, P09619, Q99497, P07237, P48745, P10586, Q9Y6N7, Q14162, Q96QR1, O75326, O43464, P00995, Q9NQ38, O00241, P31948, O95721, P63313, P04066, O14773, Q969Z4, P29350, Q8TEU8, Q9Y279, Q01151, P09038, P42701, P18627, Q9UQV4, O76036, Q13241, P48061, P10747, O15123, P05089, P40933, Q9Y653, P43629, P29474, P21246, Q15116, Q8WXI7, Q5T4W7, O15169, O15444, Q9NRJ3, Q9H5V8, P80162, P28325, Q8NFT8, Q13541, O95750, P12034, P49771, Q13651, Q08334, P29460, Q13261, Q16552, Q9P0M4, Q13478, P14784, Q9NYY1, Q9UHF4, Q8N6P7, Q13007, P15018, P42702, P03956, P09238, P20783, Q99748, P13725, P80511, Q13291, Q8IXJ6, O95630, P50225, P30203, Q969D9, P01374, O14788, Q9GZV9, Q9NSA1, P10147, Q14116, P21583, O00300, P00749, P13500, P00813, Q14790, Q99731, P78556, P55773, P13236, P25942, P02778, O14625, P42830, Q07325, P78423, P01579, P01583, P35225, P60568, O95760, P05112, P13232, P10145, P01137, P09603, P80075, P80098, Q99616, Q9BZW8, Q9NZQ7, P06127, P01732, O43508, P01375, O43557, Q07011, P22301, P05113, P51671, P01138, P39905, P01135, P50591, P05231, P14210, P15692, P09341, Q7Z6M3, P27540, Q13490, P16278, P78410, Q9UHC6, Q15517, P28845, P78310, Q9UMR7, Q8WTT0, Q8WXI8, Q6UXB4, Q6EIG7, Q9BXN2, Q07065, Q13574, O00273, Q9UN19, Q14203, P19474, Q9GZT9, Q04637, P63241, Q96P31, Q6DN72, Q96DB9, P14317, P50135, P52294, Q8N608, O43736, Q9UKX5, P23229, P18564, Q8IU57, Q00978, P51617, Q9NWZ3, Q05084, P08727, Q8NHJ6, O60449, P48740, P35240, P16455, Q96SB3, P34130, Q12968, Q03431, O75475, Q06830, P30044, Q6ZUJ8, Q9HCM2, Q14435, O95786, P58499, O94992, Q04759, O43597, Q9Y2J8, O60880, Q9UQQ2, Q9Y3P8, P78362, P52823, P30048, Q12933, Q92844, O14867, Q9NP99, Q9C035, Q15661, Q9UNE0, Q96PD2, P05412, Q05516, P23526, Q9UHX3, Q8IZP9, P51693, O95841, O43827, P50995, P09525, Q9UBU3, P20711, P40259, P19022, Q9HBB8, Q9H4D0, Q8N1Q1, P21964, P43234, Q9Y5K6, Q6WN34, Q15846, Q76M96, P46109, Q9NY25, Q9H6B4, Q9NR28, P09417, Q9UHL4, P98082, P27695, O75356, Q6UWV6, P12724, Q96LA6, P09467, P22466, P09104, P35754, O75791, P51858, Q01973, Q8WX77, P26010, O43240, Q16773, P46379, Q96JA1, A6NI73, Q86VZ4, Q9NPH0, Q16820, Q641Q3, Q8NI22, O95544, Q92692, Q9NQX5, O95502, Q15155, Q9UKJ0, Q02790, Q9NWQ8, P09668, Q92520, P41236, P25815, Q9BYZ8, Q9BZR6, P00352, P16083, Q8WTU2, Q9BQB4, Q13275, P35237, P50452, Q9Y286, Q04900, Q8WVQ1, Q8NBJ7, O00161, P31431, P29017, P52888, Q8NBS9, P19971, P01222, Q03403, Q9GZM7, Q06418, P40818, P13611, P28907, P30533, Q16620, P12644, Q96GW7, Q2TAL6, P22223, P55285, P48052, P14384, P25774, Q8N126, Q8TD46, Q8TDQ1, Q08708, O94779, Q9P126, Q8IUN9, Q9UBT3, P53634, Q9P0K1, O75077, Q8NBI3, O15197, P52798, Q08345, P15311, Q96LA5, O00214, P56159, O60609, P78333, P09919, P15509, O14793, Q16775, Q01344, P57087, Q16719, Q9BS40, Q6UX15, O43155, Q6ISS4, O43561, Q14108, P21757, Q8NFP4, O15232, P55145, P10636, Q02083, P08473, O95185, P41271, O14594, Q92823, O60462, Q9NR71, Q9HAN9, Q9BZM5, Q16288, P41217, P16234, O43157, Q9ULL4, P15151, Q2VWP7, Q96B86, Q6NW40, Q9HCK4, Q2MKA7, Q6ZMJ2, Q96GP6, Q92765, P37023, Q9Y336, Q9BZZ2, Q9H3U7, P17405, Q92752, Q08629, P04216, Q9H3S3, Q9HAV5, Q96NZ8, P29460, P29459, O95727, P12544, Q9NP84, O75509, Q03393, O60242, Q9BTE6, Q9BYC5, P19801, Q9UJ72, P07306, O15263, Q86Z14, P49789, Q10589, P55291, Q12864, P63098, Q9Y2V2, O94985, P40198, Q16619, Q9Y4X3, Q8IX05, P08962, P41208, Q9Y5P4, P52943, P78560, P32926, P16444, Q9H4A9, Q13444, P51452, Q96EP0, P14625, P42892, Q5JZY3, P23588, P21802, P13284, O76070, P36269, P09211, P09466, O15496, P32456, P30519, P24071, Q6UXK2, Q9UK53, P29218, Q9H0C8, Q8IU54, P26951, P24001, Q96EK5, P43628, Q6UWL6, Q9NS15, P48357, Q9Y6D9, P20138, P41227, O00308, Q969M7, P58417, P07196, P06748, Q13451, Q9Y680, Q8TCT1, Q15126, Q9NRG1, Q9UHV9, P11464, O14944, Q96B36, Q06323, Q96IU4, Q92597, Q9H477, P23443, Q8N474, P37108, Q9H4F8, P13385, Q9BW30, Q99426, Q96RJ3, P18031, Q6UX27, Q16864, Q9UKS7, Q9P0J1, Q9Y478, P30838, O95994, O95831, Q8NDB2, P55957, Q13145, P01258, P27797, O43570, Q9ULX7, P48730, Q9HAW4, Q11201, O00748, Q02246, P06850, Q8NC01, P23582, Q86SJ6, P42658, Q02880, Q9Y5L3, O43854, P98073, Q9UHF1, Q96RT1, P01588, Q0Z7S8, Q12778, Q9NQ88, O60760, Q13308, O14713, O75569, P09960, Q9GZY6, P01229, Q7L5Y9, P40121, Q9Y5V3, P53582, Q03426, Q9Y4K4, Q15797, Q9NXA8, Q9Y5A7, P19878, O60934, P80303, P20472, P49023, P68106, O60240, O15357, Q9NRA1, Q8IUK5, Q86SR1, P35070, Q07954, P25786, Q9BXJ7, Q8N8S7, P53539, P61244, O75688, P50749, P20936, Q12913, O75787, Q9UKL0, P49788, Q7LG56, Q86WD7, Q15165, Q9UNK0, O00186, P19429, P07332, P09769, P07947, Q7L8A9, P42768, O75354, P21589, Q8TE58, P40222, P04083, P16870, P06731, P13688, O60911, O95971, P09326, Q9UBG3, Q9UJ71, P38936, O00548, P78325, Q9NQ30, P29317, Q13158, 6BAA4, Q14512, P15328, P41439, P09958, P35052, P08069, P06756, P18084, P15260, Q9UBX7, Q9UKR3, Q9P0G3, O60259, O95274, Q16674, Q13421, P50579, P21741, Q99717, Q96NY8, P01298, O00592, O00622, P31949, P26447, P07949, P04626, P21860, Q15303, Q9BXY4, O14828, Q9BYH1, P09486, P18827, P56279, P37173, P48307, Q9HBG7, O15455, O75888, Q9NS68, O95407, P00519, P07948, Q14508, P35916, Q6UXB2, P08670, Q9Y5W5, O95388, O43895, P15514, Q14956, Q16790, P26842, P32970, O43927, P48023, P09382, P10144, P20718, O75144, Q29983, Q29980, P01133, P43489, P35968, O00233, Q9UJY5, P15121, P05187, P55008, O43707, P02771, Q12904, P20273, Q9Y644, P06865, Q9BYE9, Q9P1Z2, Q9UDT6, P42575, P30260, Q99795, Q9NTU7, P0DN86, Q496F6, P20849, P02745, O95715, P28838, Q9H0P0, Q9H773, P55039, Q14241, Q9BS26, Q53H82, P55789, O60907, O43524, O60763, Q9HD26, Q14353, O14558, P09105, P13747, P01591, P49441, Q14773, Q9UMF0, P38484, P01584, Q96PD4, Q9Y5K2, P33241, Q9H8J5, Q86SF2, P32004, P62166, P43490, P09110, O60542, P08397, Q9H3G5, P12872, P01303, Q8WUW1, Q9Y2B0, Q92832, Q96FQ6, Q93096, O75695, Q08174, Q8IWL2, P20340, P36888, P28827, P42331, P35637, O76038, P13521, Q96I15, Q9UHD8, O60575, Q99536, P48643, P51580, Q96J42, P13693, O95379, Q9BSL1, Q13459, Q8NEZ2, Q5VIR6, P17948, Q7Z5L0, Q6PCB0, Q9Y5K8, Q7Z739

Supplementary References:

1. Robinson MD, McCarthy DJ, Smyth GK. edgeR: a Bioconductor package for differential expression analysis of digital gene expression data. *Bioinformatics* **26**, 139-140 (2010).

2. Ritchie ME*, et al.* limma powers differential expression analyses for RNA-sequencing and microarray studies. *Nucleic Acids Research* **43**, e47-e47 (2015).

3. Bateman ED*, et al.* Global strategy for asthma management and prevention: GINA executive summary. *Eur Respir J* **31**, 143-178 (2008).

4. Bai J*, et al.* Phenotypic responses of differentiated asthmatic human airway epithelial cultures to rhinovirus. *PLoS One* **10**, e0118286 (2015).

5. Szklarczyk D*, et al.* The STRING database in 2021: customizable protein-protein networks, and functional characterization of user-uploaded gene/measurement sets. *Nucleic Acids Res* **49**, D605-D612 (2021).

6. Jackson DJ*, et al.* IL-33-dependent type 2 inflammation during rhinovirus-induced asthma exacerbations in vivo. *Am J Respir Crit Care Med* **190**, 1373-1382 (2014).

7. Farne H*, et al.* In vivo bronchial epithelial interferon responses are augmented in asthma on day 4 following experimental rhinovirus infection. *Thorax* **77**, 929-932 (2022).

8. Williams TC*, et al.* Rhinovirus-induced CCL17 and CCL22 in Asthma Exacerbations and Differential Regulation by STAT6. *Am J Respir Cell Mol Biol* **64**, 344-356 (2021).

9. Hansel TT*, et al.* A Comprehensive Evaluation of Nasal and Bronchial Cytokines and Chemokines Following Experimental Rhinovirus Infection in Allergic Asthma: Increased Interferons (IFN-gamma and IFN-lambda) and Type 2 Inflammation (IL-5 and IL-13). *EBioMedicine* **19**, 128-138 (2017).

10. Toussaint M*, et al.* Host DNA released by NETosis promotes rhinovirus-induced type-2 allergic asthma exacerbation. *Nat Med* **23**, 681-691 (2017).

11. Beale J*, et al.* Rhinovirus-induced IL-25 in asthma exacerbation drives type 2 immunity and allergic pulmonary inflammation. *Sci Transl Med* **6**, 256ra134 (2014).

12. Jackson DJ*, et al.* Interleukin-18 is associated with protection against rhinovirus-induced colds and asthma exacerbations. *Clin Infect Dis* **60**, 1528-1531 (2015).

13. Nikonova A*, et al.* M1-like macrophages are potent producers of anti-viral interferons and M1-associated marker-positive lung macrophages are decreased during rhinovirus-induced asthma exacerbations. *EBioMedicine* **54**, 102734 (2020).

14. Upton N*, et al.* Rhinovirus induction of fractalkine (CX3CL1) in airway and peripheral blood mononuclear cells in asthma. *PLoS One* **12**, e0183864 (2017).

15. Naveed SU*, et al.* Matrix Metalloproteinase-1 Activation Contributes to Airway Smooth Muscle Growth and Asthma Severity. *Am J Respir Crit Care Med* **195**, 1000-1009 (2017).

16. Niespodziana K*, et al.* Rhinovirus-induced VP1-specific Antibodies are Group-specific and Associated With Severity of Respiratory Symptoms. *EBioMedicine* **2**, 64-70 (2015).

17. Jackson DJ, *et al.* The influence of asthma control on the severity of virus-induced asthma exacerbations. *J Allergy Clin Immunol* **136**, 497-500 e493 (2015).

18. Jayaraman A, *et al.* IL-15 complexes induce NK- and T-cell responses independent of type I IFN signaling during rhinovirus infection. *Mucosal Immunol* **7**, 1151-1164 (2014).

19. Michalovich D, *et al.* Obesity and disease severity magnify disturbed microbiome-immune interactions in asthma patients. *Nat Commun* **10**, 5711 (2019).

20. Radzikowska U, *et al.* Distribution of ACE2, CD147, CD26, and other SARS-CoV-2 associated molecules in tissues and immune cells in health and in asthma, COPD, obesity, hypertension, and COVID-19 risk factors. *Allergy* **75**, 2829-2845 (2020).

21. Wawrzyniak P, *et al.* Regulation of bronchial epithelial barrier integrity by type 2 cytokines and histone deacetylases in asthmatic patients. *The Journal of allergy and clinical immunology* **139**, 93-103 (2017).

22. Stocker N, *et al.* Regulation of ACE2 isoforms by type 2 inflammation and viral infection in human airway epithelium. *Mucosal Immunology*, (2023).

23. Wawrzyniak P, *et al.* Inhibition of CpG methylation improves the barrier integrity of bronchial epithelial cells in asthma. *Allergy* **76**, 1864-1868 (2021).

24. Wang M, *et al.* Laundry detergents and detergent residue after rinsing directly disrupt tight junction barrier integrity in human bronchial epithelial cells. *J Allergy Clin Immunol* **143**, 1892-1903 (2019).

25. Kast JI, *et al.* Respiratory syncytial virus infection influences tight junction integrity. *Clin Exp Immunol* **190**, 351-359 (2017).

Uncropped presentation of Western Blots

RIG-I protein assessed in the cell lysates

Control

Figure S4G

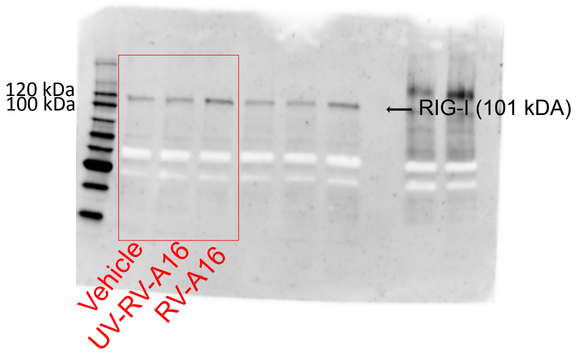

antibodies used:
goat anti-RIG-I,
sc-48929, Santa Cruz Biotechnology

HRP mouse anti-Goat IgG,
sc2354, Santa Cruz Biotechnology

β-actin protein assessed in the cell lysates

Control

Figure S4G

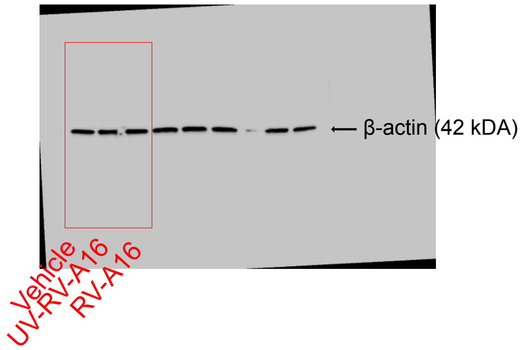

antibody used:
HRP anti-B-actin
ab49900, Abcam

NLRP3 protein assessed in the cell lysates

Control

Figure S4K

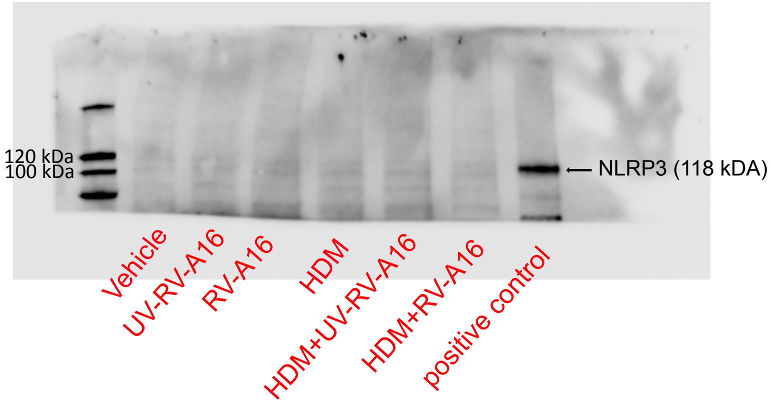

Asthma

Figure S4K

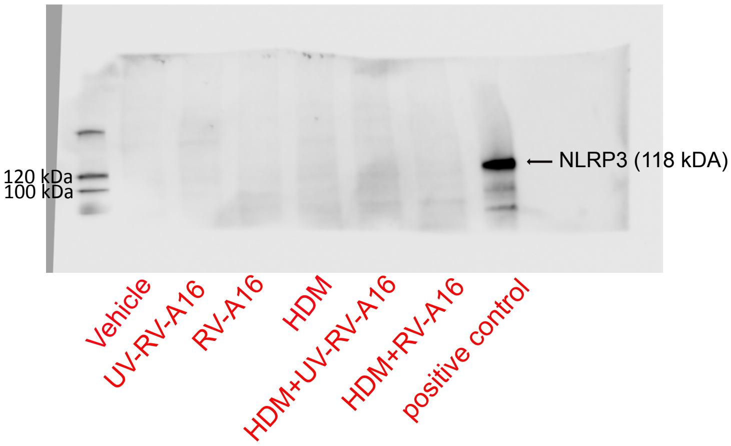

antibodies used:
mouse anti-NLRP3,
AG-20B-0014-C100, R&D systems

HRP goat anti-mouse IgG,
111-035-146, Jackson Laboratory

β-actin protein assessed in the cell lysates

Control

Figure S4K

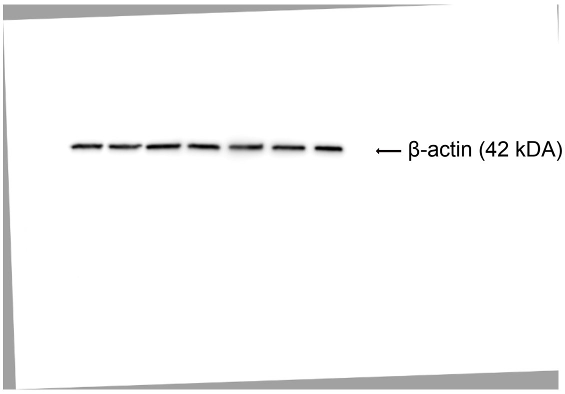

Asthma

Figure S4K

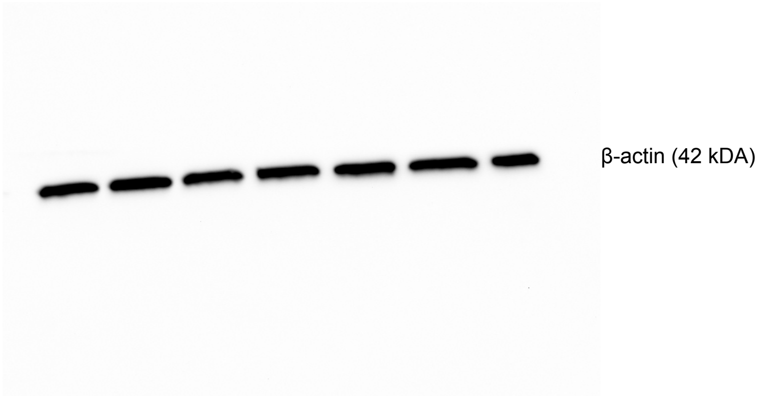

antibody used:
HRP anti-B-actin
ab49900, Abcam

Uncropped presentation of confocal pictures

Supplementary Figure 4I

Data presented: in vitro THP-1 cells

in vitro Isotype control

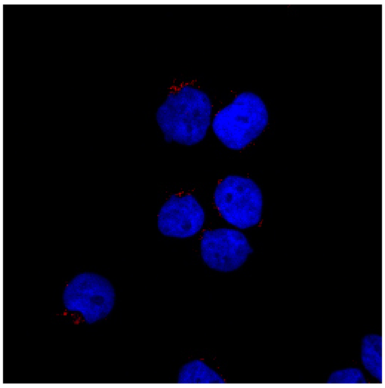

in vitro Medium control

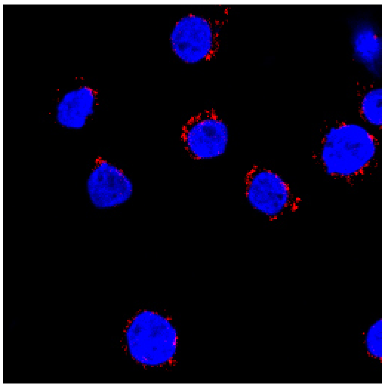

in vitro LPS+ATP

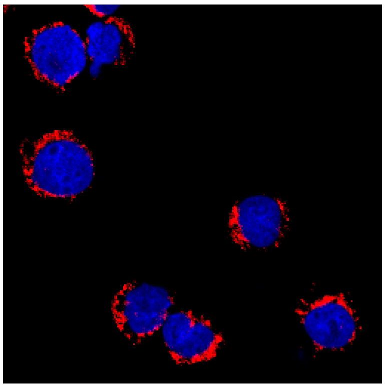

Supplement: Supplementary file 1 — Supplementary Information [file 41467_2023_37470_MOESM1_ESM.pdf]
