# Supplementary material for: Rhinovirus-induced epithelial RIG-I inflammasome suppresses antiviral immunity and promotes inflammation in asthma and COVID-19
Source: Nat Commun. 2023 Apr 22;14:2329. doi: 10.1038/s41467-023-37470-4 (PMC10122208; doi:10.1038/s41467-023-37470-4)
Supplement: Supplementary file 2 — Reporting Summary [file 41467_2023_37470_MOESM2_ESM.pdf]

nature portfolio

Corresponding author(s): Milena Sokolowska

Last updated by author(s): 2023_03_14

# Reporting Summary

Nature Portfolio wishes to improve the reproducibility of the work that we publish. This form provides structure for consistency and transparency in reporting. For further information on Nature Portfolio policies, see our Editorial Policies and the Editorial Policy Checklist.

## Statistics

For all statistical analyses, confirm that the following items are present in the figure legend, table legend, main text, or Methods section.

| n/a | Confirmed | |
|---|---|---|
| ☐ | ☒ | The exact sample size (*n*) for each experimental group/condition, given as a discrete number and unit of measurement |
| ☐ | ☒ | A statement on whether measurements were taken from distinct samples or whether the same sample was measured repeatedly |
| ☐ | ☒ | The statistical test(s) used AND whether they are one- or two-sided<br>*Only common tests should be described solely by name; describe more complex techniques in the Methods section.* |
| ☐ | ☒ | A description of all covariates tested |
| ☐ | ☒ | A description of any assumptions or corrections, such as tests of normality and adjustment for multiple comparisons |
| ☐ | ☒ | A full description of the statistical parameters including central tendency (e.g. means) or other basic estimates (e.g. regression coefficient) AND variation (e.g. standard deviation) or associated estimates of uncertainty (e.g. confidence intervals) |
| ☐ | ☒ | For null hypothesis testing, the test statistic (e.g. *F*, *t*, *r*) with confidence intervals, effect sizes, degrees of freedom and *P* value noted<br>*Give P values as exact values whenever suitable.* |
| ☒ | ☐ | For Bayesian analysis, information on the choice of priors and Markov chain Monte Carlo settings |
| ☐ | ☒ | For hierarchical and complex designs, identification of the appropriate level for tests and full reporting of outcomes |
| ☐ | ☒ | Estimates of effect sizes (e.g. Cohen's *d*, Pearson's *r*), indicating how they were calculated |

*Our web collection on statistics for biologists contains articles on many of the points above.*

## Software and code

Policy information about availability of computer code

Data collection

ELISA: Mithras LB940 (Berthold Technologies) and MicroWin 2010 version 5.25; MSD: MSD Discovery Workbench 4.0.12 (LSR_4_0_12); PEA: Biomark HD (Fluidigm) and Fluidigm Real Time PCR Analysis software version 4.5.2 + Olink NPX Manager version 3.0.1.389; WB: Luminescent Image Analyzed LAS-1000 (Fujifilm, Tokyo, Japan) or Fusion FX (Vilber) and Evolution Capt Edge version 18.11; RT-QPCR: quality of RNA was assessed by Nanodrop 2000 (ThermoFisher Scientific, Waltham, USA) and gene expression was measured by QuantStudio 7 Flex Real-Time PCR System (ThermoFisher Scientific) and QuantStudio Real-Time PCR Software version 1.5; Confocal: Zeiss LSM780 (Zeiss) and Zen Software version 3.2 Blue Edition; RNA-seq: Illumina HiSeq 4000 platform (Cohort A), Illumina HiSeq 2000 platform (GSE61141); Microarray: Transcriptome Analysis Console v4.0 (Santa Clara, United States).

Data analysis

Next generation sequencing data were processed with the workflow available at https://github.com/uzh/ezRun, with the significace threshold for differentially expressed genes set to p-value<.05 calculated for the entire gene lists in each project using the edgeR R package (version 3.16) (PMID: 19910308). Microarray data was analysed by the following Bioconductor microarray analysis workflow: https://www.bioconductor.org/packages/release/workflows/vignettes/arrays/inst/doc/arrays.html. Differentially expressed probe was identified by the limma R package with empirical Bayes estimation. Proximity Extension Assay (PEA) normalized protein expression (NPX) data were analyzed with the use of the internal Shiny App Olink data analysis toolkit. The statistical comparison of protein expression between groups was performed with the Bioconductor limma package (PMID: 25605792). The fold change and p-value were estimated by fitting a linear model for each protein. Proteins with p-value<0.05 were considered significant. Analyses made with Bioconductor version 3.16 and R version 4.2.0. Enriched pathways using Metacore software version 20.3.70200 or 19.2.69700 (Thomson Reuters). Protein interactions and pathways analysis prepared using the STRING (version 11.0 and 11.5), and further processed with the Cytoscape software (version 3.8.2 and 3.9.1). Protein interactions and pathways analysis for quantitative PEA were prepared using the STRING (version 11.5), and further processed with the Cytoscape software (version 3.9.1). Correlation between viral, RV-A16 and SARS-CoV-2 loads were calculated with Spearman`s rank correlation test. Power of the microarray analyses calculated by G*power 3.1 (https://www.psychologie.hhu.de/arbeitsgruppen/allgemeine-

psychologie-und-arbeitspsychologie/gpower) was sufficient to detect major differences. Western Blots were analysed with use of FIji (ImageJ) Software version 2.0.0-rc-49/1.51d. Statistical analysis was peformed with the Prism 9 software (Redmond, USA). The codes for transcriptome data analysis are available here: NGS [https://github.com/uzh/ezRun], microarray [https://github.com/ge11232002/p1688-Ula ]. Code for Proximity Extension Assay (PEA) data analysis is available here [https://github.com/ge11232002/OlinkR]. Confocal pictures were analyzed with use of Zen 3.2 Blue Edition Software (Zeiss, Oberkochen, Germany).

For manuscripts utilizing custom algorithms or software that are central to the research but not yet described in published literature, software must be made available to editors and reviewers. We strongly encourage code deposition in a community repository (e.g. GitHub). See the Nature Portfolio guidelines for submitting code & software for further information.

## Data

Policy information about availability of data

All manuscripts must include a data availability statement. This statement should provide the following information, where applicable:

- Accession codes, unique identifiers, or web links for publicly available datasets
- A description of any restrictions on data availability
- For clinical datasets or third party data, please ensure that the statement adheres to our policy

Transcriptome data from bronchial brushings from control individuals and patients with asthma infected in vivo with RV used in the study have been deposited in the NCBI GEO database and are available under accession number: GSE185658. All other data generated in the study are provided in Supplementary Information and Source data files. Publicly available RNAseq data GSE61141 were downloaded from the NCBI gene expression omnibus. Inflammasome-mediated immune response and antiviral response gene sets were curated from GSEA and MSigDB Database (Broad Institute, Massachusetts Institute of Technology, and Reagent of the University of California, USA). Full sets of analyzed genes are described in Supplementary Table 13.

## Human research participants

Policy information about studies involving human research participants and Sex and Gender in Research.

| | |
|---|---|
| Reporting on sex and gender | Information regarding sex and gender bias were not collected in this study. In the current manuscript we refer to the biological sex of the participants. |
| Population characteristics | Experimental in vivo rhinovirus infection population characteristics: Control individuals (n=9) mean age 31 years old, 66.7% male, no medicaments, mean FeV1 % predicted on day 0 - 101.4%, mean total IgE in serum - 17.44 IU/mL, no positive skin prick test; Patients with asthma (n=19) mean age 37.05 years old, 47.4% male, GINA status: 32% mild asthma and 68% moderate asthma, ACQ status at baseline: 37% well controlled asthma, 31% partially controlled asthma, 32% poor asthma control; 58% patients received ICS+SABA and 42% received only SABA treatment, mean FeV1 % predicted on day 0 - 83.53%, mean total IgE in serum - 453.7 IU/mL, mean total skin prick test weal size - 11.89 mm, and mean house dust mite skin prick test weal size - 11.89 mm. Characteristics of participants from cohort SIBRO: Control individuals (n=7) median age 43 years, 57% male and median BMI 22.86 kg/m2; Patients with asthma (n=12) median age 45 years, 33% male and median BMI 22.26 kg/m2. Characteristics of participants from cohort A: Control individuals (n=5), median age 35 years old, 60% male; patients with asthma (n=12), median age 51.5 years, 33% male. These data are specified in the Supplementary Table 1, 9. |
| Recruitment | Experimental in vivo rhinovirus infection in 11 control individuals and 28 patients with asthma was performed as reported previously (PMID: 25350863). Briefly, non-smoking, non-atopic control individuals, and non-smoking mild/moderate patients with asthma without any recent viral illness and without serum neutralizing antibodies towards RV-A16, who passed inclusion criteria, underwent infection on day 0 with RV-A16 at the dose of 100 TCID50. Bronchial brushings, bronchial biopsies and bronchoalveolar lavage (BAL) fluid were collected around 2 weeks before and at 4 days after RV-A16 infection. Additionally, nasal lavage (NL) samples at the peak of RV-A16 infection were collected to assess RV-A16 infection rates. Only subjects who had sufficient remaining samples to be analyzed in this original study and/or subjects who had a successful infection in the lungs, as assessed by viral RNA copies by qPCR, were included in the BAL, NL, and biopsies analyses (n=9 healthy control, n=19 patients with asthma), and bronchial brushing microarray analysis (n=7 healthy controls, n=17 patients with asthma). The study received ethical approval from the St. Mary`s Hospital Research Ethics Committee (09/H0712/59). The clinical characteristics of the 9 control and 19 asthma study participants who had sufficient remaining samples to be analyzed in this study is presented in Supplementary Tables 1 and 9. Additionally, all details regarding the cohort are disclosed in Supplementary Table 10. This observational cohort was registered at clinicaltrials.gov under the identifier NCT01159782. Control individuals and patients with asthma were enrolled in the ALL-MED Medical Research Institute, Wroclaw, Poland; the Pulmonary Division, University Hospital of Zurich, Switzerland (cohort SIBRO), or at the University Hospital, Jagiellonian University Medical College, Cracow, Poland (cohort A). Briefly, bronchoscopy with epithelial cells brushings and BAL fluid collection was performed. The studies was granted ethical permission from Switzerland and Poland (KEK-ZH-Nr. 20212-0043 – Kantonale Ethik-Kommission Zürich; KB-70/2013 and KB-567/2014 – Bioethical Committee, Wroclaw Medical University) or the Jagiellonian University Bioethics Committee (KBET/68/B/2008 and KBET/209/B/2011). The detailed description of patients recruitment is available in the original papers for: experimental in vivo rhinovirus infection (PMID: 25350863), cohort SIBRO (PMID: 31836714) and cohort A (PMID: 27312821). Potential biases during the recruitment process are as follows: in vivo RV-A16 cohort included only patients with mild and moderate asthma, and due to the ethical concerns those with severe asthma were excluded. We analyzed only patients with succesful RV-A16 infection detected in the BAL fluid, which has sufficient remaining samples in the biobank. |
| Ethics oversight | The in vivo experimental infection with rhinovirus study received ethical approval from the St. Mary`s Hospital Research Ethics Committee (09/H0712/59). This observational cohort was registered at clinicaltrials.gov under the identifier NCT01159782. All participants gave written, informed consent. Further use and additional analyses, including RNA microarray, were permitted and consented. The cohort SIBRO was granted ethical permission from Switzerland and Poland (KEK-ZH-Nr. 20212-0043 – Kantonale Ethik-Kommission Zürich; KB-70/2013 and KB-567/2014 – Bioethical Committee, |

Wroclaw Medical University). All participants gave written, informed consent. Further use and additional analyses were permitted and consented. The cohort A got a permission from the Jagiellonian University Bioethics Committee (KBET/68/B/2008 and KBET/209/B/2011). All participants gave written, informed consent. Further use and additional analyses, including NGS, were permitted and consented.

Note that full information on the approval of the study protocol must also be provided in the manuscript.

# Field-specific reporting

Please select the one below that is the best fit for your research. If you are not sure, read the appropriate sections before making your selection.

- [x] Life sciences
- [ ] Behavioural & social sciences
- [ ] Ecological, evolutionary & environmental sciences

For a reference copy of the document with all sections, see nature.com/documents/nr-reporting-summary-flat.pdf

# Life sciences study design

All studies must disclose on these points even when the disclosure is negative.

| | |
|---|---|
| Sample size | Primary bronchial epithelial cells sample size is based on preliminary results and available literature. In human cohorts sample sizes were dictated by the number of study participants and samples availability. Conclusions in the manuscript are supported by appropriate statistical tests, and all data points are shown. |
| Data exclusions | In experimental cohort of in vivo human rhinovirus infection available samples from individuals not demonstrating infection in the lungs were excluded. Otherwise no data were excluded. |
| Replication | Data delivered from the experimental in vivo RV-A16 infection in healthy controls and patients with asthma, performed only once by Jackson et al. (PMID: 25350863) indicates the number of biologically independent samples examined over one experimental infection with RV-A16. The number of biological samples is stated in each figure legend. Biological replicates guarantee reproducibility of the data. In vitro experiments were successfully and independently reproduced at least three times (two for SARS-CoV-2 infection in vitro) in several biological replicates of primary cells from patients with asthma and healthy individuals. The number of biological replicates for all analyzes is described in detail in figure legends and source data files. |
| Randomization | The in vivo studies and in vitro experiments were not randomized. |
| Blinding | Due to the character of the in vivo RV-A16 infection study (diagnosis, inclusion/exclusion criteria for RV-A16 infection) investigators were not blinded for patients allocation. Blinding was not relevant for this study, as clinical diagnosis (asthma vs control group) and before-after read-outs for RV-A16 infection were considered as primary outcomes. In vitro studies were not blinded. In vitro experiments blinding was not relevant for this study, as clinical diagnosis (asthma vs control group) and before-after read-outs for multiple stimulations were considered as primary outcomes. Quantification of in vitro Western Blots and confocal pictures were performed in a blinded manner. |

# Reporting for specific materials, systems and methods

We require information from authors about some types of materials, experimental systems and methods used in many studies. Here, indicate whether each material, system or method listed is relevant to your study. If you are not sure if a list item applies to your research, read the appropriate section before selecting a response.

## Materials & experimental systems

| n/a | Involved in the study | |
|---|---|---|
| [ ] | [x] | Antibodies |
| [ ] | [x] | Eukaryotic cell lines |
| [x] | [ ] | Palaeontology and archaeology |
| [x] | [ ] | Animals and other organisms |
| [x] | [ ] | Clinical data |
| [x] | [ ] | Dual use research of concern |

## Methods

| n/a | Involved in the study | |
|---|---|---|
| [x] | [ ] | ChIP-seq |
| [x] | [ ] | Flow cytometry |
| [x] | [ ] | MRI-based neuroimaging |

## Antibodies

| | |
|---|---|
| Antibodies used | Human IL1B/IL-1F2 duo set, cat. no. DY201 (R&D systems); V-Plex Human IL1B kit, cat. no. K151QPD-1, (MSD); for WB analyses: Mouse anti-NLRP3, cat. no. AG-20B-0014-C100, (Adipogen); Goat anti-IL1B, cat. no. AF-201-NA, (R&D systems); Mouse anti-ASC, cat. no. sc-514414, (Santa Cruz Biotechnology); Rabbit anti-Caspase-1, cat. no. 2225, (Cell Signaling); Goat anti-RIG-I, cat. no. sc-48929, (Santa Cruz Biotechnology); HRP Goat Anti Mouse IgG, cat. no. 111-035-146, (Jackson Laboratory); HRP Mouse anti-goat IgG, cat. no. sc2354, (Santa Cruz Biotechnology); HRP AffiniPure Goat Anti Rabbit IgG, cat. no. 111-035-003, (Jackson Laboratory); HRP Anti-beta Actin, cat. no. ab49900, (Abcam), Direct-Blot HRP anti-beta-actin, cat no. 664803 (Biolegend). For co-immunoprecypitation: Rabbit anti-ASC, cat. no. sc22514-R, (Santa Cruz Biotechnology); Mouse anti-RIG-I, cat. no. sc376845, (Santa Cruz Biotechnology); HRP Goat |

Anti Mouse IgG, cat. no. 115-035-146, (Jackson Laboratory), Rabbit anti-MDA5, cat. No. ab126630, (Abcam);HRP AffiniPure Goat Anti Rabbit IgG, cat. No. 111-035-003, (Jackson Laboratory); WB from apical compartments: Goat anti-IL1B, cat. no. AF-201-NA, (R&D systems); HRP Mouse anti-goat IgG, cat. no. sc2354, (Santa Cruz Biotechnology); Confocal staining in i) human bronchial epithelial cells: Mouse IgG1 anti-IL1B, cat. no. ab156791, (Abcam); Mouse IgG1 anti-RIG-I, cat. no. sc-376845, (Santa Cruz Biotechnology); Mouse IgG1 anti-ASC, cat. no. sc-514414, (Santa Cruz Biotechnology); Goat anti-mouse IgG, Alexa Fluor 546, cat. no. A11003, (Invitrogen); Goat anti-mouse IgG, Alexa Fluor 488, cat. no. A11001, (Invitrogen); ii) bronchial biopsies: Rabbit anti-Caspase-1, cat. no. 2225, (Cell Signalling); Mouse IgG1 anti-IL1B, cat. no. ab156791, (Abcam); Mouse IgG1 anti-RIG-I, cat. no. sc-376845, (Santa Cruz Biotechnology); Goat anti-Rabbit IgG, Alexa Fluor 488, cat. no. (A11034), Invitrogen; Goat anti-mouse IgG, Alexa Fluor 546, cat. no. A11003, (Invitrogen); iii) NLRP3 and for SARS-CoV-2 experiment: Anti ACE2, cat. no. ab15348, (Abcam); Anti NLRP3, cat. no. AG-20B-0014-C100, (Adipogen); Anti Occludin, cat. no. OC-3F10, (ThermoFisher); Anti N-Protein, cat. no. MA1-7404, (ThermoFisher); Goat anti-Rabbit IgG Alexa 546, cat. no. A11010, (Invitrogen); Goat anti mouse IgG2b, cat. no. (A21143), Invitrogen.

Validation

All commercially available antibodies were validated by manufacturer. Additional information can be obtained on the company websites provided below:
WB analyses: Mouse anti-NLRP3, cat. no. AG-20B-0014-C100, (Adipogen), https://adipogen.com/ag-20b-0014-anti-nlrp3-nalp3-mab-cryo-2.html/; Goat anti-IL1B, cat. no. AF-201-NA, (R&D systems), https://www.rndsystems.com/products/human-il-1beta-il-1f2-antibody_af-201-na; Mouse anti-ASC, cat. no. sc-514414, (Santa Cruz Biotechnology), https://www.scbt.com/p/asc-antibody-b-3; Rabbit anti-Caspase-1, cat. no. 2225, (Cell Signaling), https://www.cellsignal.com/products/primary-antibodies/caspase-1-antibody/2225; Goat anti-RIG-I, cat. no. sc-48929, (Santa Cruz Biotechnology), https://www.scbt.com/p/rig-i-antibody-c-15, product discontinued; For co-immunoprecypitation: Rabbit anti-ASC, cat. no. sc22514-R, (Santa Cruz Biotechnology), https://www.scbt.com/p/asc-antibody-n-15, product discontinued; Mouse anti-RIG-I, cat. no. sc376845, (Santa Cruz Biotechnology), https://www.scbt.com/p/rig-i-antibody-d-12; Rabbit anti-MDA5, cat. No. ab126630, (Abcam), https://www.abcam.com/nav/primary-antibodies/rabbit-monoclonal-antibodies/mda5-antibody-epr6743-ab126630.html; WB from apical compartments: Goat anti-IL1B, cat. no. AF-201-NA, (R&D systems), https://www.rndsystems.com/products/human-il-1beta-il-1f2-antibody_af-201-na; Confocal staining in i) human bronchial epithelial cells: Mouse IgG1 anti-IL1B, cat. no. ab156791, (Abcam); https://www.abcam.com/il-1-beta-antibody-oti3e1-ab156791.html; Mouse IgG1 anti-RIG-I, cat. no. sc-376845, (Santa Cruz Biotechnology); https://www.scbt.com/p/rig-i-antibody-d-12; Mouse IgG1 anti-ASC, cat. no. sc-514414, (Santa Cruz Biotechnology); https://www.scbt.com/p/asc-antibody-b-3?gclid=CjwKCAiAm7OMBhAQEiwArvGi3I270DjHRSaBvswQnXksHQX6FsJMwG5A_8UEtzZdL3ul0rG7_ASjnBoCRq4QAvD_BwE; ii) bronchial biopsies: Rabbit anti-Caspase-1, cat. no. 2225, (Cell Signalling); https://www.cellsignal.com/products/primary-antibodies/caspase-1-antibody/2225; Mouse IgG1 anti-IL1B, cat. no. ab156791, (Abcam); https://www.abcam.com/il-1-beta-antibody-oti3e1-ab156791.html; Mouse IgG1 anti-RIG-I, cat. no. sc-376845, (Santa Cruz Biotechnology); https://www.scbt.com/p/rig-i-antibody-d-12; iii) NLRP3 and for SARS-CoV-2 experiment: Anti ACE2, cat. no. ab15348, (Abcam); https://www.abcam.com/ace2-antibody-ab15348.html; Anti NLRP3, cat. no. AG-20B-0014-C100, (Adipogen); https://adipogen.com/ag-20b-0014-anti-nlrp3-nalp3-mab-cryo-2.html; Anti Occludin, cat. no. OC-3F10, (ThermoFisher); https://www.thermofisher.com/antibody/product/33-1500.html?ef_id=CjwKCAiAm7OMBhAQEiwArvGi3FySohOszNZuTdvSxYBIfOtDzkiBcx9yhJh73uAz3vJckJkAgJVn8hoCNu4QAvD_BwE:G:s&s_kwcid=AL!3652!3!459736943987!!!g!!
&cid=bid_pca_aup_r01_co_cp1359_pjt0000_bid00000_0se_gaw_dy_pur_con&gclid=CjwKCAiAm7OMBhAQEiwArvGi3FySohOszNZuTdvSxYBIfOtDzkiBcx9yhJh73uAz3vJckJkAgJVn8hoCNu4QAvD_BwE; Anti N-Protein, cat. no. MA1-7404, (ThermoFisher), https://www.thermofisher.com/antibody/product/SARS-SARS-CoV-2-Coronavirus-Nucleocapsid-Antibody-clone-B46F-Monoclonal/MA1-7404. Mouse IgG2a monoclonal anti-human ICAM-1 antibody R6.5 was produced in hybridoma cells as shown previously (DOI:10.1111/all.12931), and validation was based on functional assays: limiting rhinovirus A16 infection.

## Eukaryotic cell lines

Policy information about cell lines and Sex and Gender in Research

Cell line source(s)

Primary Bronchial Epithelial cells were purchased from Lonza (CC-2540) and Epithelix (EP51AB). THP-1 cell line (thpx-sp) was purchased from Invivogen. Mouse hybridoma cells (HB-9580), HeLa, Vero E6, and BHK-21 cell lines were purchased from ATCC.

Authentication

Epithelial cells used in the manuscript are primary bronchial epithelial cells isolated from human bronchial biopsies, authenticated and performance-assayed by the vendor. THP-1, HB-9580, HeLa, Vero E6, and BHK-21 cell lines were profiled by the vendor. Primary epithelial cells obtained from the biopsies from patients and healthy controls from cohort SIBRO and cohort A were authenticated by the cellular markers by PCR assays with species-specific primers. (PMID: 23590309, PMID: 36642382)

Mycoplasma contamination

Cells tested negative for mycoplasma contamination.

Commonly misidentified lines
(See ICLAC register)

No commonly misidentified lines were used.
