# Supplementary material for: Rhinovirus-induced epithelial RIG-I inflammasome suppresses antiviral immunity and promotes inflammation in asthma and COVID-19
Source: Nat Commun. 2023 Apr 22;14:2329. doi: 10.1038/s41467-023-37470-4 (PMC10122208; doi:10.1038/s41467-023-37470-4)

Uncropped presentation of Western Blots

IL-1β protein assessed in the supernatants

Control

Asthma

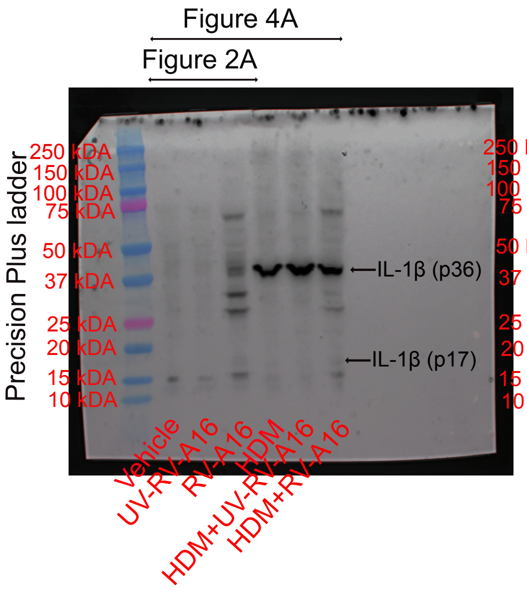

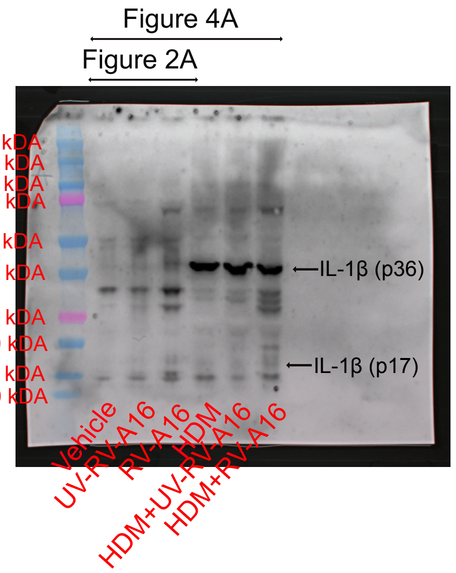

antibodies used:
goat anti-IL-1β,
AF-201-NA, Abcam

HRP mouse antigoat IgG,
sc2354, Santa Cruz Biotechnology

IL-1β protein assessed in the cell lysates

Control

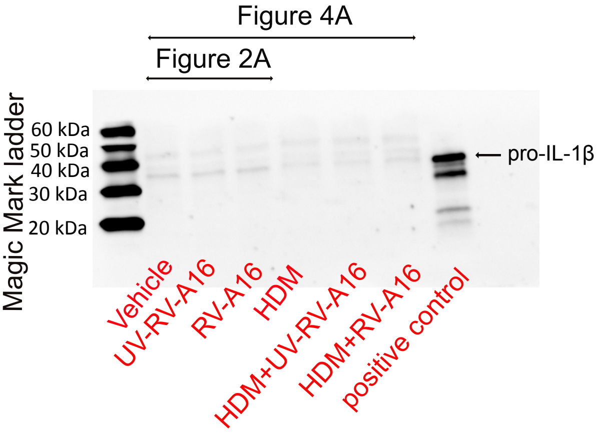

Asthma

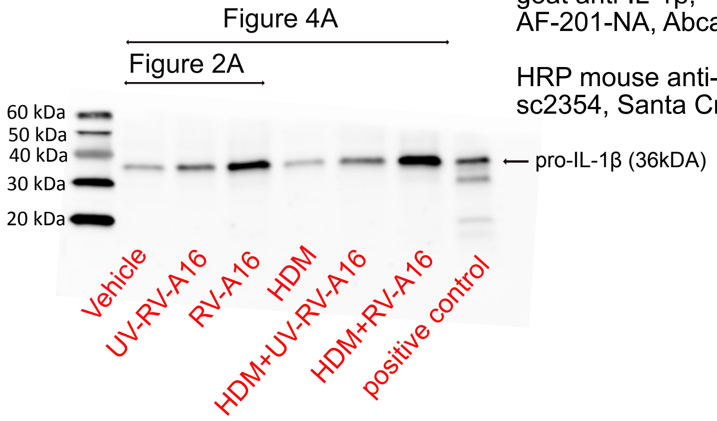

antibodies used:
goat anti-IL-1β,
AF-201-NA, Abcam

HRP mouse anti-goat IgG,
sc2354, Santa Cruz Biotechnology

ASC protein assessed in the cell lysates

Control

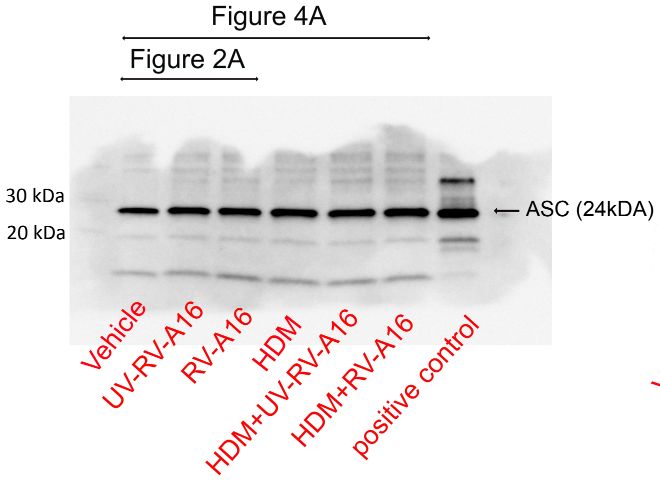

Asthma

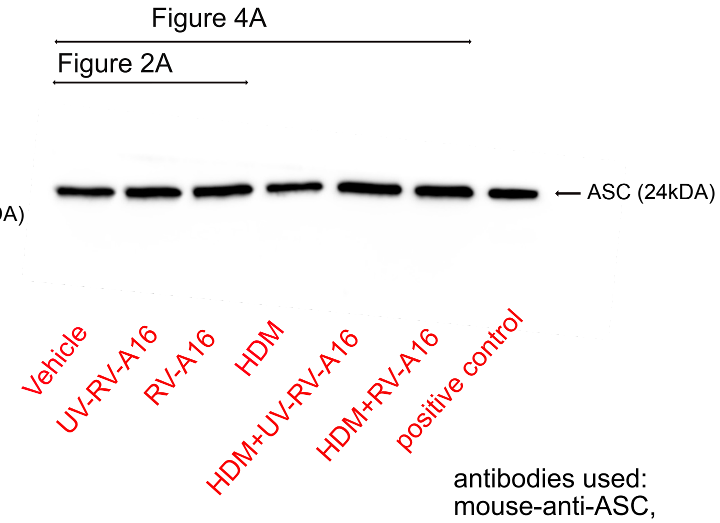

antibodies used:
mouse-anti-ASC,
sc-514414, Santa Cruz Biotechnology

HRP goat anti-mouse IgG,
111-035-146, Jackson Laboratory

Caspase-1 protein assessed in the cell lysates

Control

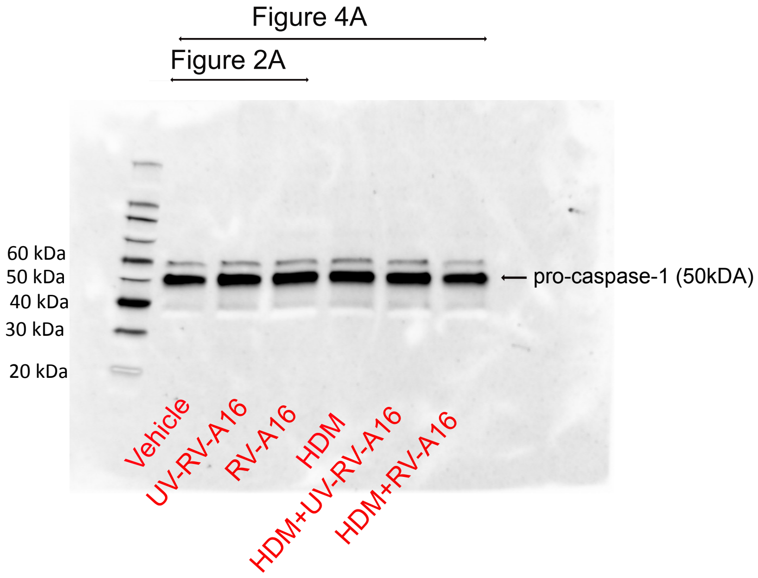

Asthma

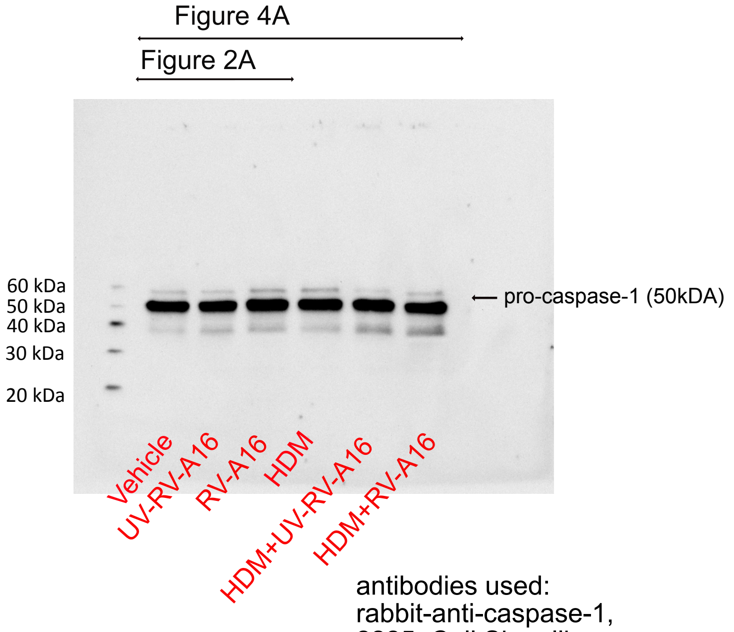

antibodies used:
rabbit-anti-caspase-1,
2225, Cell Signalling

HRP Affinity Pure Goat anti-rabbit IgG,
111-035-003, Jackson Laboratory

β-actin protein assessed in the cell lysates

Control

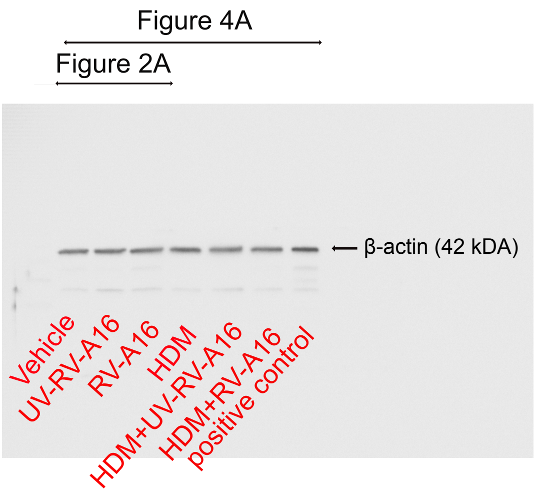

Asthma

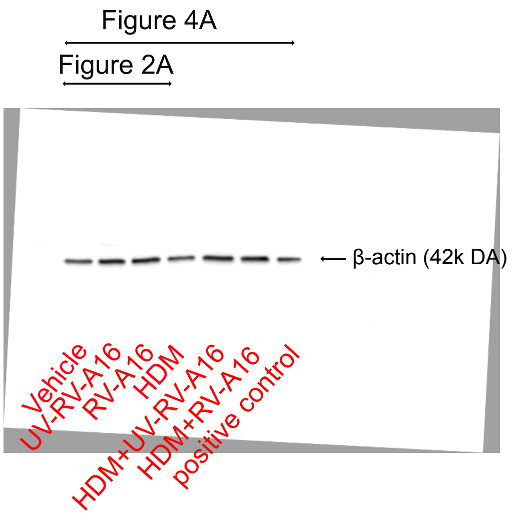

antibody used:
HRP anti-B-actin
ab49900, Abcam

RIG-I protein assessed in the cell lysates

Asthma

Figure 2H

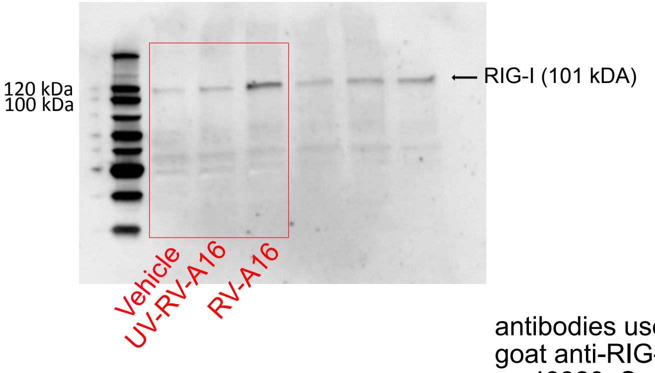

antibodies used:
goat anti-RIG-I,
sc-48929, Santa Cruz Biotechnology

HRP mouse anti-Goat IgG,
sc2354, Santa Cruz Biotechnology

β-actin protein assessed in the cell lysates

Asthma

Figure 2H

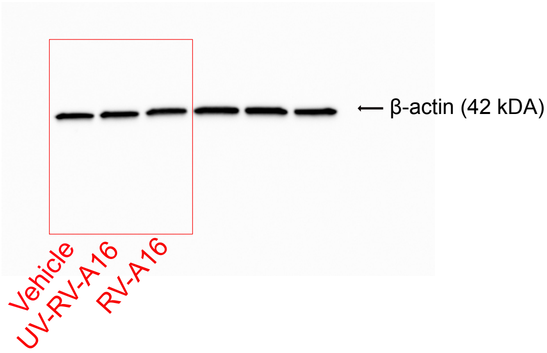

antibody used:
HRP anti-B-actin
ab49900, Abcam

NLRP3 protein assessed in the cell lysates

Asthma

Figure 2L

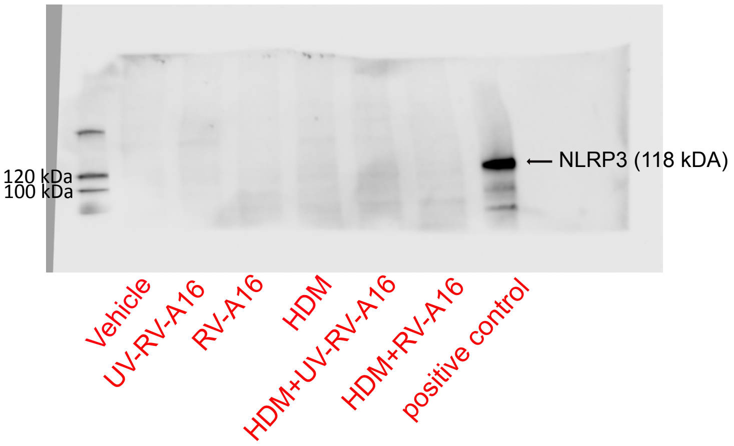

antibodies used:
mouse anti-NLRP3,
AG-20B-0014-C100, R&D systems

HRP goat anti-mouse IgG,
111-035-146, Jackson Laboratory

β-actin protein assessed in the cell lysates

Asthma

Figure 2L

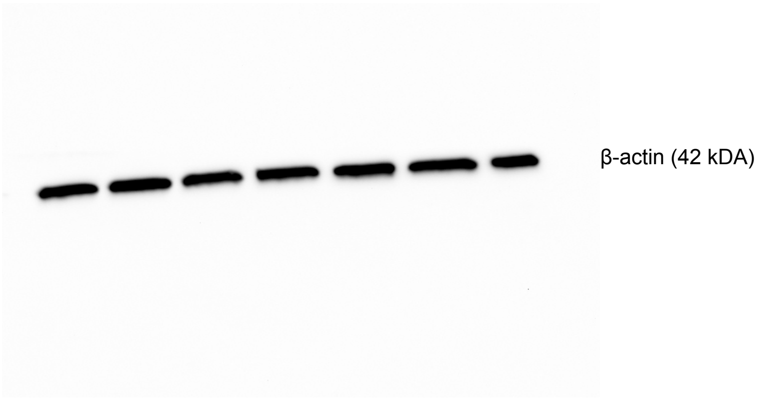

antibody used:
HRP anti-B-actin
ab49900, Abcam

ASC and RIG-I co-immunoprecipitation

Asthma

Figure 2J

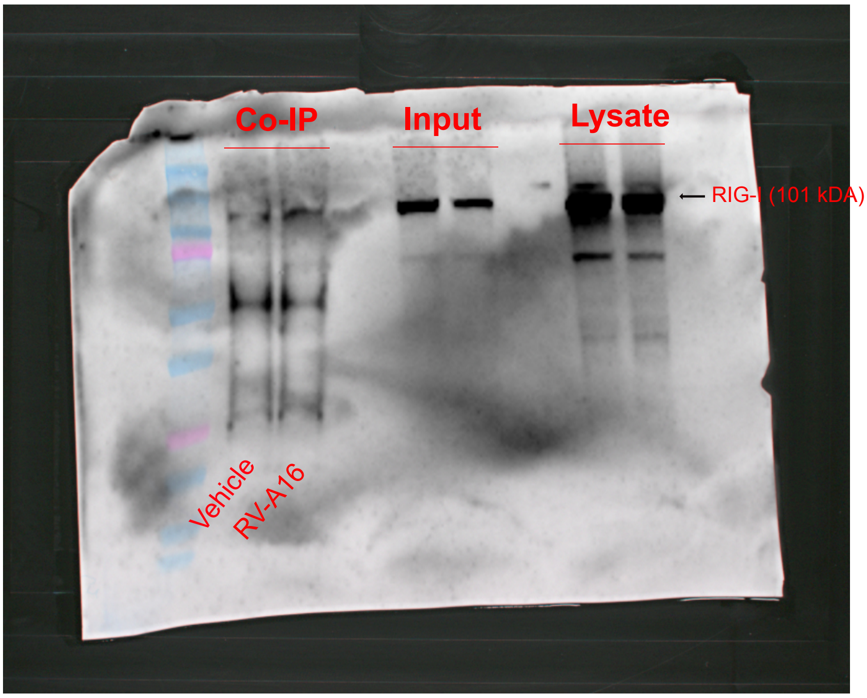

antibodies used:
co-IP: rabbit anti-ASC
sc22514-R, Santa Cruz Biotechnology

WB:
Mouse anti-RIG-I
sc376845, Santa Cruz Biotechnology

HRP goat anti-mouse IgG,
111-035-146, Jackson Laboratory

ASC and MDA5 co-immunoprecipitation

Asthma

Figure 2J

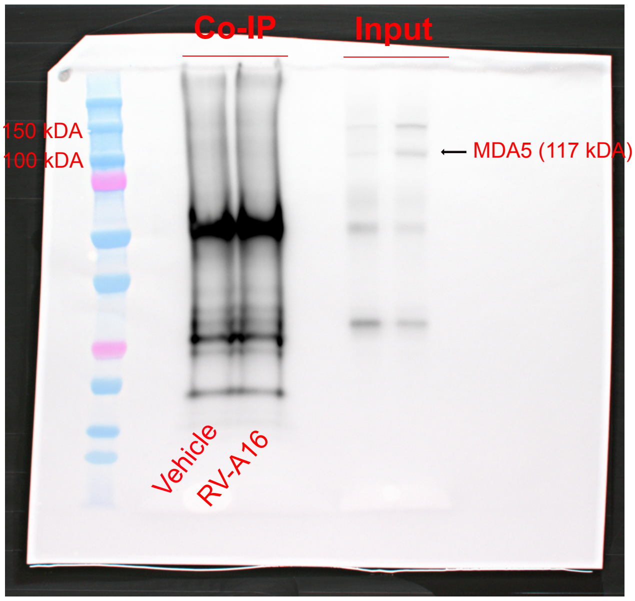

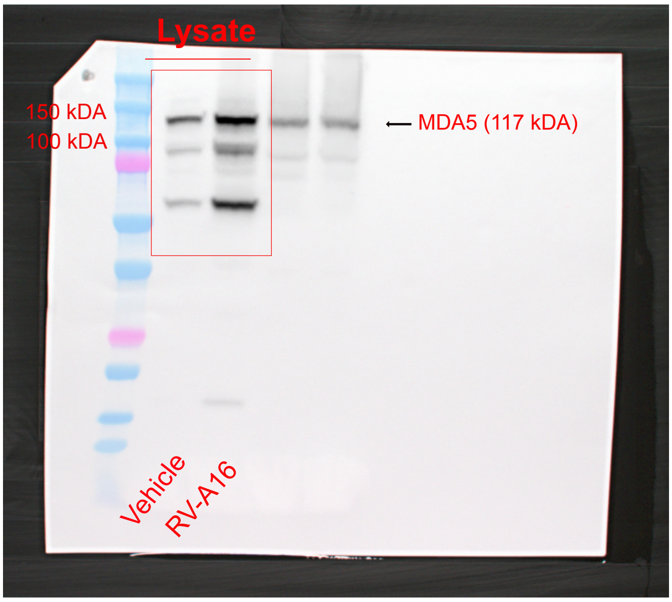

co-IP: antibodies used:
rabbit anti-ASC
sc22514-R, Santa Cruz Biotechnology

WB:
Rabbit anti-MDA5
ab126630, Abcam

HRP AffiniPure Goat anti-rabbit IgG
111-035-003, Jackson Laboratory

IL-1β protein assessed in the supernatants

Control

Figure 4G

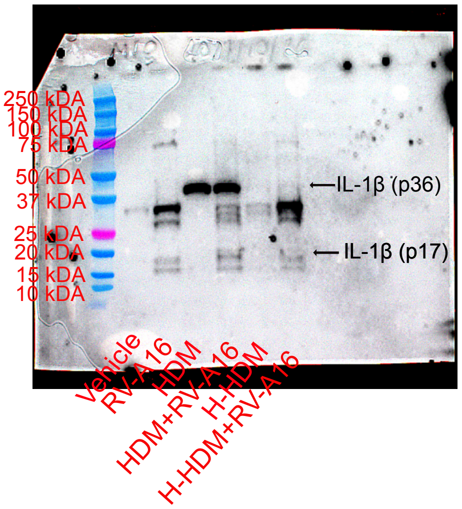

Asthma

Figure 4G

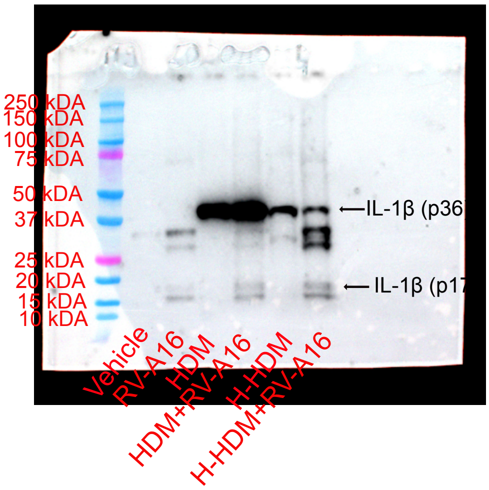

antibodies used:
goat anti-IL-1β,
AF-201-NA, Abcam

HRP mouse anti-goat IgG,
sc2354, Santa Cruz Biotechnology

IL-1β protein assessed in the cell lysates

Control

Figure 4G

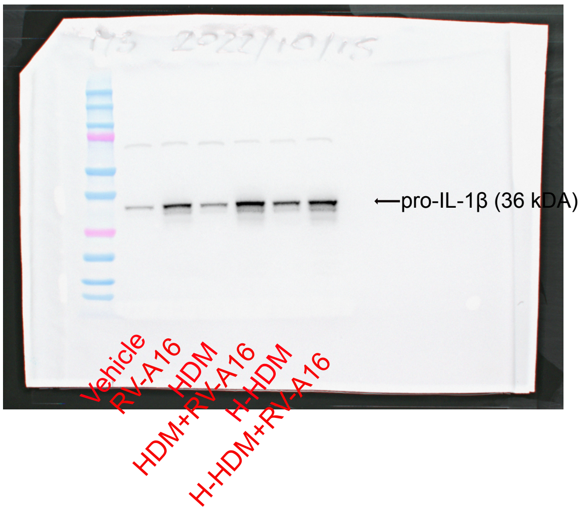

Asthma

Figure 4G

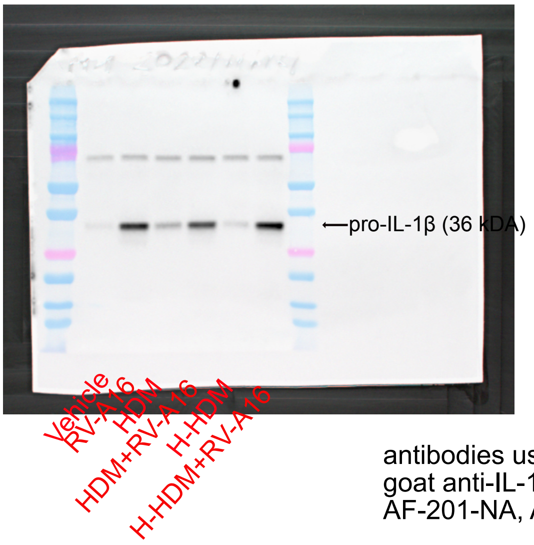

antibodies used:
goat anti-IL-1β,
AF-201-NA, Abcam

HRP mouse antigoat IgG,
sc2354, Santa Cruz Biotechnology

β-actin protein assessed in the cell lysates

Control

Figure 4G

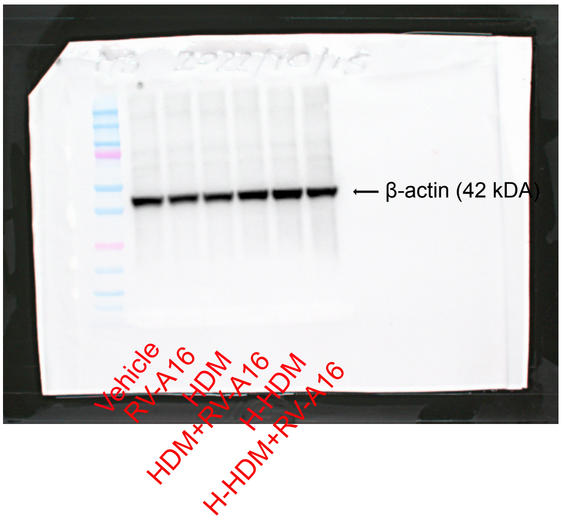

Asthma

Figure 4G

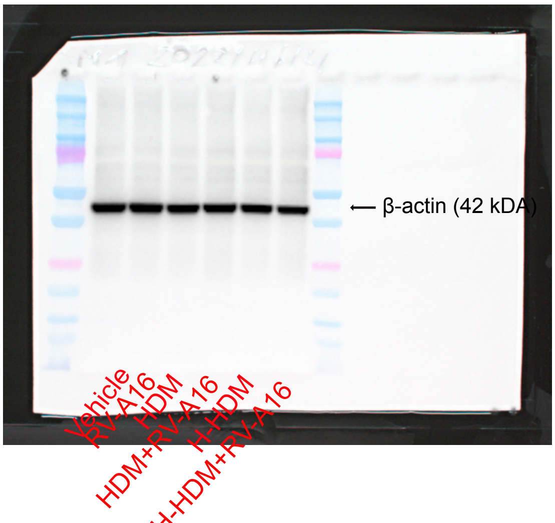

antibody used:
Direct-Blot HRP anti-B-actin
664803, Biolegend

IL-1β protein assessed in the supernatants

Control

Figure 4I

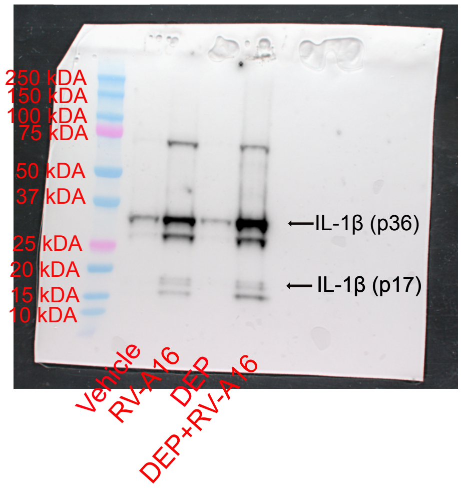

Asthma

Figure 4I

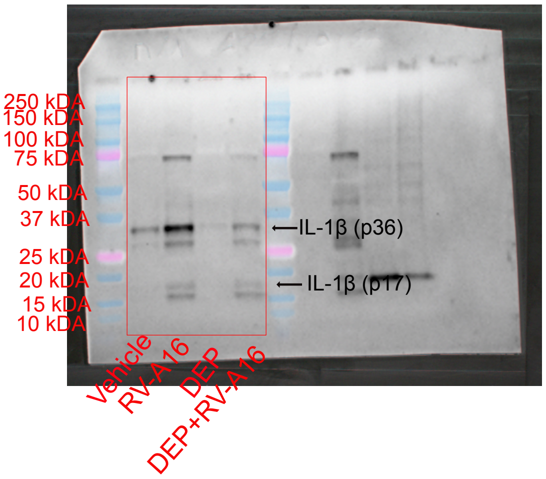

antibodies used:
goat anti-IL-1β,
AF-201-NA, Abcam

HRP mouse anti-goat IgG,
sc2354, Santa Cruz Biotechnology

IL-1β protein assessed in the cell lysates

Control

Figure 4I

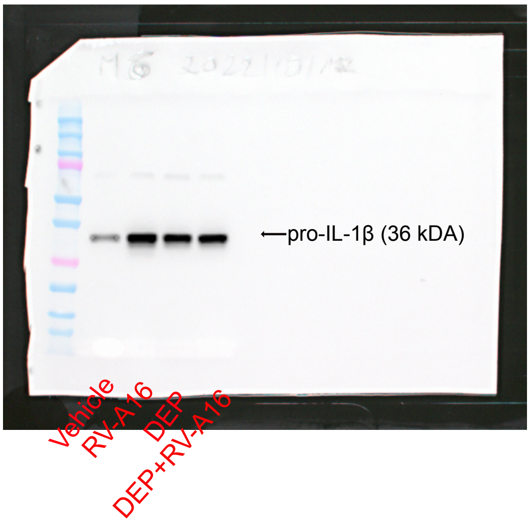

Asthma

antibodies used:
goat anti-IL-1β,
AF-201-NA, Abcam

HRP mouse anti-goat IgG,
sc2354, Santa Cruz Biotechnology

Figure 4I

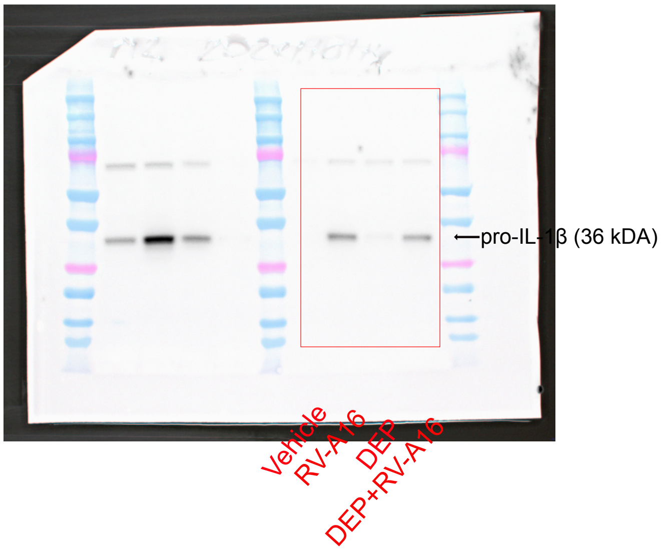

β-actin protein assessed in the cell lysates

Control

Figure 4I

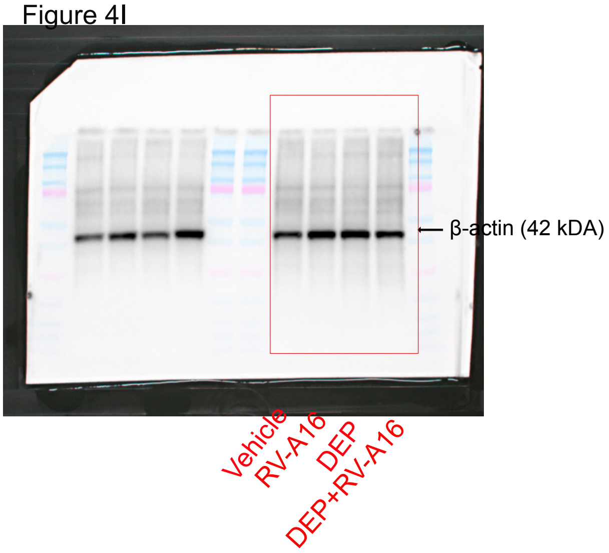

Asthma

Figure 4I

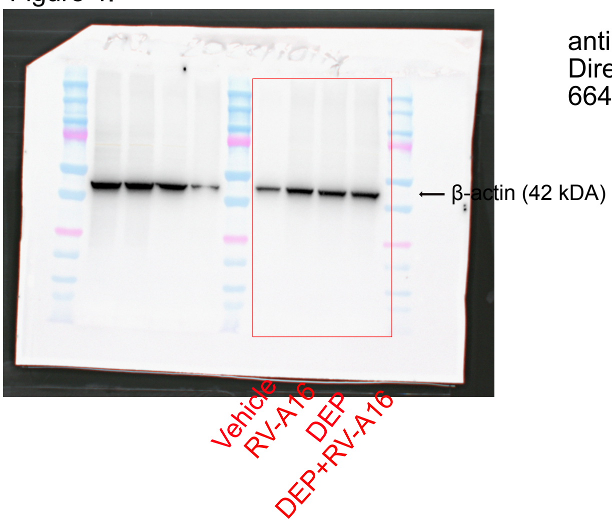

antibody used:
Direct-Blot HRP anti-B-actin
664803, Biolegend

Uncropped presentation of confiocal pictures

Figure 1e

Data presented: in vivo experimental RV-A16 infection in humans

Control before in vivo RV-A16 infection

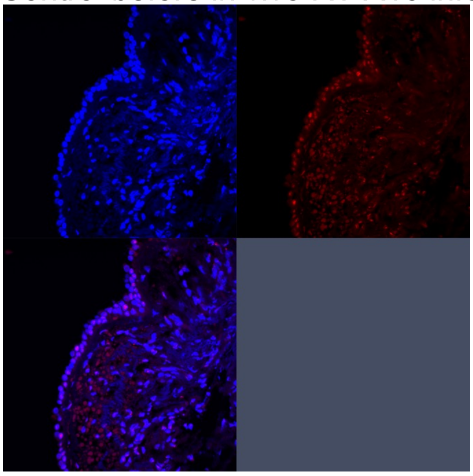

Control after in vivo RV-A16 infection

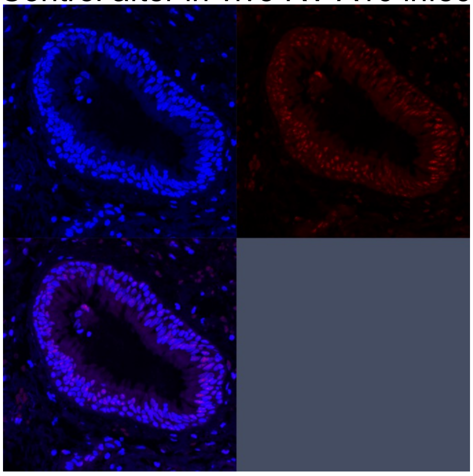

Asthma before in vivo RV-A16 infection

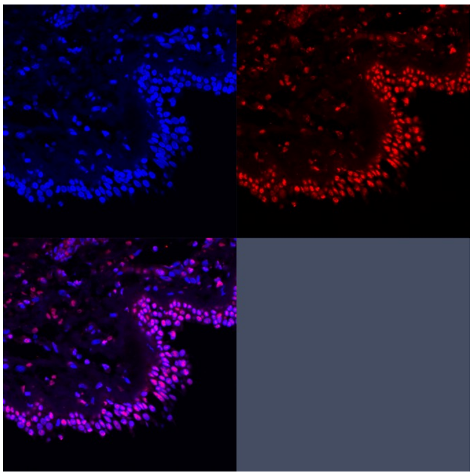

Asthma after in vivo RV-A16 infection

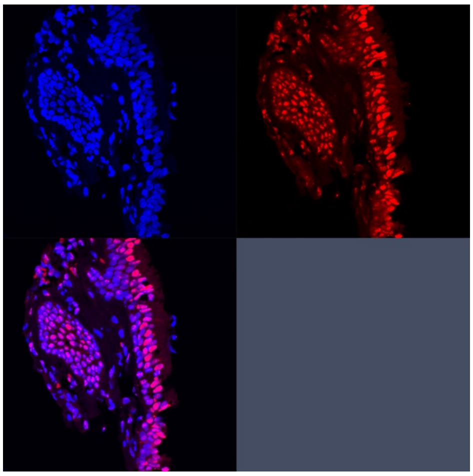

Figure 1j

Data presented: in vivo experimental RV-A16 infection in humans

Control before in vivo RV-A16 infection

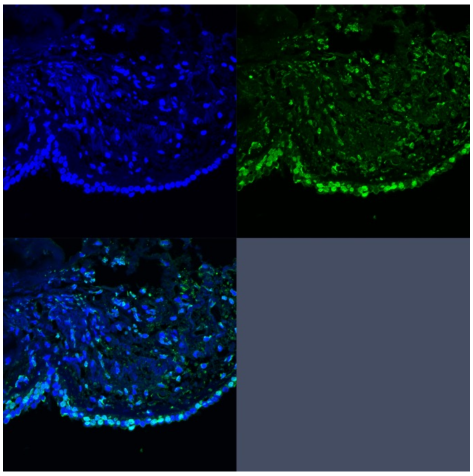

Control after in vivo RV-A16 infection

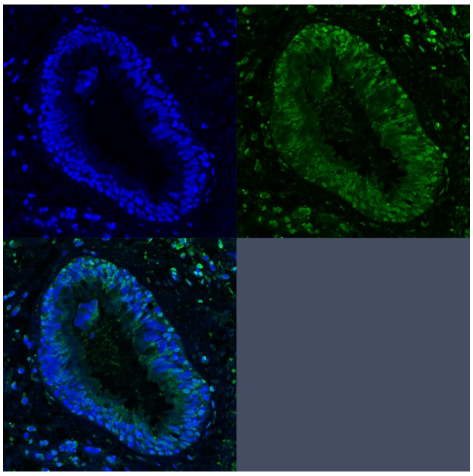

Asthma before in vivo RV-A16 infection

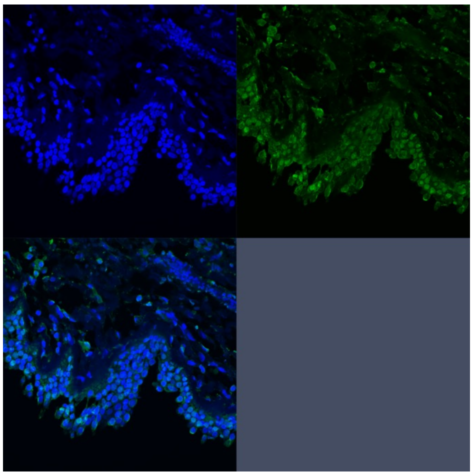

Asthma after in vivo RV-A16 infection

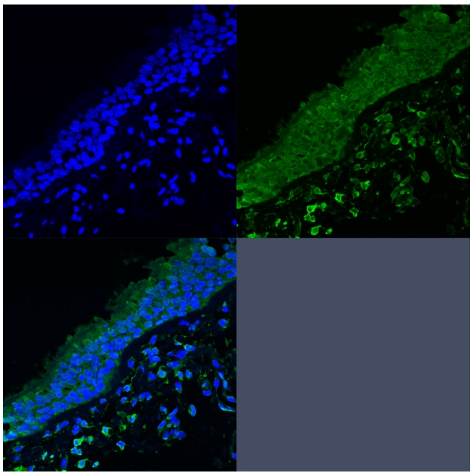

Figure 2c

Data presented: in vitro RV-A16 infection in air-liquid-differentiated primary human bronchial epithelial cells

in vitro Isotype control

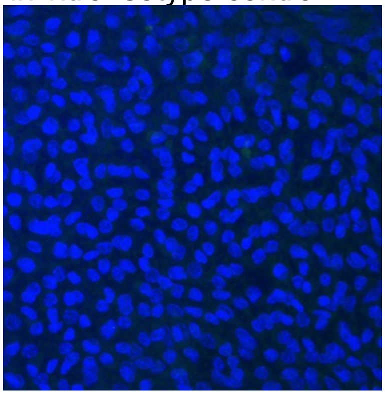

Control
in vitro Medium control

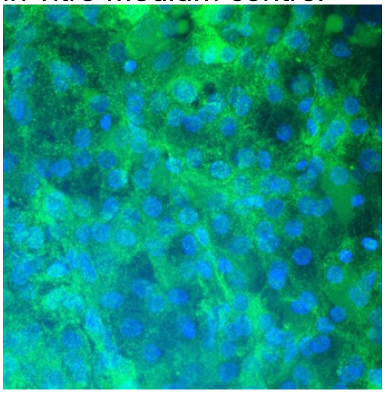

Asthma
in vitro Medium control

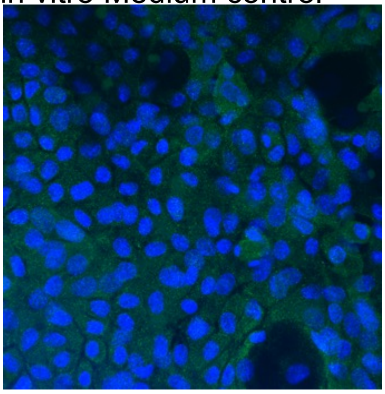

in vitro UV-RV-A16

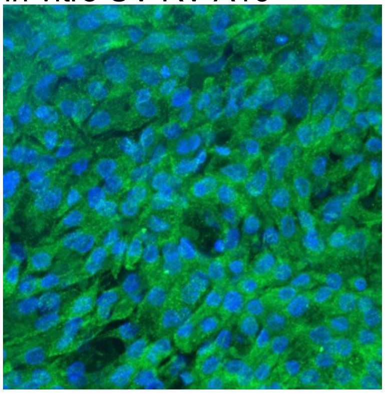

in vitro UV-RV-A16

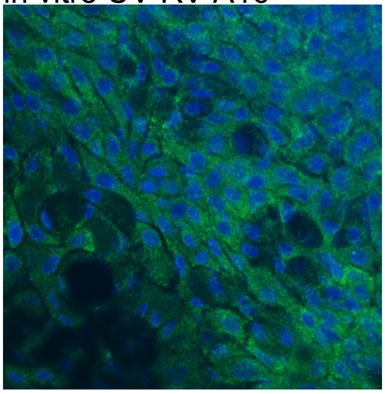

in vitro RV-A16

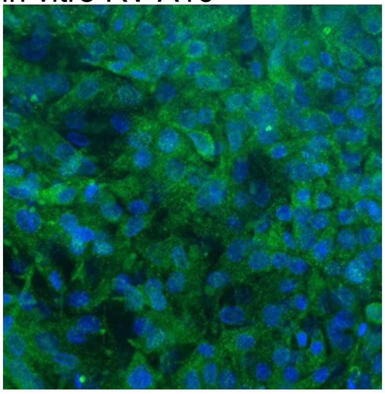

in vitro RV-A16

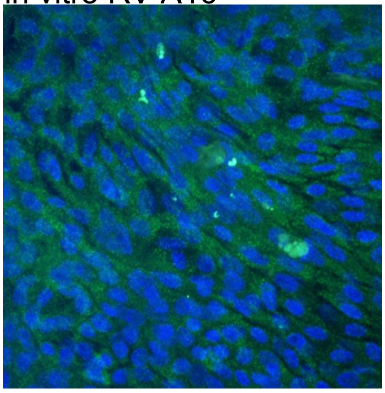

Figure 2i

Data presented: in vitro RV-A16 infection in air-liquid-differentiated primary human bronchial epithelial cells

in vitro Isotype

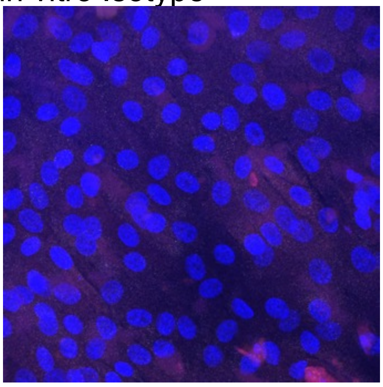

in vitro Medium control

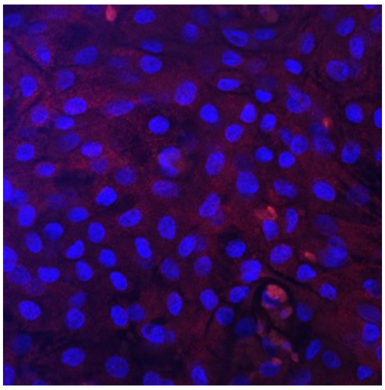

in vitro RV-A16

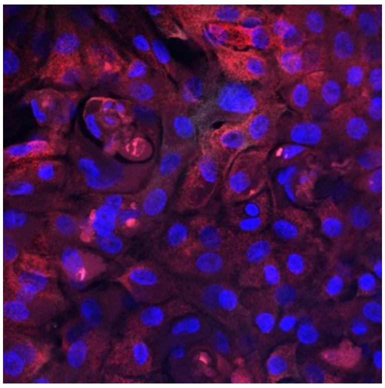

Figure 2m

Data presented: in vitro RV-A16 infection in air-liquid-differentiated primary human bronchial epithelial cells

in vitro Isotype

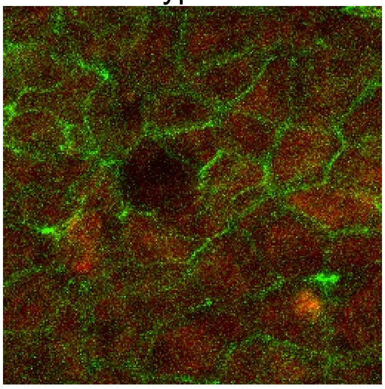

in vitro Medium control

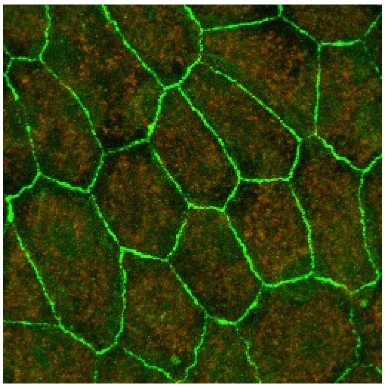

in vitro RV-A16

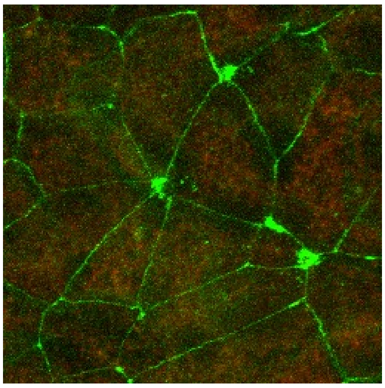

Figure 3o

Data presented: in vivo experimental RV-A16 infection in humans

Control before in vivo RV-A16 infection

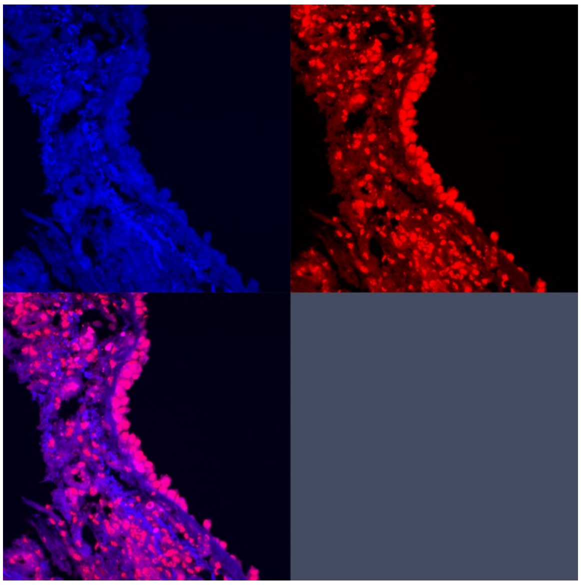

Asthma before in vivo RV-A16 infection

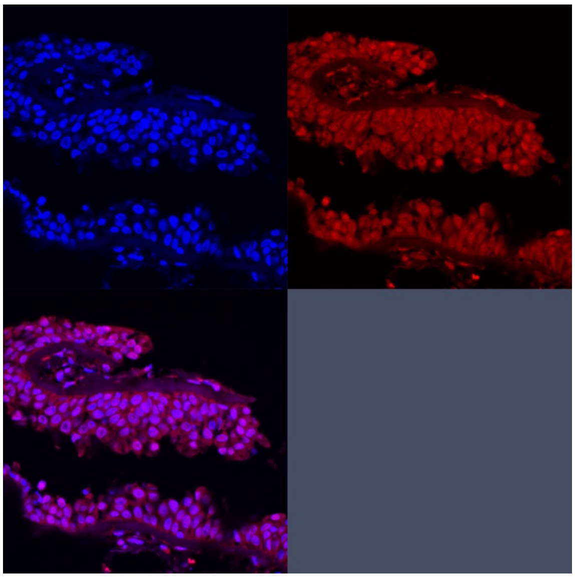

Figure 4c

Data presented: in vitro RV-A16 infection in air-liquid-differentiated primary human bronchial epithelial cells

in vitro Isotype control

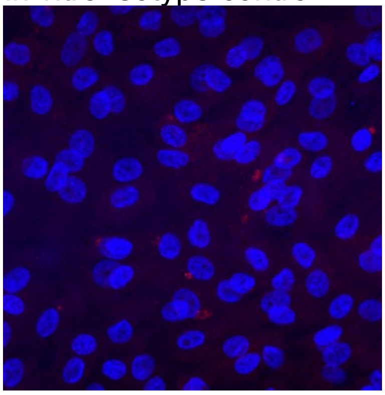

in vitro Medium control

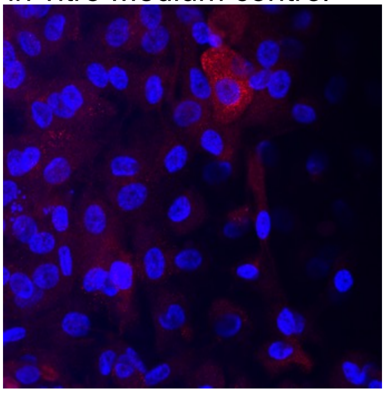

in vitro HDM

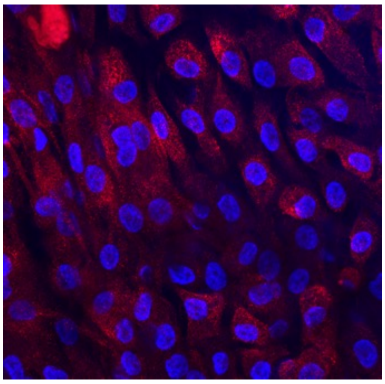

in vitro RV-A16

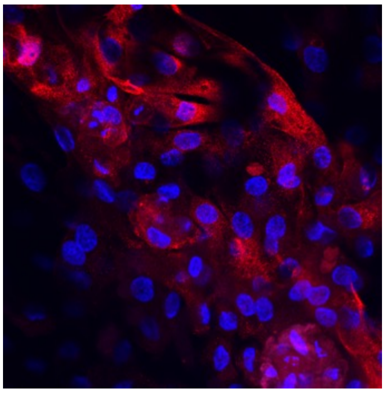

in vitro HDM+RV-A16

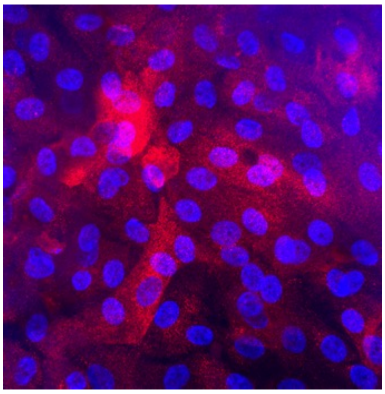

Figure 4d

Data presented: in vitro RV-A16 infection in air-liquid-differentiated primary human bronchial epithelial cells

in vitro Isotype control

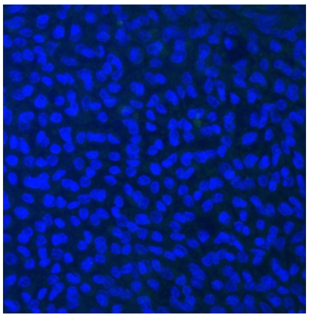

Control

in vitro RV-A16

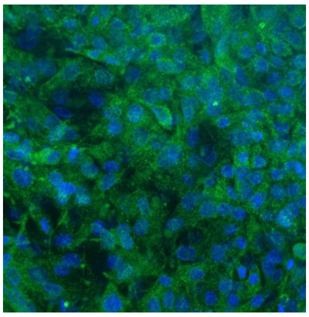

in vitro HDM

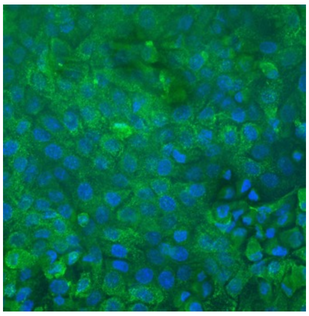

in vitro HDM+UV-RV-A16

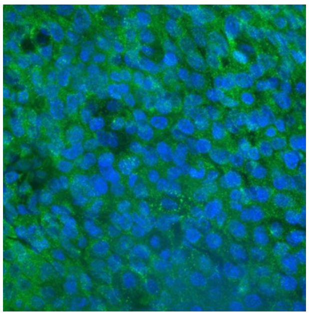

in vitro HDM+RV-A16

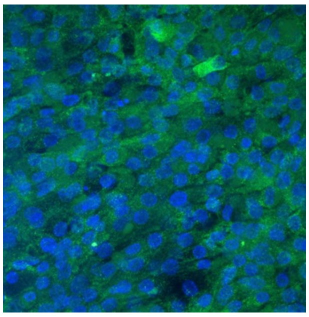

Asthma

in vitro RV-A16

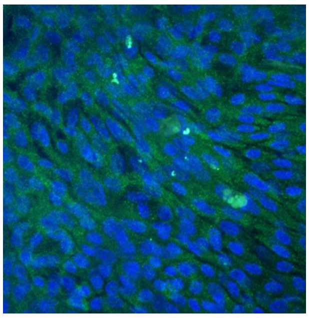

in vitro HDM

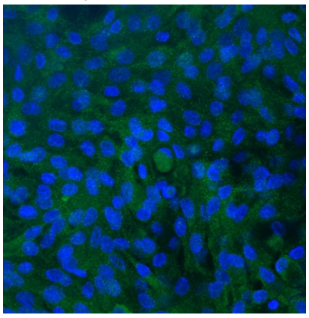

in vitro HDM+UV-RV-A16

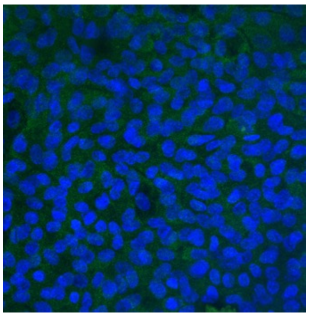

in vitro HDM+RV-A16

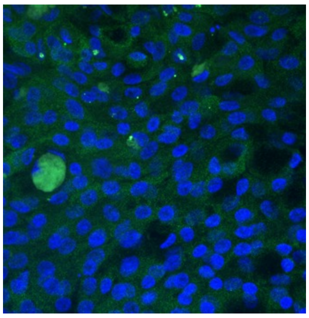

Figure 6b

Data presented: in vitro RV-A16 infection in air-liquid-differentiated primary human bronchial epithelial cells

in vitro Isotype control

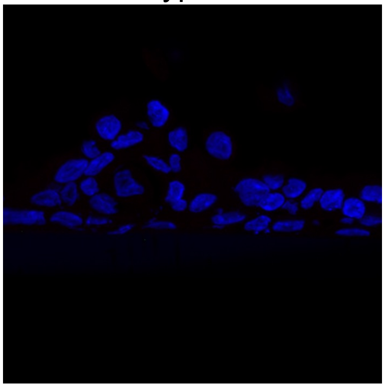

in vitro Medium control

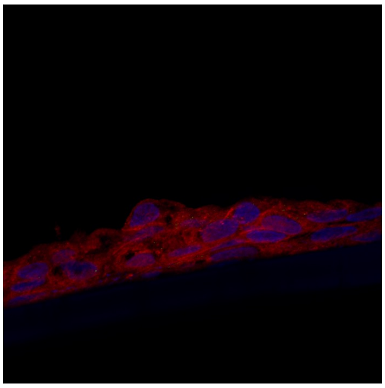

in vitro SARS-CoV-2

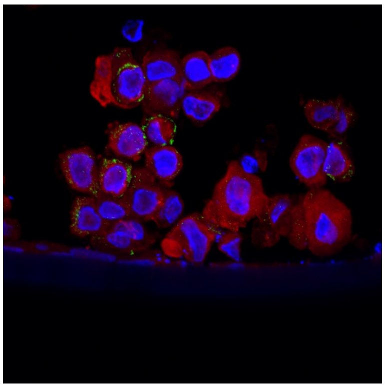

Supplement: Supplementary file 3 — Source Data [file 41467_2023_37470_MOESM3_ESM.zip › Western blots uncropped.pdf]
